# Identification of Salivary Exosome-Derived miRNAs as Potential Biomarkers of Bone Remodeling During Orthodontic Tooth Movement

**DOI:** 10.3390/ijms26031228

**Published:** 2025-01-30

**Authors:** Nikolaos Kazanopoulos, Constantinos D. Sideris, Yong Xu, Dimitrios Konstantonis, Heleni Vastardis, Elizabeth R. Balmayor, Michael Wolf, Christian Apel

**Affiliations:** 1 Department of Biohybrid & Medical Textiles, Institute of Applied Medical Engineering, RWTH Aachen University Hospital, 52074 Aachen, Germany; kazanopoulosn@gmail.com (N.K.); xu@ame.rwth-aachen.de (Y.X.); 2 Department of Biology, National and Kapodistrian University of Athens, 10561 Athens, Greece; con97sider@gmail.com; 3 Department of Orthodontics, School of Dentistry, National and Kapodistrian University of Athens, 10561 Athens, Greece; dikons@gmail.com (D.K.); vastard@dent.uoa.gr (H.V.); 4 Experimental Orthopaedics and Trauma Surgery, Department of Orthopaedic, Trauma, and Reconstructive Surgery, RWTH Aachen University Hospital, 52074 Aachen, Germany; erosadobalma@ukaachen.de; 5 Department of Orthodontics, RWTH Aachen University Hospital, 52074 Aachen, Germany; michwolf@ukaachen.de

**Keywords:** extracellular vesicles, osteogenesis, saliva, gene expression regulation, mechanical stress

## Abstract

Orthodontic tooth movement (OTM) is a complex process involving bone remodeling, and is regulated by various molecular factors, including microRNAs (miRNAs). These small, non-coding RNAs are critical in post-transcriptional gene regulation and have been implicated in the modulation of osteoclast and osteoblast activity during OTM. This study aimed to explore the expression profiles of salivary exosome-derived miRNAs during OTM to identify potential biomarkers that could provide insights into the biological processes involved in orthodontic tooth movement. Saliva samples were collected from 15 patients at three time points: before treatment (Day 0), 7 days after the treatment’s onset (Day 7), and 40 days after the treatment’s onset (Day 40). The exosomes were isolated, and the miRNAs were extracted and sequenced. A differential expression analysis and gene ontology (GO) enrichment were performed to identify the miRNAs involved in osteoblast and osteoclast differentiation. Out of the 1405 detected miRNAs, 185 were analyzed. Several miRNAs were associated with bone-remodeling processes. The statistically significant finding was the downregulation of hsa-miR-4634 after 40 days of treatment. These findings contribute to the understanding of miRNA regulation in orthodontics and may have broader implications for skeletal disorders, such as osteoporosis.

## 1. Introduction

microRNAs (miRNAs) are an integral part of gene regulatory networks, influencing a considerable number of processes both in physiological and diseased states in different tissues and organs. Alterations to their levels have been associated with many diseases and many miRNAs have been identified as biomarkers in pathological conditions [1,2,3]. miRNAs are a set of non-coding RNAs that modulate gene expression post-transcriptionally. They are, on average, 22 nucleotides long and play an important role in biological processes [3]. They target mRNAs by imperfect complementary binding, usually in the 3’ untranslated region (UTR), and they suppress their expression through a combination of translational inhibition and the promotion of mRNA decay. Their biogenesis involves their transcription by the RNA pol II as pri-miRNA and their cleavage to form pre-miRNA in the nucleus. Then, another cleavage occurs through the action of Dicer, leading to the formation of mature miRNA in a duplex form. The duplex miRNA is bound by Argonaute, thus forming the RISC complex, which is the effector of transcript downregulation [1,3].

miRNAs have been shown to be involved in processes relevant to dentistry such as tooth development, bone remodeling, and the differentiation of dental stem cells [1]. Many miRNAs are known to regulate osteoclast and osteoblast differentiation and their maintenance and function, which are important to bone remodeling. More specifically, regarding orthodontic tooth movement, miRNAs are involved in a process that triggers a series of biological changes [1,4]. In orthodontics, tooth displacement and skeletal growth modification occur due to the bone’s capacity to remodel. Orthodontic devices exert forces on the teeth and the surrounding tissues, thereby inducing reactions to their cells and the extracellular components. Bone remodeling is regulated by a balance system of two types of cells, the osteoblasts and osteoclasts, and includes a complex network of interactions between cells and between the extracellular matrix and the cells in the presence of hormones, cytokines, growth factors, and mechanical loading [3,5,6,7].

These processes heavily depend on the sterile inflammation that occurs upon the application of mechanical stress. Following the reception of mechanical cues, the signal conveying the mechanical conditions of the extracellular environment is carried towards the nucleus through MAPK kinases and, more importantly, through extracellular-signal-regulated kinases (ERKs) and c-Jun N-terminal kinases (JNKs) [8,9,10,11,12]. Then, to activate osteo-specific transcription factors like Runx2 and c-Jun, c-Fos stimulates the DNA-binding potential to specific genes associated with osteoblast differentiation (ALP, osteocalcin, collagen type I). All of the above ultimately lead to a change in gene expression and reprogramming towards the osteoblastic phenotype. Additionally, mature osteoblasts produce cytokines such as RANKL and OPG, the balance of which is essential for osteoclast differentiation and bone resorption [13,14,15,16]. The proteins essential for these processes are targeted by miRNAs. There is already a set of miRNA molecules that have been validated to be differentially expressed during orthodontic movement. These miRNAs comprise the miRNAs 21, 27, 29, 34,146, 214, and 101 [1].

Lately, the research focus in dentistry and orthodontics has been directed towards the development of biomarkers derived from oral fluids. This would be of interest because these biomarkers could provide information about the monitoring of physiological processes and serve as tools for the early diagnosis of diseases. Most studies regarding orthodontic tooth movement (OTM) have been performed on gingival crevicular fluid (GCF) [1,17,18]. GCF is the fluid between a tooth and the surrounding gingival tissues, known as gingival sulcus, and it can contain cells, biomolecules, and microbiota [19,20,21]. Apart from GCF, saliva has been identified as a potential fluid for biomarker discovery. Saliva can give a variety of information for both oral and systemic health, as its contents can alter in diseased states. Various molecules have been identified as biomarkers in saliva, in patients diagnosed with cancer or autoimmune and infectious diseases [22]. Despite the use of GCF as a biomarker source in orthodontic tooth movement, the limited sample material that can be reliably collected in the clinic, as well as the non-invasive and cost-effective collection of saliva, could make saliva a more widespread material for biomarker discovery [23].

In the context of using saliva as a source for biomarkers, there is increasing evidence regarding the utilization of salivary exosomes. Exosomes are small extracellular vehicles (EVs) (40–150 nm) secreted by a variety of cells, and they play a crucial role in intracellular communication, as they include various macromolecules such as RNAs, proteins, etc. [24,25,26]. An important feature of exosomes is that they can pass through epithelial barriers, meaning that information from the underlying tissues in the mouth can eventually be found in the saliva. This phenomenon could be leveraged in order to identify biomarkers related to systemic skeletal disorders such as osteoporosis [27,28]. The investigation of these salivary exosomes could clarify the molecular pathways associated with the bone-remodeling process, which occurs after the application of mechanical stress as in orthodontic treatment [29]. As the current literature suggests, exosomal miRNAs could be a better source for biomarker studies [30]. This study aimed to isolate salivary exosome-derived miRNAs in patients undergoing OTM and identify new biomarkers for bone remodeling.

## 2. Results

**Figure 1:**
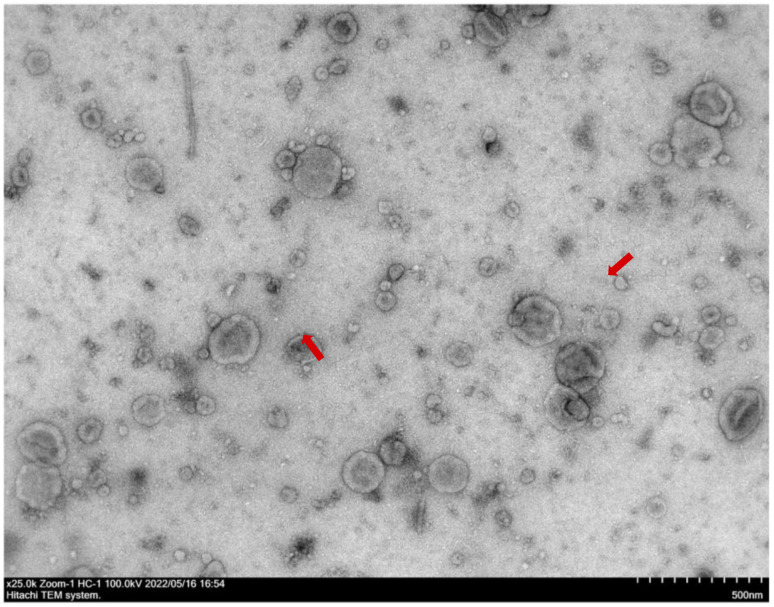
TEM image showing EVs isolated from saliva fluid pooled from multiple patient samples. The scale bar represents 500 nm. Arrows point to two representative structures.

**Figure 2:**
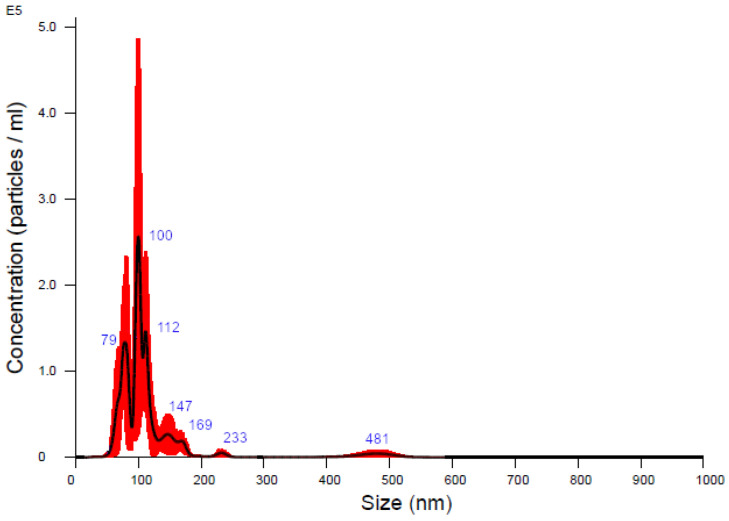
Nanoparticle tracking analysis (NTA) of EVs isolated from pooled saliva samples collected from multiple patients, presenting a size distribution plot and the mean size of each peak.

Following the isolation and analysis of salivary samples, transmission electron microscopy (TEM) confirmed the presence of EVs in the samples from patients undergoing orthodontic treatment. In the TEM analysis (Figure 1), the EVs appeared as spherical structures, with some exhibiting a distinct membrane-like structure. A complementary size distribution analysis via nanoparticle tracking confirmed that the majority of the EVs were between 100 and 150 nm in diameter, with a peak concentration around 100 nm (Figure 2). These findings confirm the successful isolation and identification of extracellular vesicles, which provide a viable source for a further miRNA analysis relevant to bone-remodeling processes during orthodontic treatment.

**Figure 3:**
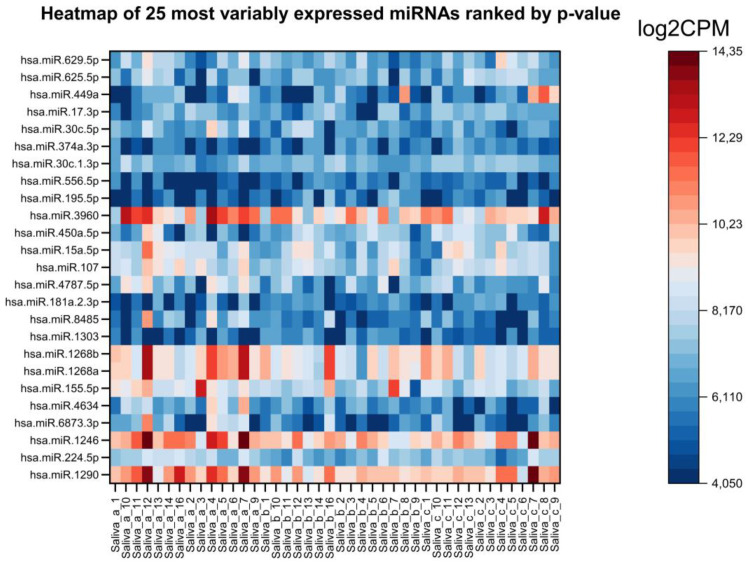
Heatmap of the 25 most variably expressed miRNAs ranked by *p*-value < 0,05. The expression levels (log2CPM) are shown across saliva samples with the miRNA. The color scale ranges from blue (low expression) to red (high expression).

**Figure 4:**
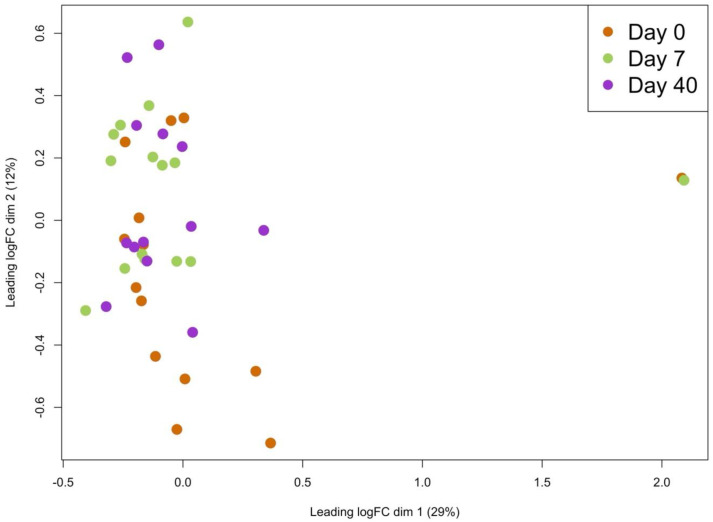
MDS plot showing the separation of samples across three time points (Day 0, Day 7, and Day 40) based on the log fold change (logFC). Each point represents a sample, with the colors indicating the corresponding time points: orange for Day 0, green for Day 7, and purple for Day 40. The leading dimensions 1 and 2 explain 29% and 12% of the variance, respectively.

The most variably expressed miRNAs can be viewed in the heatmap below (Figure 3), with no distinct clustering observed in either the samples or the miRNA expression patterns. Likewise, the MDS plot (Figure 4) did not display distinct groupings based on time points or individual subjects, suggesting an absence of strong time-dependent or subject-specific patterns in the data.

**Figure 5:**
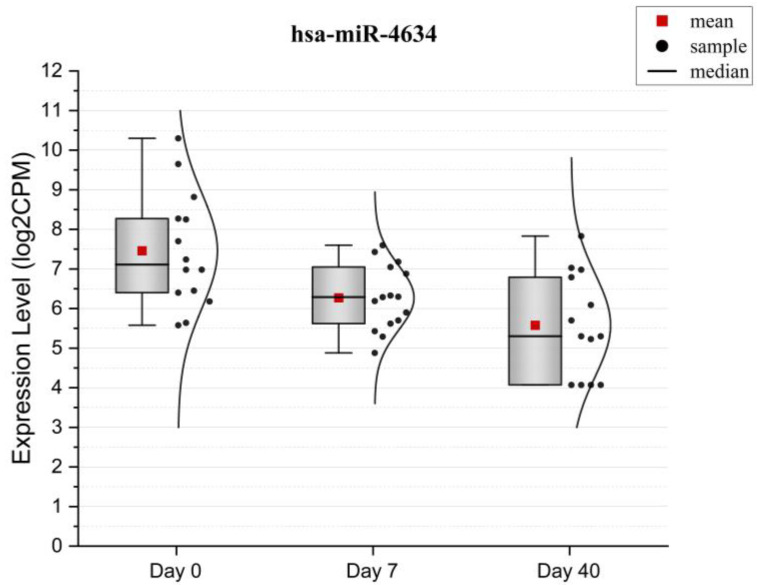
Boxplot of hsa-miR-4634 expression levels (log2 CPM) at three time points (Day 0, Day 7, and Day 40). The red square represents the mean expression level, and the black dots represent individual samples. The black line in each box indicates the median value, and the density plot on each side shows the distribution of expression values.

Of the comparisons made, the Day 40 vs. Day 0 contrast yielded a statistically significant result, identifying hsa-miR-4634 as differentially expressed, with its expression reduced on Day 40 compared to Day 0 (log fold change = −1.9, mean expression = 6.2 log2-transformed CPMs, adjusted *p*-value = 0.043) (Figure 5).

**Figure 6:**
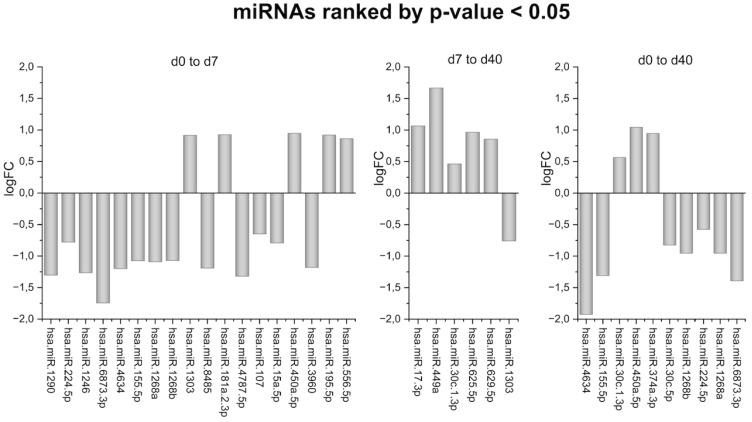
Bar plots showing the log fold changes (logFC) of miRNAs ranked by *p*-value < 0.05 across different time point comparisons: Day 0 to Day 7, Day 7 to Day 40, and Day 0 to Day 40. Each bar represents an miRNA, with positive and negative values indicating upregulation and downregulation, respectively, between the compared time points. The data presented represent the combined analysis of all the included samples rather than individual patient data.

While other miRNAs did show notable changes in expression, they did not reach statistical significance after adjusting for multiple testing to reduce the likelihood of false positives (adjusted *p*-value > 0.1). Because these miRNAs changed in a statistically significant way (*p*-value < 0.05), it is noteworthy to mention and analyze their alterations (Figure 6). Using the database TarBase-v9.0, we generated a list of the genes that these miRNAs target [31]. Afterwards, we performed a gene ontology (GO) enrichment analysis using the bioinformatics tool DAVID (database for annotation, visualization, and integrated discovery) [32]. Of all the processes affected by the input gene set, we searched only the processes that are the most relevant to bone remodeling, such as osteoblast differentiation (GO:0001649); osteoclast differentiation (GO:0030218); the positive regulation of osteoblast differentiation (GO:0045669); and the positive regulation of bone mineralization (GO:0030501) (Table 1, Table 2 and Table 3).

## 3. Discussion

The present study is the first, to our knowledge, to investigate the expression of miRNAs in salivary exosomes during OTM in patients. The foremost finding of this study concerns miRNA hsa-miR-4634, which was found to have a statistically significant altered expression. Its expression was downregulated after 40 days of treatment. The literature regarding this particular miRNA is limited. Still, the only known target for this miRNA is VAV3 [33]. VAV3 is a Rho family guanine nucleotide exchange factor, which has been shown to be essential for stimulated osteoclast activation and bone density. Guanine nucleotide exchange factors (GEFs) mediate the activation of Rho family GTPase by exchanging GDP for GTP. This occurs in addition to GTPase activation, thus influencing downstream signaling pathways. VAV3 has been identified as an essential factor in the regulation of osteoclast function. More specifically, VAV3-deficient mice were shown to have an increased bone mass because of dysfunctional osteoclasts and exhibited protection from stimulated bone loss. Also, the authors reported that GTPase Rac1 was affected by defective VAV3, and the authors concluded that the activation of Rac1 is VAV3-dependent and the signaling after cytokine stimulation required for cytoskeletal reorganization in osteoclasts is impaired [34]. Based on the findings of the aforementioned study, it is evident that the regulation of VAV3 could be significant in orthodontic tooth movement, in the phase where osteoclast activity is essential for bone remodeling. It is possible that, in this setting, hsa-miR-4634 is downregulated in order for an upregulation of VAV3 to occur, a hypothesis that could be addressed in a future study with the use of experimental models.

Apart from miR-4634, which is statistically significant for the adjusted *p*-value, some noteworthy results can be extracted from the other miRNAs with a *p*-value < 0.05, such as hsa-miR-195-5p and hsa-miR-1246, which are known to regulate osteoblast differentiation and inhibit the osteogenic potential of progenitor cells, respectively (Figure 6) [35]. The GO enrichment analysis revealed that processes associated with bone remodeling were impacted, including osteoblast differentiation, the positive regulation of osteoblast differentiation, osteoclast differentiation, and the positive regulation of bone mineralization. A comparison of the three tables (Table 1, Table 2 and Table 3) yielded significant data. The first statistical comparison was of the differential expression of miRNAs on Day 7 vs. Day 0, the second was of the expression on Day 40 vs. Day 7, and the third was of the expression on Day 40 vs. Day 0. What might be observed is that the first comparison showed genes related to processes of osteoblast differentiation, the positive regulation of osteoblast differentiation, and bone mineralization, while the other comparisons included osteoclast differentiation as well.

OTM can be divided into three phases: the initial phase, characterized by rapid tooth movement immediately after force application; the lag phase, during which tooth movement temporarily halts due to the hyalinization of the periodontal ligament; and the post-lag phase, when movement resumes as necrotic tissue is cleared, allowing the tooth to continue its displacement [36]. In our study, the selected timepoints corresponded to the initial (0 to 7 days) and the lag and post-lag phases (7 to 40 days). Current reports in the literature suggest that, in the initial phase after the application of mechanical stress, the early cellular response involves inflammation and tissue remodeling. Osteoclastogenesis, a critical process for bone resorption, tends to increase during the later stages of tooth movement, which aligns with our findings [37,38]. The absence of detectable miRNAs targeting osteoclast differentiation genes early on can be attributed to the fact that osteoclast activity is still relatively low during this phase [39].

In the lag and post-lag phases of orthodontic tooth movement, after the application of stress, alveolar bone remodeling becomes more pronounced as osteoclasts are activated on the pressure side of the tooth [39,40]. At this phase, miRNAs related to osteoclast differentiation, such as those targeting the RANK, RANKL, or NFATc1 pathways, are more likely to be expressed [37,40]. This molecular phase corresponds clinically with the acceleration phase of tooth movement, when bone resorption becomes more critical, facilitating tooth displacement through the alveolar bone. These reports are in accordance with our results, as in the initial phase of our research, only osteoblast differentiation and the positive regulation of osteoblast differentiation were affected, while osteoclast differentiation was affected at later time points [38,41]. The detection of differentially expressed miRNAs in this final phase could be a result of the crosstalk of the periodontal ligament cells and the cells of the alveolar bones. The cellular source of EVs in the saliva is heterogenous, as various cell types can contribute [42]. Apart from the epithelial cells and immune cells of the oral cavity, a portion of the EVs found in the saliva can originate from dental-tissue-derived cells, such as gingival mesenchymal stem cells and periodontal ligament stem cells [43]. The periodontal ligament is mechanically stimulated during orthodontic tooth movement; it is the primary tissue that responds to mechanical signal transduction, and it conveys changes in the surrounding bone [44]. As it is known that periodontal ligament stem cells (PDLSCs) change their EV miRNA content in response to mechanical stress in order to affect the activity of osteoblasts and osteoclasts [45], and preventing the release of EVs from PDLSCs results in a disrupted osteoclast function in OTM [46], one could hypothesize that the changes in the expression of miR-4634 and the other differentially expressed miRNAs in our study could be reflective of the response of the periodontal ligament during OTM.

Summarizing the results of this study, the most significant finding was the downregulation of the miRNA hsa-miR-4634. This downregulation was found to occur on Day 0 to Day 40 of tooth movement. Even though there might be a plausible biological explanation for their altered expression that fits the setting of orthodontic stress application, none of the aforementioned miRNAs (Table 1, Table 2 and Table 3) were found to be significant after the *p*-value adjustment.

One of the potential limitations of this study is that the sample comprised patients in the first phase of orthodontic treatment, during which only a small number of teeth were moved. The cellular and molecular changes expected to occur might have been more pronounced if mechanical stress had been exerted on a larger number of teeth. It is important to note that the term saliva is used interchangeably with oral fluid. As the samples were collected after the patients chewed on parafilm, saliva production was stimulated [47]. Because of this, the collected fluid was oral fluid that contained mainly saliva, but also other components of the oral cavity. Moreover, the flow rate was not recorded, but at least 5 min were required to produce 5 mL of saliva. This corresponds to a stimulated saliva flow rate of approximately 1 mL/min and aligns with the expectations in healthy adolescents [48].

This study might serve as a starting point in investigating altered oral fluid miRNA expression during orthodontic treatment. Further studies are needed to elucidate the molecular interplay between miRNAs and their targets. The identification of potential biomarkers will be of great value to clinical orthodontics and future oral therapeutics. In molecular orthodontics, the clinician may be ultimately capable of controlling several clinical aspects, like the rate of tooth movement. The decoding of the human genome, along with new developments in molecular biology, has provided a much-anticipated boost to the biological sciences. Orthodontics, which increasingly relies on biotechnology, is expected to be significantly impacted by these advancements.

## 4. Materials and Methods

### 4.1. Patients

This study received ethical approval from the ethical committee of the RWTH Medical University of Aachen in Germany, and informed consent was obtained from all the participants (ethical approval number: CTC-A-Nr.: 22-262).

This study involved fifteen Caucasian patients aged 11–15 years (six females with a mean age of 14 ± 2.3 years and nine males with a mean age of 13 ± 1.3 years) presenting with a dental Class I or Class II malocclusion accompanied by moderate to severe crowding or spacing. When comparing the mean age of male and female patients, there were no statistically significant differences between the two groups (*p*-value = 0.583), suggesting that gender did not have a significant effect on patient age. These patients were treated in a private orthodontic office in Bedburg, Germany. The age group used for this study was selected in order to minimize the likelihood of the presence of underlying conditions, such as periodontitis or systemic disorders, that could confound the results. The exclusion criteria included any history of cleft lip/palate, dentofacial deformities or syndromes, autoimmune diseases, or type 1 or type 2 diabetes; a history of drug use; and previous orthodontic treatment or intraoral/external oral surgery. All the patients underwent a thorough intraoral examination, a clinical assessment of the teeth, and a periodontal evaluation, which included the approximal plaque index (API), sulcus bleeding index (SBI), and periodontal screening index (PSI) [49,50,51] (Table S1—Supplementary Materials). According to the orthodontic treatment plan, the 4 maxillary incisors and the 2 first maxillary molars were bonded with fixed pre-adjusted orthodontic appliances featuring a 0.22 slot size. From each patient, saliva samples were collected at three different time points: one week before bracket placement and archwire activation, 7 days after the treatment’s onset, and 40 days after the treatment’s onset. Thus, 45 saliva samples were finally collected.

### 4.2. Sample Size Calculation

This study was designed as an exploratory analysis, with the primary objective of examining temporal changes in biomarkers, without testing predefined hypotheses. The sample size was determined through a power analysis based on a repeated measures design, where three measurements were collected from each participant at distinct time points: prior to bracket placement (Day 0), one week after the treatment (Day 7), and 40 days after the treatment (Day 40). Based on previous studies, a medium effect size (f = 0.30) was observed for changes in biomarkers over time [52]. A power analysis was performed using the pwr package in R (Table S2—Supplementary Materials), which indicated that a minimum of 15 participants was required to achieve 40% power at an alpha level of 0.05 for detecting differences across the three time points. In exploratory analyses, the use of a lower power can be justified when the primary objective is to identify preliminary trends that may guide the design of future, more rigorous studies [53]. Accordingly, 15 patients were recruited for this study, and saliva samples were collected from each participant at the three time points, resulting in a total of 45 samples.

### 4.3. Saliva Collection

The saliva samples were collected in the dental office where the patients received orthodontic treatment. The collection was placed on ice in Falcon tubes previously refrigerated. The participants were instructed not to brush their teeth, chew gum, eat, or drink any liquids for at least 1.5 h before the visit. Saliva was collected upon arrival by having the patients chew a piece of parafilm for one minute while swallowing normally. The time of collection was set as late afternoon (15:00–18:00) for all the patients in order to prevent potential time-dependent changes in the saliva content. Before collection, 100 μL of PhosSTOP™ EASYpack (Roche Applied Science, Cat. No. 04 906 845 001, Mannheim, Germany) and 100 μL of cOmplete™ Mini Protease Inhibitor Cocktail Tablets (Roche Applied Science, Cat. No. 04 693 124 001, Mannheim, Germany) were added to a cold 50 mL Falcon tube. This was equivalent to ½ tablet of PhosSTOP and ½ tablet of cOmplete. The participants were instructed to begin chewing and then hold the saliva in their mouth (i.e., not swallow), and at 30-s intervals, eject the saliva into the cold 50 mL Falcon tube. The participants continued this process until a minimum of 5 mL had been obtained, or for up to 15 min of chewing and ejecting the saliva.

After collection, the saliva samples were diluted with pre-cooled phosphate-buffered saline (PBS) at a ratio of 1:1. The samples were then centrifuged (Sigma 3K15, Sigma, Osterode am Harz, Germany) at 300× *g* for 20 min at 4 °C and the pellet was removed. Afterward, the supernatant was centrifuged further at 2000× *g* for 10 min at 4 °C, and the resulting pellet was discarded. This step was repeated at 5000× *g* for 30 min at 4 °C, and the final pellet was discarded. The remainder of the supernatant was stored at 4 °C until same-day transport and then stored at −80 °C in the lab.

At the lab, the supernatant was thawed and centrifuged using an Optima LE-80K ultracentrifuge with an SW 32 Ti rotor (Beckman Coulter, Chaska, MN, USA) at 12,000× *g* for 20 min at 4 °C; then, the pellet was discarded. The sample was centrifuged at 120,000× *g* for 70 min at 4 °C, after which the supernatant was discarded. The pellet was resuspended, filtered using a 0.2 μm filter, and washed again by centrifugation at 120,000× *g* for 70 min at 4 °C. The final pellet was resuspended in filtered PBS and stored at −80 °C for long-term storage [24,25,54,55].

### 4.4. RNA Isolation

RNA was isolated from 60 µL of exosome samples using the Maxwell RSC miRNA Plasma and Serum Kit (Promega, Madison, WI, USA) according to the manufacturer’s instructions. This process ensured the efficient extraction of small RNAs, including miRNAs, from the exosome samples.

### 4.5. RNA Quantification

The concentration of the isolated RNA was determined using a Promega Quantus Fluorometer (Promega, Madison, WI, USA). This quantification step was crucial to ensure sufficient RNA input for downstream library preparation.

### 4.6. Library Preparation

Sequencing libraries were prepared using the QIAseq miRNA UDI Library Kit (QIAGEN, Hilden, Germany), following the manufacturer’s protocol. QIAseq miRNA Library QC spike-ins were added to each sample to monitor the library preparation process and ensure the accuracy of miRNA detection. This step facilitated the normalization and validation of the library preparation efficiency.

### 4.7. Library Quality Control

The size distribution of the prepared libraries was assessed using the Agilent TapeStation (Agilent Technologies, Santa Clara, CA, USA). The average library size was confirmed to fall within the expected range for miRNA libraries. After size confirmation, the library concentrations were determined with the Promega Quantus Fluorometer to ensure accurate loading for sequencing.

### 4.8. Sequencing

Sequencing was performed on an Illumina NextSeq 500/550 system using a High Output Kit v2.5 (75 cycles) (Illumina Inc., San Diego, CA, USA). The libraries were sequenced in a single-end mode for 72 cycles, with a 1% PhiX spike-in used as an internal control to monitor sequencing quality and performance.

### 4.9. Statistical and Bioinformatical Analysis

FASTQ files were generated using bcl2fastq (Illumina). To facilitate a reproducible analysis, the samples were processed using the publicly available nf-core/smRNAseq pipeline, version 1.1.0 [56], implemented in Nextflow 21.10.6 [57] using Docker 20.10.12 (Merkel 2014) with the minimal command.

Out of 45 samples, 1 was eliminated from further analysis because no miRNA could be detected in the sample. Therefore, miRNA counts from the 44 samples were analyzed to identify changes in the expression levels. Two samples with library sizes (sum of miRNA counts) of less than 10,000 were excluded, resulting in 42 samples for analysis. Such a phenomenon could be a result of the various centrifugation and washing steps involved in the process of purification. Out of the 1405 miRNAs, only those with at least one read in a minimum of five samples were retained, yielding 185 miRNAs. The miRNA expression table in TMM-normalized CPMs, after count-based miRNA filtering, is included in the Supplementary Materials (Table S3—Supplementary Materials).

To assess the overall structure and quality of the data before conducting a differential expression analysis, a multidimensional scaling (MDS) plot was generated using the plotMDS function from the “limma” R package. In this plot, each point represents a sample, with the distance between points indicating the similarity of their miRNA expression profiles.

The differential expression analysis was performed using the “limma” R package once again. The “voom” function from the “limma” R package was used to transform the count data into log2-counts per million (CPM) with associated weights, accounting for mean–variance relationships. Next, a linear model was fitted to the transformed data while accounting for within-subject correlations. The “duplicateCorrelation” function was employed to estimate the correlation between measurements from the same subject, with the inter-subject correlation being incorporated into the linear model fitting process. This correlation was subsequently used in the “lmFit” function, which fitted the linear model to the data, adjusting for the block effect (i.e., subject-specific variation). To compare different time points, contrasts were defined using the “makeContrasts” function for the following comparisons: Day 7 vs. Day 0, Day 40 vs. Day 7, and Day 40 vs. Day 0. These contrasts were applied to the fitted model using the “contrasts.fit” function. Finally, moderated *t*-tests were computed using the “eBayes” function to determine the significance of differential expression across the specified contrasts. The R script for this analysis is provided in the Supplementary Materials (Table S3—Supplementary Materials).

## 5. Conclusions

The downregulation of hsa-miR-4634 may play a role in modulating osteoclast function, possibly through the regulation of the gene VAV3. The findings suggest that salivary miRNAs, particularly those derived from exosomes, hold promise as non-invasive biomarkers for monitoring bone-remodeling processes during orthodontic tooth movement. Although other miRNAs showed changes in expression, they did not reach statistical significance after adjustment, highlighting the complexity of miRNA regulation in response to mechanical stress. Future research should focus on elucidating the molecular interactions between miRNAs and their targets, which may provide valuable insights not only for advancing orthodontic treatments, but also for enhancing our understanding of broader skeletal health issues, such as osteoporosis. Understanding these molecular mechanisms could eventually lead to more personalized approaches in orthodontics, including the modulation of tooth movement rates based on individual miRNA signatures.

## Figures and Tables

**Table 1:** Initial phase—d0 to d7: Summary table for the comparison of miRNAs from the initial phase (Day 0 to Day 7) following the GO enrichment analysis. The listed target genes are involved in osteoblast differentiation, the positive regulation of osteoblast differentiation, and the positive regulation of bone mineralization.

miRNAs	Osteoblast Differentiation	Positive Regulation of Osteoblast Differentiation	Positive Regulation of Bone Mineralization
**hsa-miR-107**	ADAP2, ALS2, ALS2CL, ARHGAP12, BVES, CRK, DAB2IP, EFNA5, EPHA1, EPHA3, EPHA4, EPHA5, EPHB3, FGD3, FGD4, ITGB1BP1, NF1, NTRK2, PLXNA1, PLXNA2, PLXNA3, PLXNA4, PLXNB2, PLXNB3, PLXND1, PRKG1, PROM2, PTK2, RAB3GAP1, RAB3GAP2, RABGAP1, RABGAP1L, RALBP1, RAP1GAP, RAPGEF6, RDX, SIPA1L1, SOD1, STMN3, TBC1D10B, VAV2, VAV3	ACVR1, ACVR2A, ACVR2B, ATRAID, BMP2, BMPR1A, BMPR1B, BMPR2, CCN1, CEBPB, CTHRC1, CTNNB1, CTNNBIP1, FAM20C, FBN2, FBXO5, FERMT2, FGF2, HGF, IFITM1, IGF1, IL6, IL6ST, ILK, JAG1, JUND, LRP5, PPP3CA, SMAD1, SMAD5, SOX11, SUCO, TENT5A, TP63, VEGFA, WNT10B, WNT3, WNT7B, WWTR1, YAP1, ZHX3	ACVR1, ACVR2A, ACVR2B, ADRB2, ANO6, ATP2B1, ATRAID, BMP2, BMPR1A, BMPR1B, BMPR2, CCN1, FAM20C, FBN2, GPM6B, OSR1, OSR2, RXRA, SLC20A2, SLC8A1, SMAD3, TENT5A, TFAP2A, VDR, WNT10B
**hsa-miR-1246**		CEBPB, IL6ST, SMAD5, WWTR1	ATP2B1
**hsa-miR-1290**	ARHGAP12, CRK, FGD4, RABGAP1, RALBP1	IL6ST, SUCO	
**hsa-miR-1303**	NF1	JAG1, PPP3CA	
**hsa-miR-155-5p**	ARHGAP12, CRK, EPHA4, EPHA5, EPHB3, NF1, NTRK2, PLXNA2, PLXNA3, PLXNB1, PLXND1, RAB3GAP1, RABGAP1, RABGAP1L, RAPGEF6, RDX, TBC1D15, VAV2	ACVR2A, ACVR2B, BMP4, BMP6, BMPR1A, BMPR2, CCN1, CEBPB, CEBPD, CTNNB1, FBXO5, FERMT2, FGF2, FZD1, HGF, IFITM1, IGF1, IL6, IL6R, IL6ST, ILK, JAG1, MEF2C, RUNX2, SMAD1, SMAD5, TP63, VEGFA, WWTR1, YAP1	ACVR2A, ACVR2B, ADRB2, ATP2B1, BMP4, BMP6, BMPR1A, BMPR2, CCN1, GPM6B, ISG15, MEF2C, RXRB, SMAD3, TFAP2A
**hsa-miR-15a-5p**	ADAP1, ADAP2, ALS2, ALS2CL, ARHGAP12, ARHGAP44, ARHGAP9, ARHGEF5, BVES, CRK, DAB2IP, EFNA5, EPHA1, EPHA3, EPHA4, EPHA5, EPHB3, FGD1, FGD3, FGD4, FGD5, FGD6, GPR137B, ITGB1BP1, NF1, PLXNA1, PLXNA2, PLXNA3, PLXNA4, PLXNB1, PLXNB2, PLXNB3, PLXNC1, PLXND1, PRKG1, PROM2, PTK2, RAB3GAP1, RAB3GAP2, RABGAP1, RABGAP1L, RALBP1, RAP1GAP, RAPGEF6, RASIP1, RDX, SIPA1L1, SOD1, STMN3, TBC1D10B, TBC1D15, VAV2, VAV3	ACVR1, ACVR2A, ACVR2B, ATRAID, BMP2, BMP6, BMPR1A, BMPR1B, BMPR2, CCN1, CEBPB, CTHRC1, CTNNB1, CTNNBIP1, DDR2, FAM20C, FBN2, FBXO5, FERMT2, FGF2, FZD1, GLI3, HGF, IFITM1, IGF1, IL6, IL6R, IL6ST, JAG1, JUND, LRP3, LRP5, MEF2C, MSX2, NPNT, PPP3CA, RUNX2, SCUBE2, SCUBE3, SFRP2, SMAD1, SMAD5, SOX11, SUCO, TENT5A, VEGFA, WNT10B, WNT3, WNT7B, WWTR1, YAP1, ZHX3	ACVR1, ACVR2A, ACVR2B, ADGRV1, ADRB2, ANO6, ATP2B1, ATRAID, BMP2, BMP6, BMPR1A, BMPR1B, BMPR2, CCN1, FAM20C, FBN2, GPM6B, KL, MEF2C, OSR2, P2RX7, PKDCC, RXRA, RXRB, SLC20A2, SLC8A1, SMAD3, TENT5A, TFAP2A, VDR, WNT10B
**hsa-miR-181a-2-3p**	ARHGAP9, CRK, PLXNA2, PLXNA4, PLXND1, RABGAP1, RAPGEF6, SIPA1L1, TBC1D15	ACVR2A, ACVR2B, BMPR2, CTNNB1, CTNNBIP1, FERMT2, JAG1, LRP5, PPP3CA, SMAD5, SOX11, SUCO, VEGFA, YAP1	ACVR2A, ACVR2B, BMPR2, RXRB
**hsa-miR-195-5p**	ALS2, ALS2CL, ARHGAP12, BVES, CRK, EFNA5, EPHA1, EPHA4, EPHA5, FGD1, NF1, PLXNA1, PLXNA3, PLXNB2, PLXNB3, PLXND1, PRKG1, PTK2, RAB3GAP1, RAB3GAP2, RABGAP1, RABGAP1L, RAP1GAP, SIPA1L1, SOD1, STMN3, TBC1D10B, TBC1D15, VAV2, VAV3	ACVR2A, ACVR2B, BMPR2, CCN1, CEBPB, CTNNB1, CTNNBIP1, DDR2, FBN2, FBXO5, FERMT2, FGF2, FZD1, HGF, IGF1, IL6R, IL6ST, LRP3, LRP5, MEF2C, NPNT, PPP3CA, SMAD1, SMAD5, SOX11, SUCO, TENT5A, VEGFA, WNT3, WNT7B, WWTR1, YAP1, ZHX3	ACVR2A, ACVR2B, ADGRV1, ADRB2, ANO6, ATP2B1, BMPR2, CCN1, FBN2, MEF2C, OSR1, OSR2, RXRA, RXRB, SLC20A2, SMAD3, TENT5A, TFAP2A, VDR
**hsa-miR-224-5p**	CRK, NTRK2, SOD1	ATRAID, BMPR2, CTNNB1, FERMT2, IL6R, IL6ST, JUND, PPP3CA, SMAD5, SUCO, TENT5A, YAP1	ATRAID, BMPR2, SLC20A2, TENT5A, VDR
**hsa-miR-450a-5p**	BVES, DAB2IP, EPHB3, FGD1, FGD6, NF1, PLXNA2, PLXNB2, PLXNC1, PLXND1, RABGAP1, RALBP1, RDX	ACVR1, ATRAID, BMP2, BMPR1A, BMPR2, CCN1, CEBPD, CTNNB1, DDR2, FAM20C, FBN2, FERMT2, FGF2, HGF, JAG1, JUND, MEF2C, SFRP2, SUCO, TENT5A, TP63, VEGFA, WWTR1, YAP1, ZHX3	ACVR1, ATRAID, BMP2, BMPR1A, BMPR2, CCN1, FAM20C, FBN2, ISG15, MEF2C, PTN, RXRA, SLC20A2, SMAD3, TENT5A, TFAP2A
**hsa-miR-8485**		ILK	

**Table 2:** Lag and post-lag phases—d7 to d40: Summary table for the comparison of miRNAs from the lag and post-lag phases (Day 7 to Day 40) following the GO enrichment analysis. The target genes mentioned are associated with osteoblast differentiation, the positive regulation of osteoblast differentiation, and osteoclast differentiation.

miRNAs	Osteoblast Differentiation	Positive Regulation of Osteoblast Differentiation	Osteoclast Differentiation
**hsa-miR-1303**	DDX21, GJA1, NF1, SEMA7A, SMAD4	JAG1, PPP3CA	FOXP1, NF1, PIK3R1, TFRC
**hsa-miR-17-3p**	AKT1, BMPR2, CLTC, CTNNB1, DHX9, FASN, GABBR1, HIRA, HNRNPC, HNRNPU, LGR4, MAPK14, MEF2D, NF1, RPS15, RRBP1, RSL1D1, SMAD4, SMAD5, SNAI1, SND1, SNRNP200, SYNCRIP, TNC, UFL1, VCAN	ACVR1, ACVR2A, ACVR2B, BMPR2, CTNNB1, HGF, IGF1, IL6R, IL6ST, JAG1, LRP3, LRP5, PPP3CA, SMAD5, YAP1	CSF1, CSF1R, CTNNB1, FARP2, FOXP1, GLO1, MAPK14, NF1, SLC4A2, TFRC, TOB2
**hsa-miR-30c-1-3p**	ADAR, ATP5F1B, BMP2, BMPR1B, BMPR2, CAT, CBFB, CLTC, COL6A1, CREB3L1, CTNNB1, DDX21, DHX9, EPHA2, FASN, FBL, FGF2, FOSL2, GJA1, GLI3, GTPBP4, HIRA, HNRNPC, HNRNPU, IGFBP5, JUNB, LGR4, MAPK14, MEF2D, NF1, RDH14, RRBP1, RSL1D1, RUNX2, SEMA7A, SMAD4, SMAD5, SNAI2, SND1, SNRNP200, TNC, TP53INP2, UFL1, WWTR1	ACVR2B, ATRAID, BMP2, BMPR1B, BMPR2, CLIC1, CTNNB1, DDR2, FERMT2, FGF2, GLI3, IL6R, IL6ST, ILK, JAG1, JUND, LRP5, NPNT, PPP3CA, RUNX2, SMAD1, SMAD5, SUCO, TENT5A, WWTR1, YAP1	BMP2, CSF1, CTNNB1, EPHA2, FOSL2, FOXP1, GLO1, IREB2, JUNB, MAPK14, MITF, NF1, PIK3R1, SBNO2, SLC4A2, TFRC, TNF, TOB2
**hsa-miR-449a**	ADAR, AKT1, ALPL, ATP5F1B, AXIN2, BMP4, BMPR1A, BMPR2, CAT, CCDC47, CLTC, DDX21, DNAJC13, EPHA2, FASN, FHL2, FIGNL1, GLI3, HIRA, HSPE1, IARS1, IGFBP3, IGFBP5, ITGA11, LGR4, MAPK14, MSX2, NF1, RSPO2, SEMA7A, SMAD4, SND1, SNRNP200, TNC, TP53INP2, VCAN	ACVR1, ATRAID, BMP4, BMPR1A, BMPR2, CEBPA, CEBPB, FBN2, FERMT2, GLI3, IL6R, ILK, JAG1, MSX2, NPNT, NPPC, SOX11, SUCO, VEGFA, YAP1	EPHA2, FOS, MAPK14, NF1, OSTM1, PIK3R1, TOB2, TYROBP
**hsa-miR-625-5p**	ADAR, AKT1, BCAP29, BMP2, BMPR2, CBFB, CLTC, COL1A1, COL6A1, CTNNB1, DDX21, DNAJC13, EPHA2, FASN, FBL, FGF2, FOSL2, GABBR1, GTPBP4, HAND2, HIRA, HNRNPC, HNRNPU, MEF2D, NF1, RBMX, RPS15, RUNX2, SEMA7A, SMAD4, SMAD5, SNRNP200, SYNCRIP, VCAN	ACVR1, ACVR2B, BMP2, BMPR2, CEBPB, CLIC1, CTNNB1, DDR2, FERMT2, FGF2, IGF1, JAG1, PPP3CA, RUNX2, SMAD5, VEGFA, WNT3, YAP1	BMP2, CREB1, CTNNB1, EPHA2, FOS, FOSL2, FOXP1, GLO1, IREB2, MITF, NF1, SBNO2, SLC4A2, TCIRG1, TFRC, TOB2, TRAF6
**hsa-miR-629-5p**	ATP5F1B, BMP2, BMPR2, CLTC, COL1A1, CTNNB1, DDX21, FASN, FOSL2, HNRNPU, IFT80, IGFBP5, LGR4, MEF2C, MEF2D, PSMC2, RSL1D1, SEMA7A, SMAD5, SNRNP200, VCAN	ACVR2A, BMP2, BMPR2, CEBPB, CTNNB1, FERMT2, MEF2C, PPP3CA, SMAD5, YAP1	BMP2, CTNNB1, FOSL2, FOXP1, PIK3R1, TMEM64

**Table 3:** Day 0 to Day 40: Summary table for the comparison of miRNAs from Day 0 to Day 40 following the GO enrichment analysis. The listed target genes are related to osteoblast differentiation, the positive regulation of osteoblast differentiation, osteoclast differentiation, and the positive regulation of bone mineralization.

miRNAs	Osteoblast Differentiation	Positive Regulation of Osteoblast Differentiation	Osteoclast Differentiation	Positive Regulation of Bone Mineralization
**hsa-miR-1268a**	CDK12, POLR2D, SCAF1			
**hsa-miR-1268b**	CCNT1, IRF9, SCAF1			
**hsa-miR-155-5p**	ABT1, ADRB2, ARID4A, ASH1L, ATRX, ATXN1L, BCL9, BMP4, CBFB, CCNT1, CCNT2, CDK12, CDK7, CDK9, CEBPB, CEBPD, COPS2, CTNNB1, DDX21, DEK, DRAP1, EDN1, ETS1, ETV1, FGF2, FOS, FOSB, GLIS3, GTF2A1, GTF2A2, GTF2H1, GTF2H3, IRF9, ISL1, JAK2, KLF4, MAF, MAPK14, MNT, NCAPG2, NFAT5, NFATC2, NFIX, NFKB1, NFX1, NFYA, NR4A1, PAXBP1, PIK3R1, POLR2B, POLR2C, POLR2J, PRDM4, PROX1, RPAP1, RREB1, SCAF1, SMAD1, SMARCC2, SNAPC3, SOX9, TAF1, TAF12, TAF5L, TAF7, TCEA1, TEAD2, TFAP2B, TFDP1, TP63, TRIM24, TRIP11, TRIP13, TSC22D1, XBP1	ACVR2A, ACVR2B, BMP4, BMP6, BMPR1A, BMPR2, CCN1, CEBPB, CEBPD, CTNNB1, FBXO5, FERMT2, FGF2, FZD1, HGF, IFITM1, IGF1, IL6, IL6R, IL6ST, ILK, JAG1, MEF2C, RUNX2, SMAD1, SMAD5, TP63, VEGFA, WWTR1, YAP1	CREB1, CSF1R, CTNNB1, FOS, FOSL2, FOXP1, GLO1, IREB2, JUNB, MAPK14, MITF, NF1, OSTM1, PIK3R1, SLC4A2, SNX10, TFRC, TMEM64, TNF	ACVR2A, ACVR2B, ADRB2, ATP2B1, BMP4, BMP6, BMPR1A, BMPR2, CCN1, GPM6B, ISG15, MEF2C, RXRB, SMAD3, TFAP2A
**hsa-miR-224-5p**	ATF4, BCL6, CCNT2, CTNNB1, ERCC2, ERCC3, GTF2H5, KLF13, NFAT5, NFIX, NR4A1, PARP1, POLR2F, PRDM4, SQSTM1, TAF1, TAF10, TAF5L, TFDP1, TSC22D1, XBP1	ATRAID, BMPR2, CTNNB1, FERMT2, IL6R, IL6ST, JUND, PPP3CA, SMAD5, SUCO, TENT5A, YAP1	CTNNB1, EPHA2, GLO1, IREB2	ATRAID, BMPR2, SLC20A2, TENT5A, VDR
**hsa-miR-30c-1-3p**	ABLIM3, ADRB2, AR, ARID4A, ARRB2, ASH1L, ATF4, ATRX, ATXN1L, BCL9, BCL9L, BMP2, CBFB, CCNK, CCNT1, CCNT2, CDK12, CHD7, COPS2, CTNNB1, DDX21, FGF2, FOSB, FOXP3, GTF2A1, GTF2H1, HOXA13, INHBA, IRF9, JAK2, KLF4, KPNA6, MAPK14, MED16, MNT, MYEF2, NFAT5, NFIC, NFIX, NFX1, NFYA, PARP1, PAX5, PIK3R1, PKNOX1, POLR2A, POLR2B, POLR2E, PRDM4, RB1, RREB1, SCAF1, SMAD1, SMAD4, SMARCC2, SOX9, SPDEF, SQSTM1, SRF, TAF1, TAF10, TAF5L, TBP, TRIP13, TSC22D1, ZNF768	ACVR2B, ATRAID, BMP2, BMPR1B, BMPR2, CLIC1, CTNNB1, DDR2, FERMT2, FGF2, GLI3, IL6R, IL6ST, ILK, JAG1, JUND, NPNT, PPP3CA, RUNX2, SMAD1, SMAD5, SUCO, TENT5A, WWTR1, YAP1	BMP2, CSF1, CTNNB1, EPHA2, FOSL2, FOXP1, GLO1, IREB2, JUNB, MAPK14, MITF, NF1, PIK3R1, SBNO2, SLC4A2, TFRC, TNF, TOB2	ACVR2B, ADRB2, ANO6, ATP2B1, ATRAID, BMP2, BMPR1B, BMPR2, RXRA, RXRB, SLC20A2, SMAD3, TENT5A, VDR
**hsa-miR-30c-5p**	ADRB2, AR, ARID4A, ARID4B, ARRB2, ASH1L, ATRX, ATXN1L, BCL11B, BCL9, CCNK, CCNT2, CDK12, CHD7, CTNNB1, DDX21, ETS1, GMEB2, GTF2A1, GTF2E2, GTF2H1, KLF13, KLF4, KPNA6, LHX1, MNT, MYEF2, NCAPG2, NFAT5, NFIC, NFKB1, NFX1, NOTCH1, PAXBP1, PIK3R1, PKNOX1, POLR2B, POLR2D, SLC40A1, SMAD4, SMARCC2, SRF, TBPL1, TFDP1, THRA, XBP1	ACVR1, BMPR2, CTHRC1, CTNNB1, FZD1, JAG1, MEF2C, PPP3CA, RUNX2, SMAD5, SUCO, VEGFA, YAP1	CTNNB1, EPHA2, FOSL2, FOXP1, IREB2, JUNB, NF1, PIK3R1, SLC4A2, SNX10, TFRC, TMEM64, TOB2	ACVR1, ADRB2, ATP2B1, BMPR2, MEF2C, SLC20A2
**hsa-miR-374a-3p**	AR, ATXN1L, CBFB, CDK12, CTNNB1, DDX21, ETS1, NFAT5, NFIC, NFIX, NFKB1, NFYA, PARP1, PIK3R1, SCAF1	BMPR2, CTNNB1, IL6, SMAD5	CTNNB1, GLO1, JUNB, PIK3R1, SBNO2, SLC4A2, TMEM64	ATP2B1, BMPR2
**hsa-miR-450a-5p**	ARID4A, ASH1L, ATXN1L, BCL9, BCL9L, BMP2, CBFB, CCNT2, CDK7, CDK9, CEBPD, CTNNB1, DRAP1, ERCC2, ETV1, FGF2, FOS, FOSB, GATA2, GTF2A1, GTF2H2, ISL1, JAK2, MBD1, MLIP, NFAT5, NFATC4, NFIC, NFIX, NFKB1, NOTCH1, NR4A1, PIK3R1, POLR2A, PROX1, RBMX, RREB1, SMAD4, SNAPC3, SRF, TAF10, TAF12, TEAD2, TP63, TSC22D1, ZNF141	ACVR1, ATRAID, BMP2, BMPR1A, BMPR2, CCN1, CEBPD, CLIC1, CTNNB1, DDR2, FBN2, FERMT2, FGF2, HGF, JAG1, JUND, MEF2C, SFRP2, SUCO, TENT5A, TP63, VEGFA, WWTR1, YAP1, ZHX3	BMP2, CTNNB1, EPHA2, FOS, FOSL2, GPR183, JUNB, MITF, NF1, PIK3R1, TFRC, TMEM64	ACVR1, ATRAID, BMP2, BMPR1A, BMPR2, CCN1, FBN2, ISG15, MEF2C, PTN, RXRA, SLC20A2, SMAD3, TENT5A, TFAP2A

## Data Availability

The original contributions presented in this study are included in the article/Supplementary Material. Further inquiries can be directed to the corresponding author.

## Supplementary Materials

The following supporting information can be downloaded at: https://www.mdpi.com/article/10.3390/ijms26031228/s1.

## Abbreviations

The following abbreviations are used in this manuscript:

OTM	orthodontic tooth movement
EVs	extracellular vesicles
GO	gene ontology
NTA TEM	nanoparticle tracking analysis transmission electron microscopy
