# Supplementary material for: Identification of Salivary Exosome-Derived miRNAs as Potential Biomarkers of Bone Remodeling During Orthodontic Tooth Movement
Source: Int J Mol Sci. 2025 Jan 30;26(3):1228. doi: 10.3390/ijms26031228 (PMC11818790; doi:10.3390/ijms26031228)
Supplement: Supplementary file 1 [file ijms-26-01228-s001.zip › Supplementary materials/Table S1/Tables API, SBI, PSI/Sample _6/Ergebnisse_PSI.pdf]

Ergebnisse Parodontaler Screening-Index (PSI)

Probe: 6

[redacted] [redacted]

Wir haben bei Ihnen den Parodontalen Screening-Index (PSI) erhoben. Der PSI bietet einen orientierenden Überblick über das mögliche Vorliegen und die Schwere einer parodontalen Erkrankung sowie den möglichen Behandlungsbedarf. Die bei Ihnen in der Untersuchung festgestellten Werte können Sie der nachfolgenden Tabelle entnehmen.

Einteilung von Ober- und Unterkiefer in je drei Sextanten (S 1 - S 6)

Oberkiefer

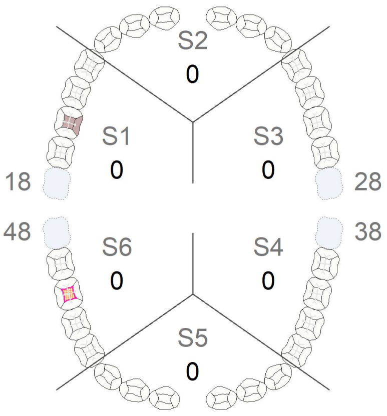

Unterkiefer

Höchster im Sextanten festgestellter Code

S 1	**0**
S 2	**0**
S 3	**0**
S 4	**0**
S 5	**0**
S 6	**0**

Erläuterung des PSI-Codes

0 Sondierungstiefe kleiner 3,5 mm, keine Blutung auf Sondierung, kein Zahnstein, keine überstehenden Füllungs-/Kronenränder

1 Sondierungstiefe kleiner 3,5 mm, Blutung auf Sondierung, kein Zahnstein, keine überstehenden Füllungs-/Kronenränder

2 Sondierungstiefe kleiner 3,5 mm, Zahnstein und/oder überstehende Füllungs-/Kronenränder

3 Sondierungstiefe 3,5 bis 5,5 mm

4 Sondierungstiefe größer 5,5 mm

* Auffälligkeiten wie z.B. Zahnfleischrückgang oder Zahnlockerung sind mit einem Stern gekennzeichnet

Aus den Screening-Ergebnissen ergeben sich folgende Diagnosen und Empfehlungen.

PSI-Code	Diagnose	Empfehlungen, möglicher Untersuchungs- und Behandlungsbedarf
0	Parodontal gesund	Keine Therapie notwendig, regelmäßige Kontrolluntersuchung
1	Zahnfleischentzündung (Gingivitis)	Verbesserung der Mundhygiene
2	Zahnfleischentzündung (Gingivitis), Zahnstein oder überstehende Füllungs- oder Kronenränder	Verbesserung der Mundhygiene, Zahnsteinentfernung oder Glättung überstehender Füllungs- und Kronenränder
3<br>4	Verdacht auf Parodontitis	Verbesserung der Mundhygiene, parodontale Befunderhebung einschließlich der Anfertigung von Röntgenbildern als Basis der Diagnosestellung und der weiteren Therapieplanung

Wir haben Sie über das Untersuchungsergebnis, den möglichen Behandlungsbedarf sowie - bei Messergebnis Code 3 oder 4 - über die Notwendigkeit, einen klinischen und einen röntgenologischen Befund zu erheben sowie die Diagnose zu stellen, informiert.

Sonstige/weitere Empfehlung:

72451
Dr. med. dent.
Nataly Iserhardt
Kieferorthopädin
Erkelenzer Straße 131
50181 Bedburg
Tel.: 0 22 72 / 24 34

Bedburg, 06.03.2023
