# Supplementary figures and images for: Identification of Salivary Exosome-Derived miRNAs as Potential Biomarkers of Bone Remodeling During Orthodontic Tooth Movement
Source: Int J Mol Sci. 2025 Jan 30;26(3):1228. doi: 10.3390/ijms26031228 (PMC11818790; doi:10.3390/ijms26031228)

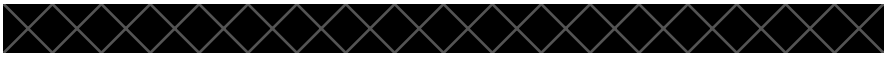

Probe: 10

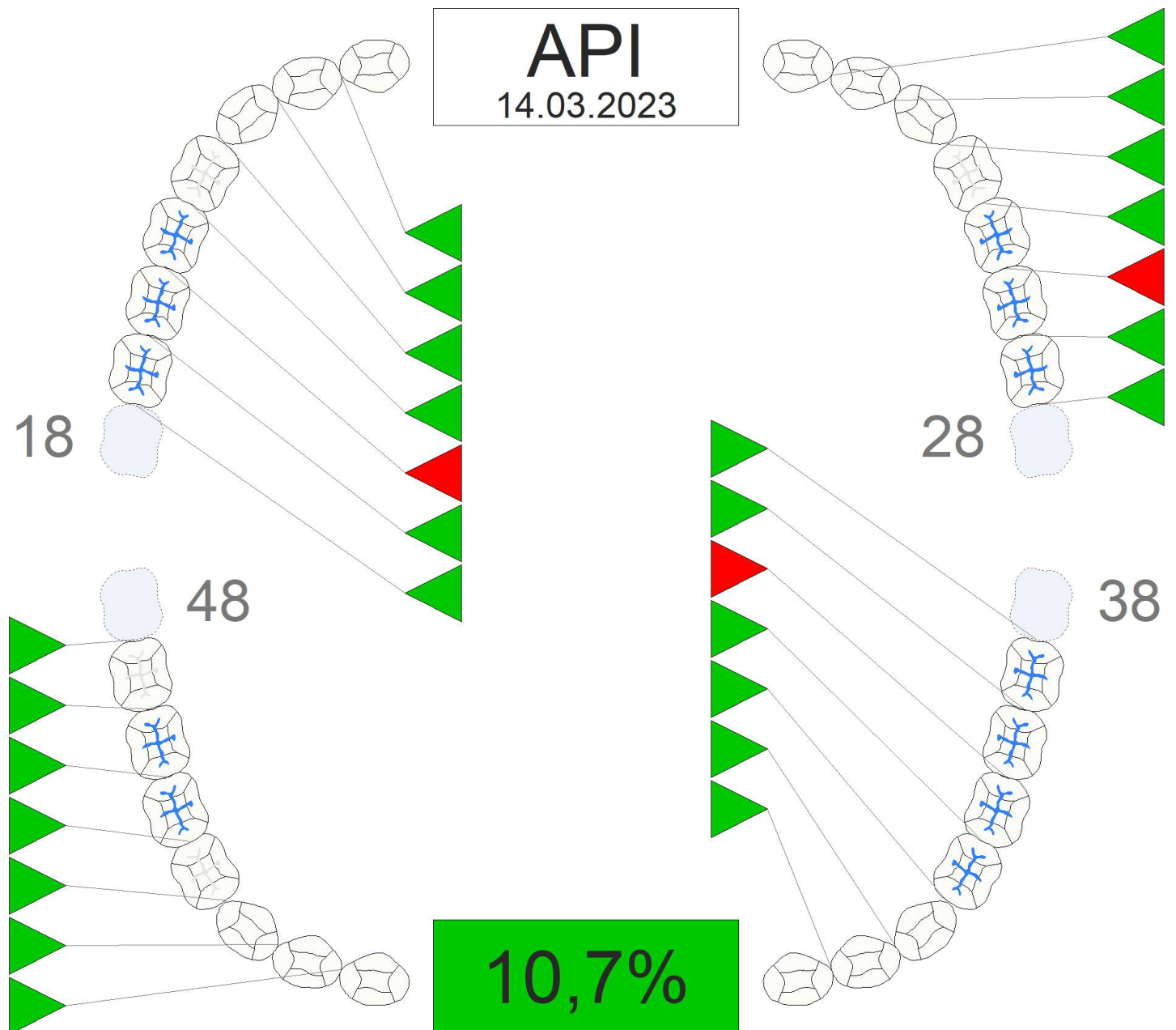

Supplement: Supplementary file 1 [file ijms-26-01228-s001.zip › Supplementary materials/Table S1/Tables API, SBI, PSI/Sample _10/API.pdf]

| Age Group | Percentage |
|-----------|------------|
| 18-24     | 25%        |
| 25-34     | 35%        |
| 35-44     | 15%        |
| 45-54     | 10%        |
| 55-64     | 8%         |
| 65-74     | 5%         |
| 75-84     | 3%         |
| 85+       | 1%         |

## Probe: 10

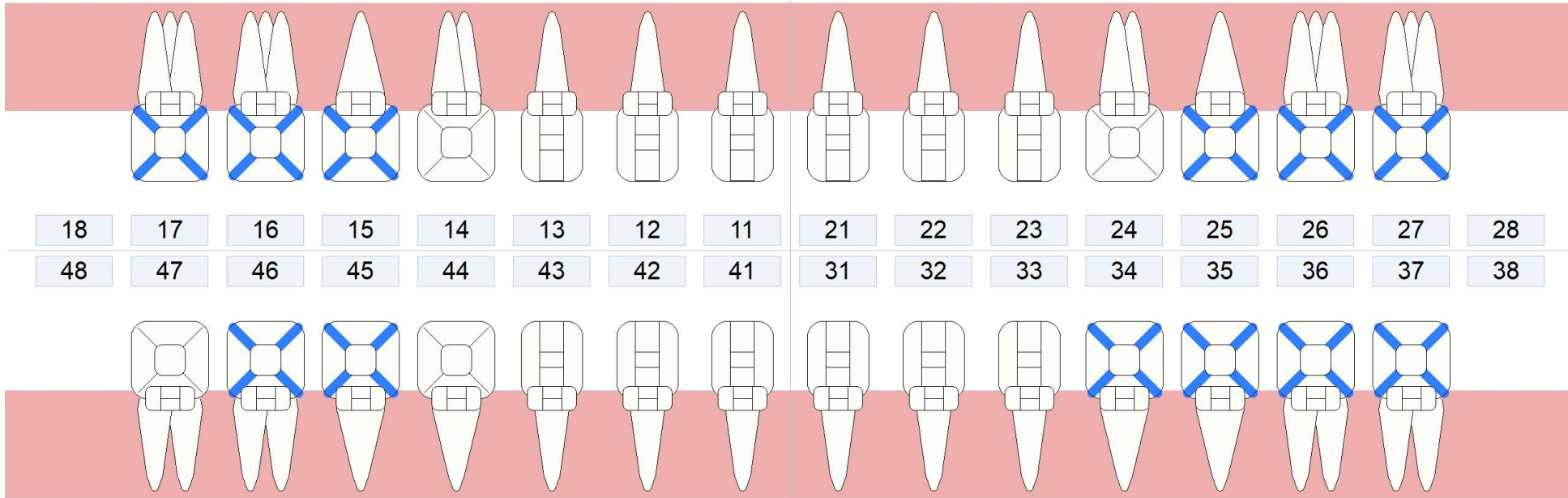

Supplement: Supplementary file 1 [file ijms-26-01228-s001.zip › Supplementary materials/Table S1/Tables API, SBI, PSI/Sample _10/Befund.pdf]

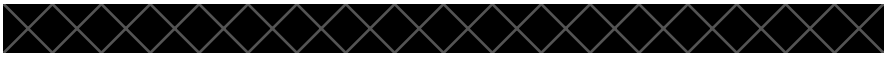

Probe: 10

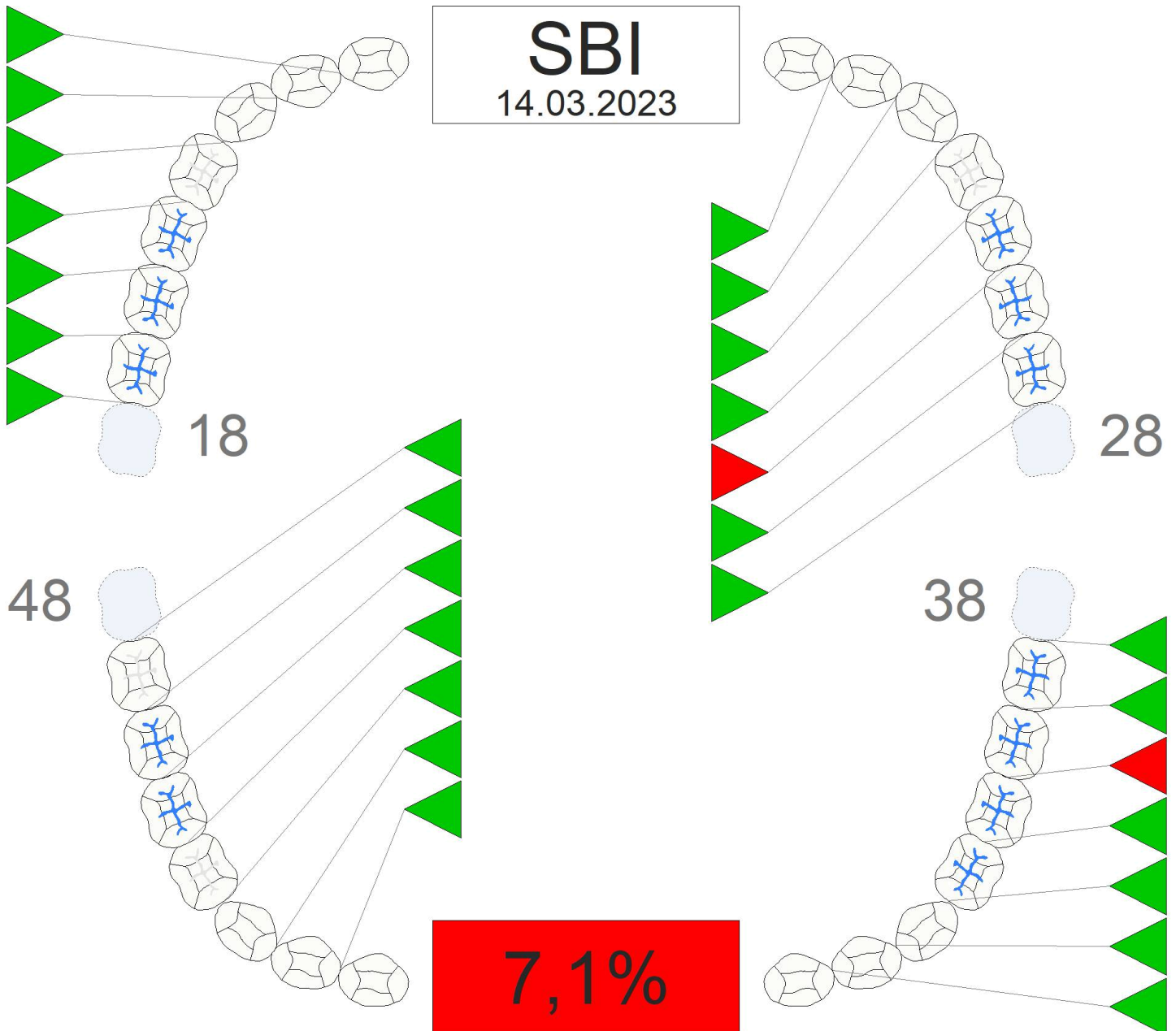

Supplement: Supplementary file 1 [file ijms-26-01228-s001.zip › Supplementary materials/Table S1/Tables API, SBI, PSI/Sample _10/SBI.pdf]

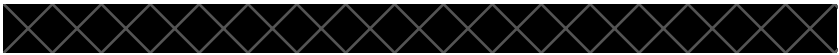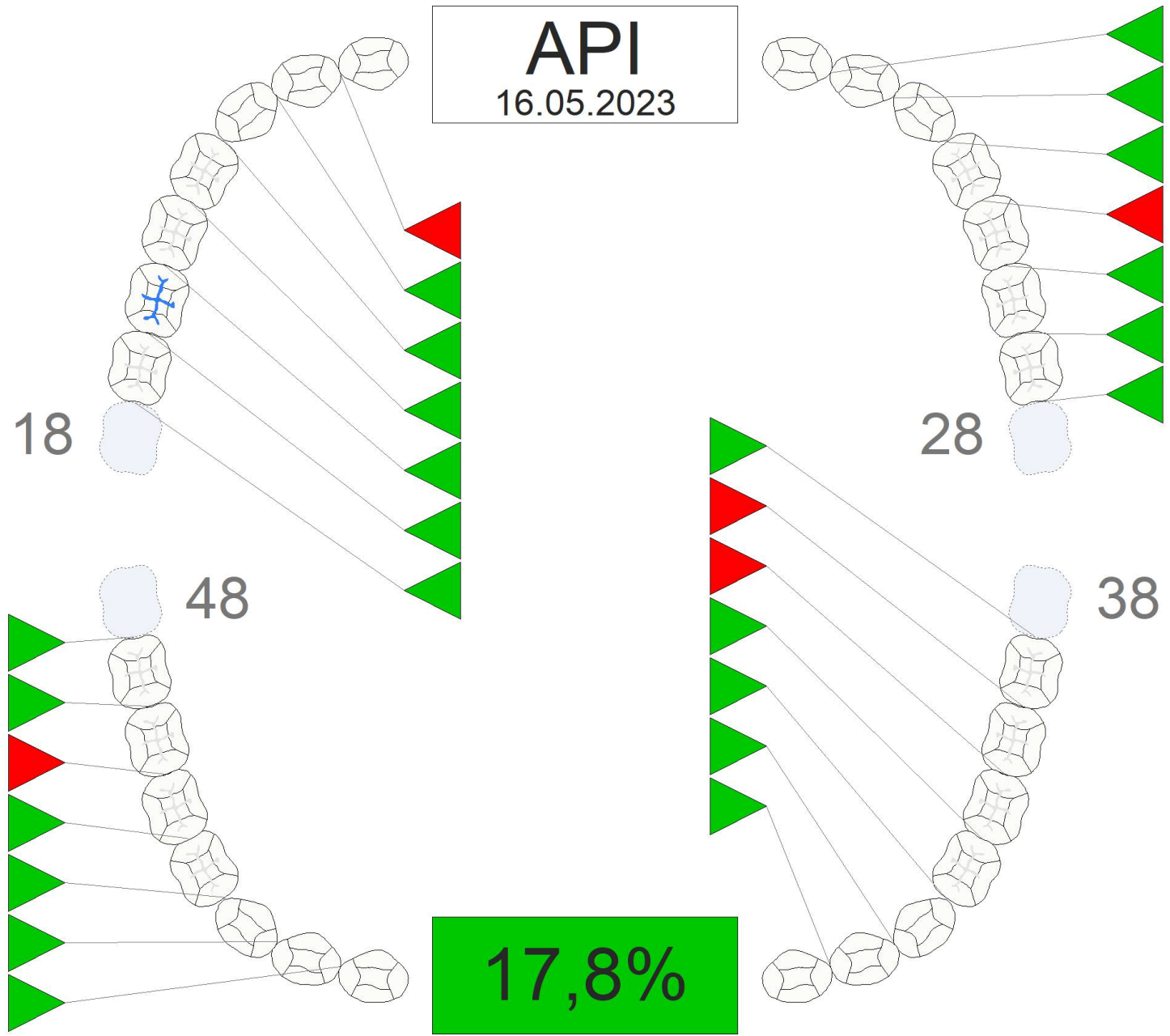

Supplement: Supplementary file 1 [file ijms-26-01228-s001.zip › Supplementary materials/Table S1/Tables API, SBI, PSI/Sample _11/API.pdf]

Zahnbefund

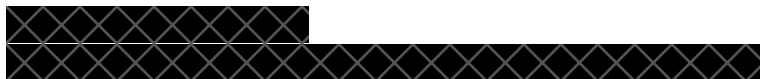

**Probe: 11**  
**16.05.23**

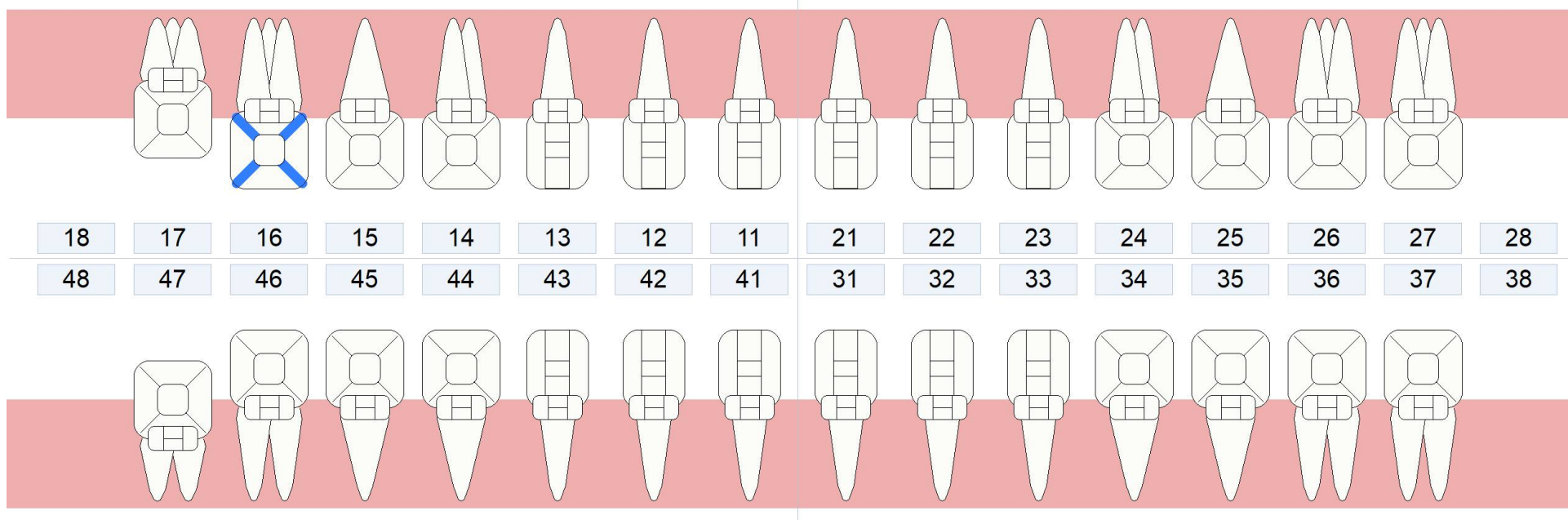

Supplement: Supplementary file 1 [file ijms-26-01228-s001.zip › Supplementary materials/Table S1/Tables API, SBI, PSI/Sample _11/Befund.pdf]

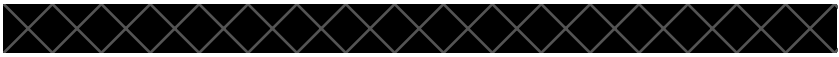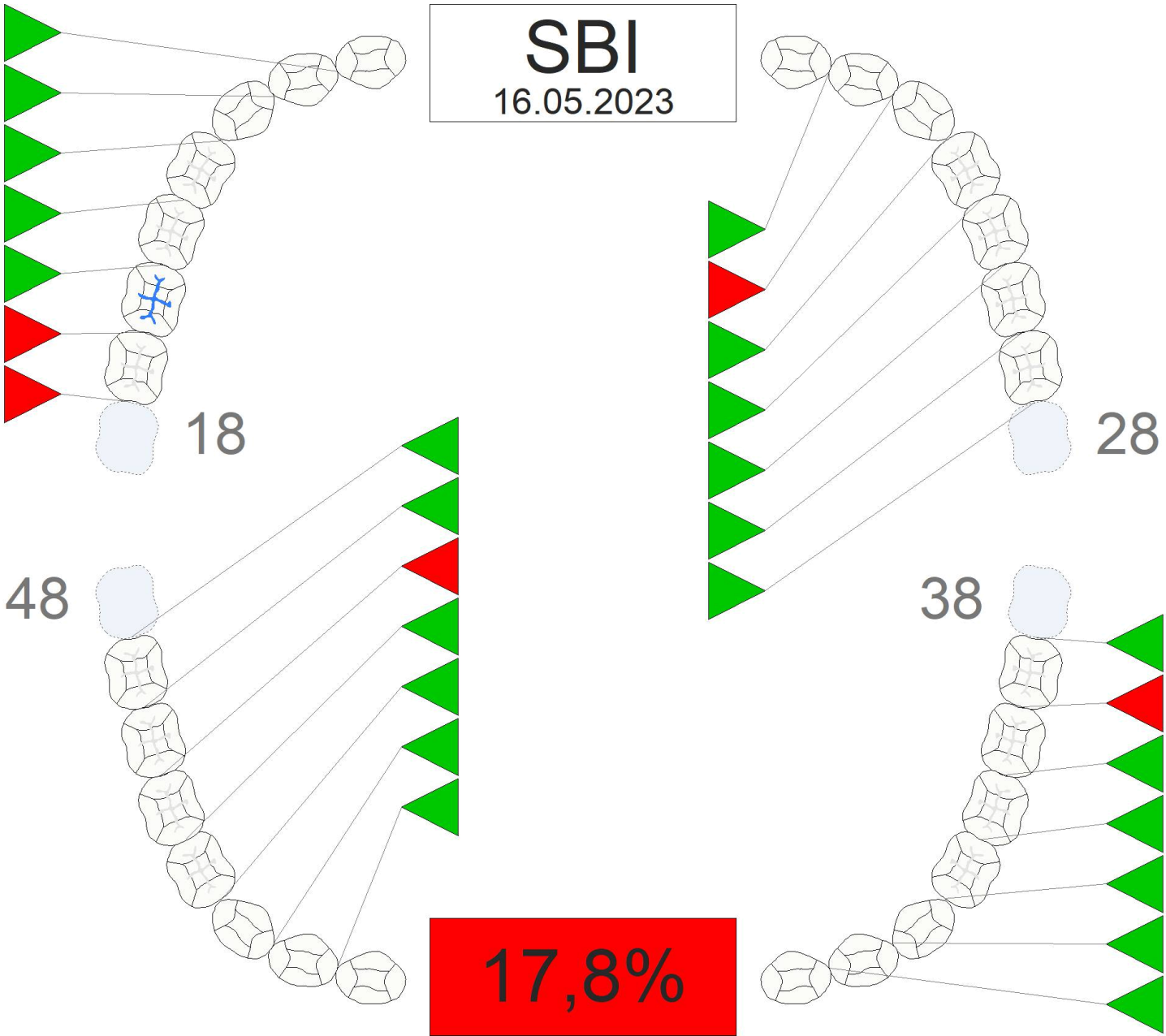

Supplement: Supplementary file 1 [file ijms-26-01228-s001.zip › Supplementary materials/Table S1/Tables API, SBI, PSI/Sample _11/SBI.pdf]

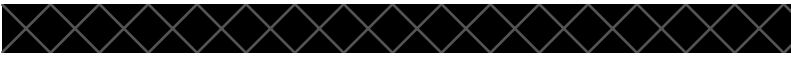

Probe: 12

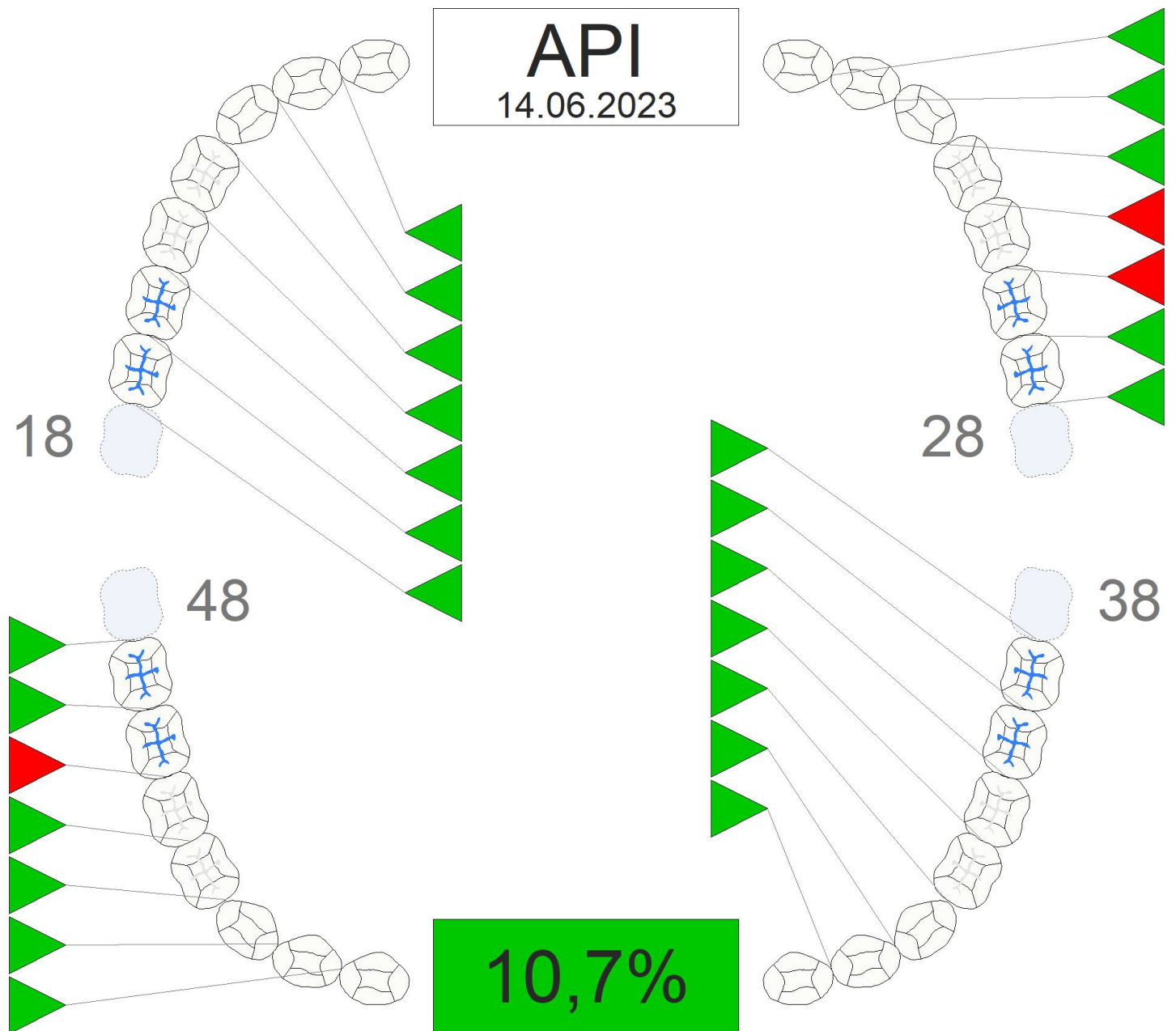

Supplement: Supplementary file 1 [file ijms-26-01228-s001.zip › Supplementary materials/Table S1/Tables API, SBI, PSI/Sample _12/API.pdf]

Zahnbefund

Probe: 12

14.06.23

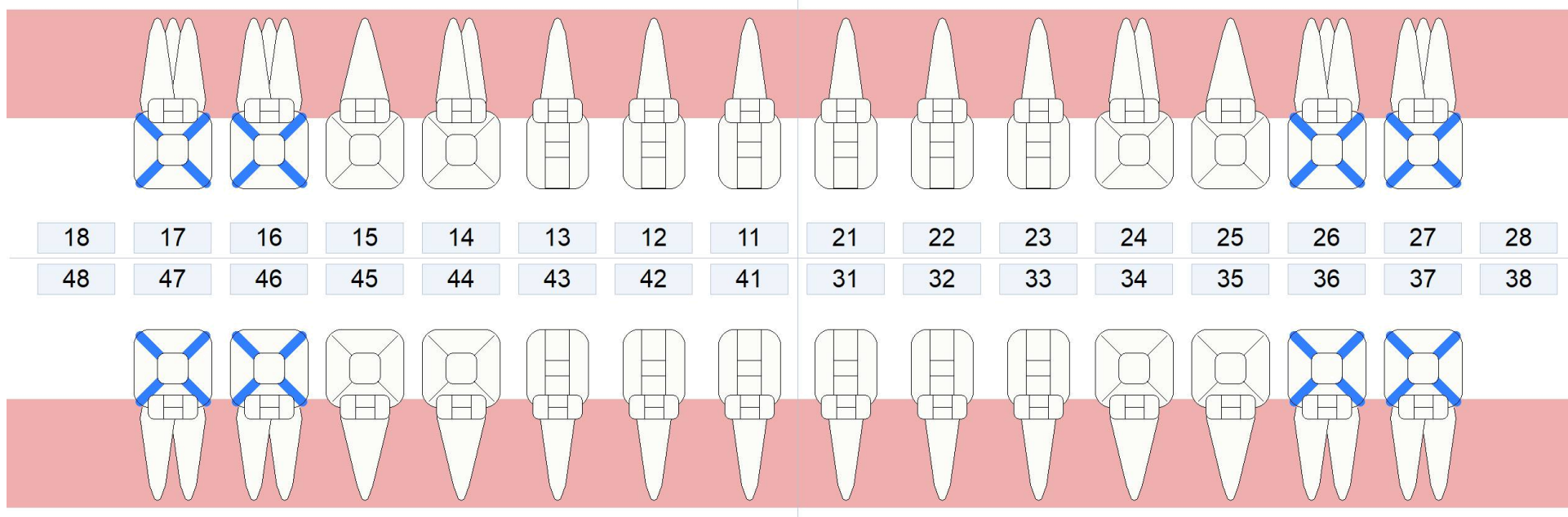

Supplement: Supplementary file 1 [file ijms-26-01228-s001.zip › Supplementary materials/Table S1/Tables API, SBI, PSI/Sample _12/Befund.pdf]

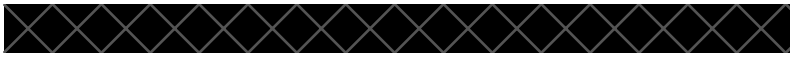

Probe: 12

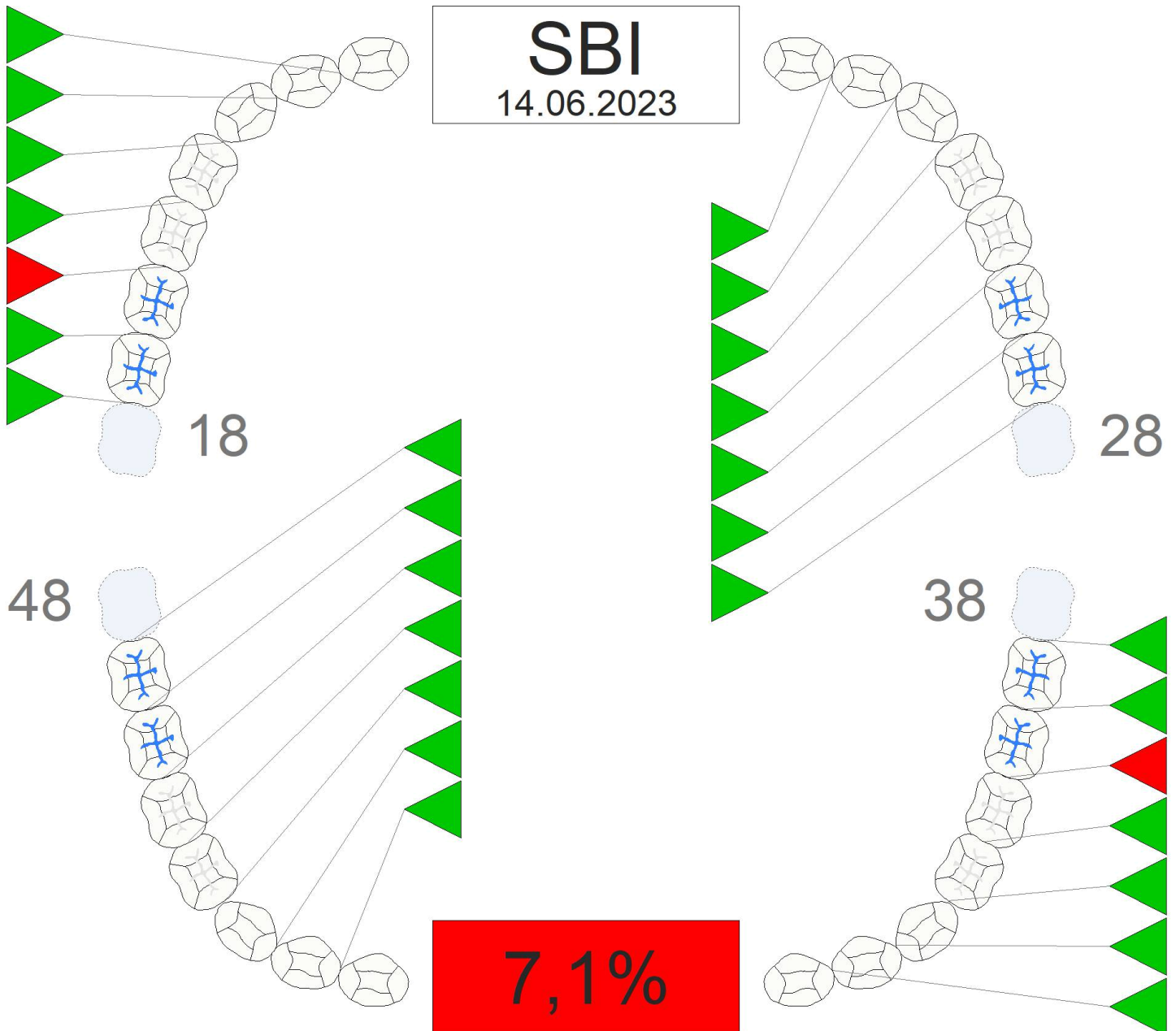

Supplement: Supplementary file 1 [file ijms-26-01228-s001.zip › Supplementary materials/Table S1/Tables API, SBI, PSI/Sample _12/SBI.pdf]

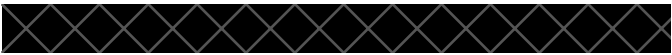

Probe: 13

API  
19.06.2023

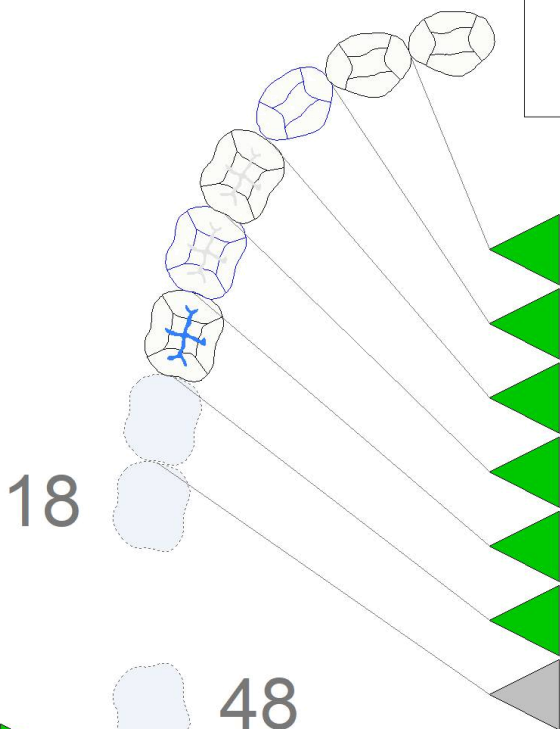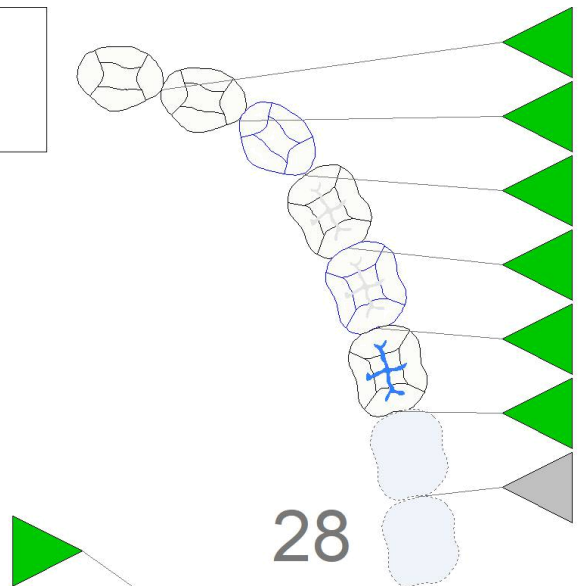

0%

Supplement: Supplementary file 1 [file ijms-26-01228-s001.zip › Supplementary materials/Table S1/Tables API, SBI, PSI/Sample _13/API.pdf]

Zahnbefund

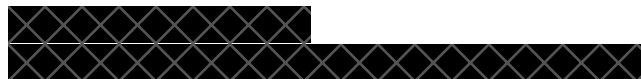

Probe: 13

08.03.23

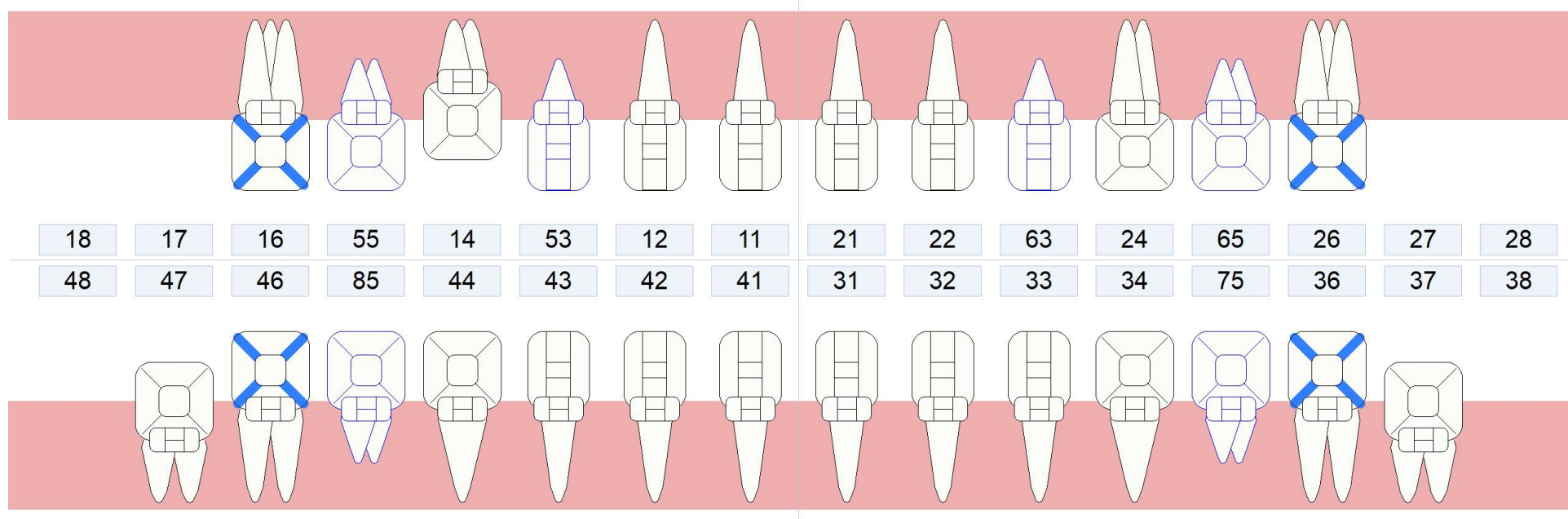

Supplement: Supplementary file 1 [file ijms-26-01228-s001.zip › Supplementary materials/Table S1/Tables API, SBI, PSI/Sample _13/Befund.pdf]

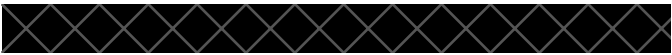

Probe: 13

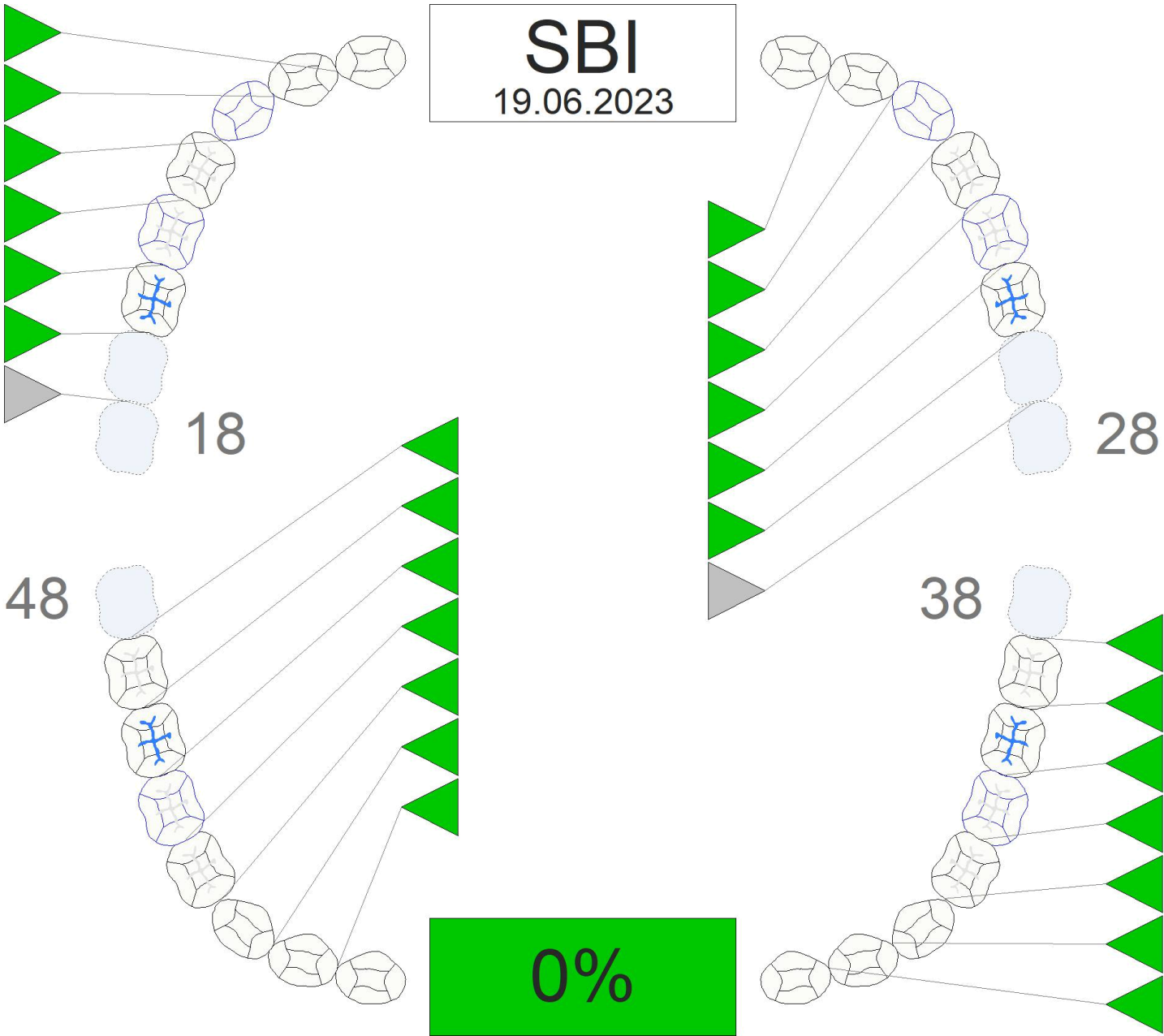

Supplement: Supplementary file 1 [file ijms-26-01228-s001.zip › Supplementary materials/Table S1/Tables API, SBI, PSI/Sample _13/SBI.pdf]

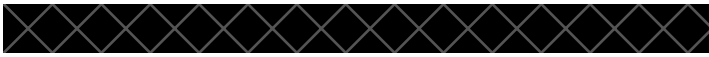

Probe: 14

API  
21.06.2023

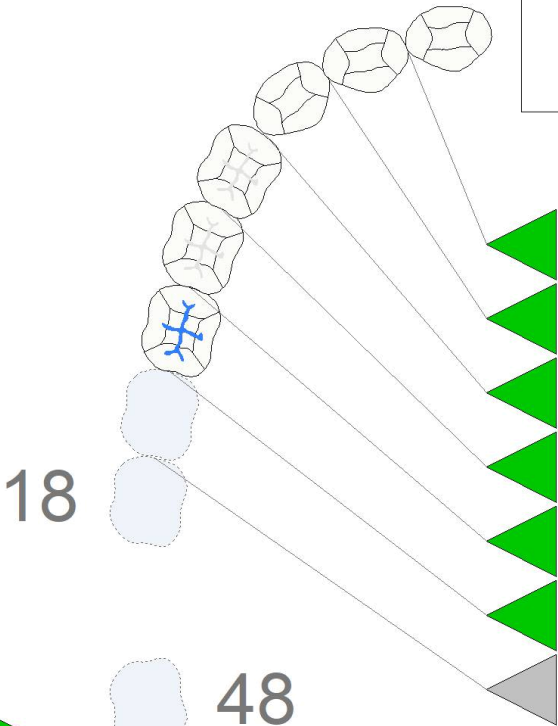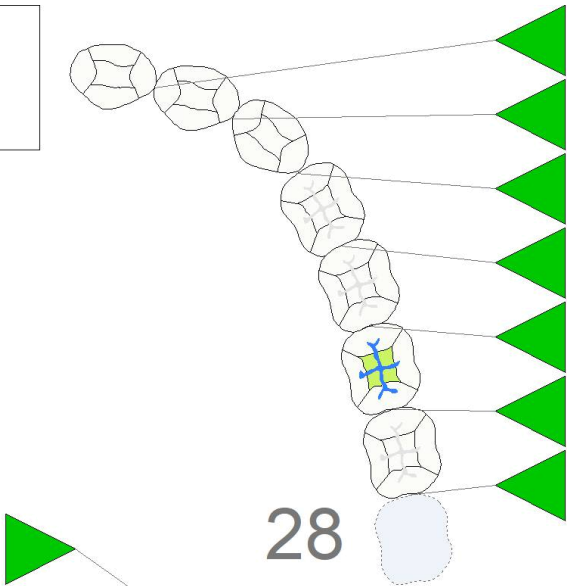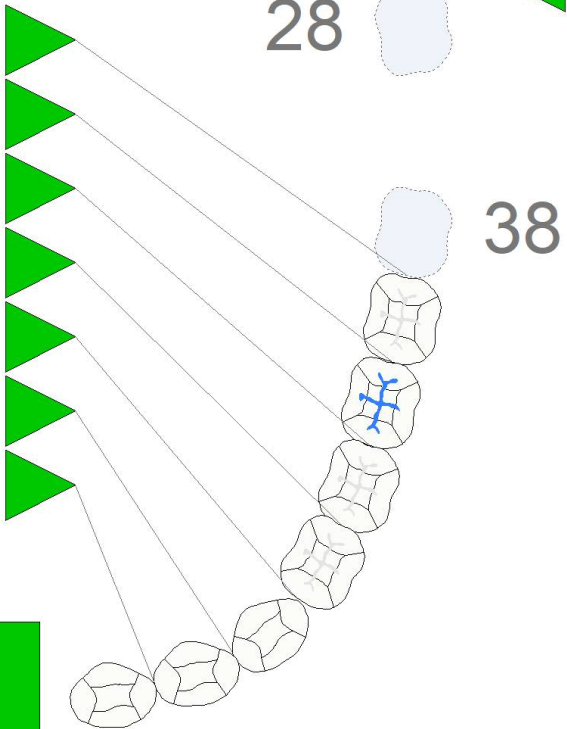

0%

Supplement: Supplementary file 1 [file ijms-26-01228-s001.zip › Supplementary materials/Table S1/Tables API, SBI, PSI/Sample _14/API.pdf]

Zahnbefund

Probe: 14

27.04.23

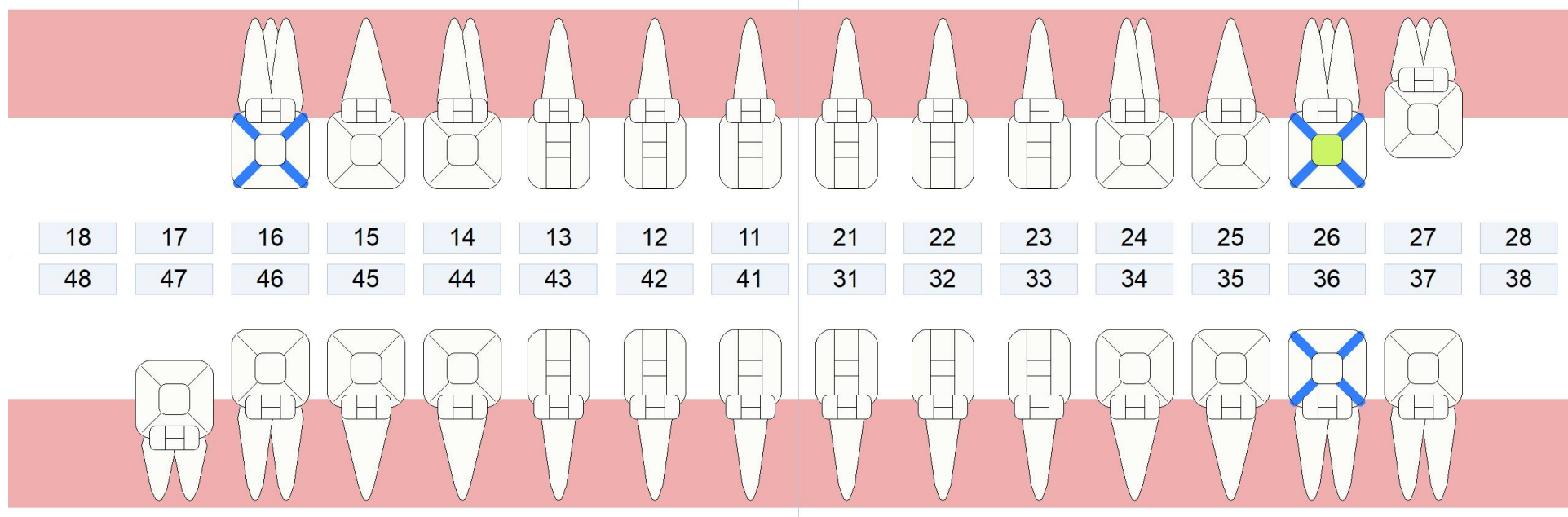

Supplement: Supplementary file 1 [file ijms-26-01228-s001.zip › Supplementary materials/Table S1/Tables API, SBI, PSI/Sample _14/Befund.pdf]

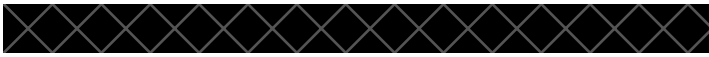

Probe: 14

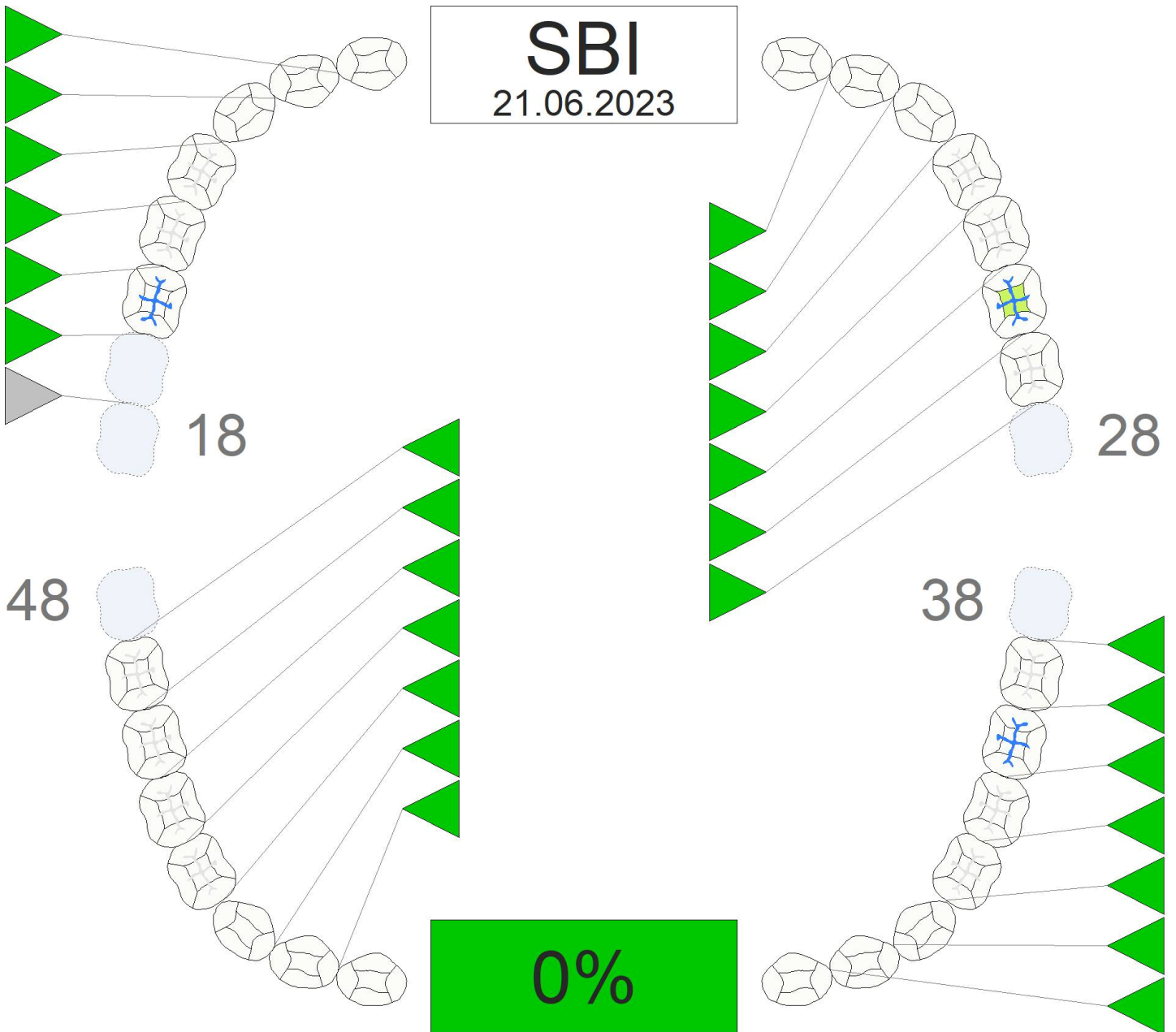

Supplement: Supplementary file 1 [file ijms-26-01228-s001.zip › Supplementary materials/Table S1/Tables API, SBI, PSI/Sample _14/SBI.pdf]

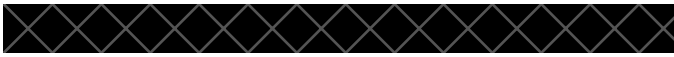

Probe: 15

API  
30.08.2023

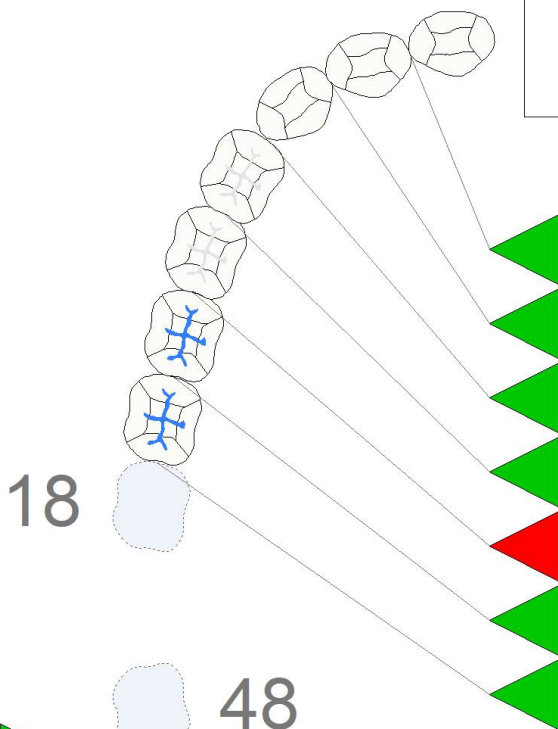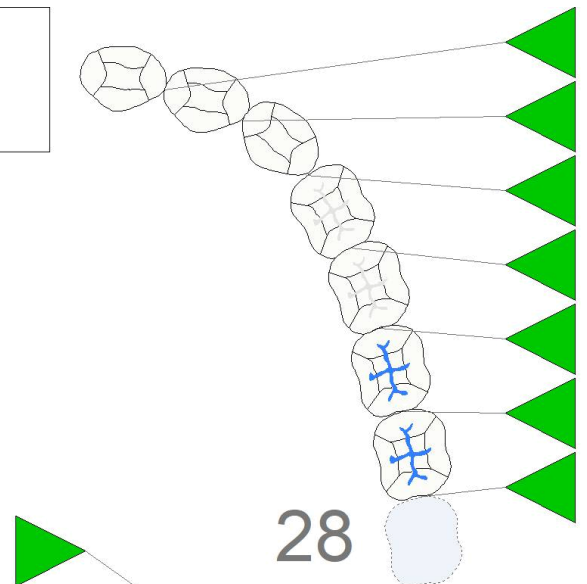

10,7%

Supplement: Supplementary file 1 [file ijms-26-01228-s001.zip › Supplementary materials/Table S1/Tables API, SBI, PSI/Sample _15/API.pdf]

# Zahnbefund

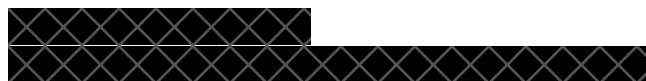

Probe: 15

30.08.23

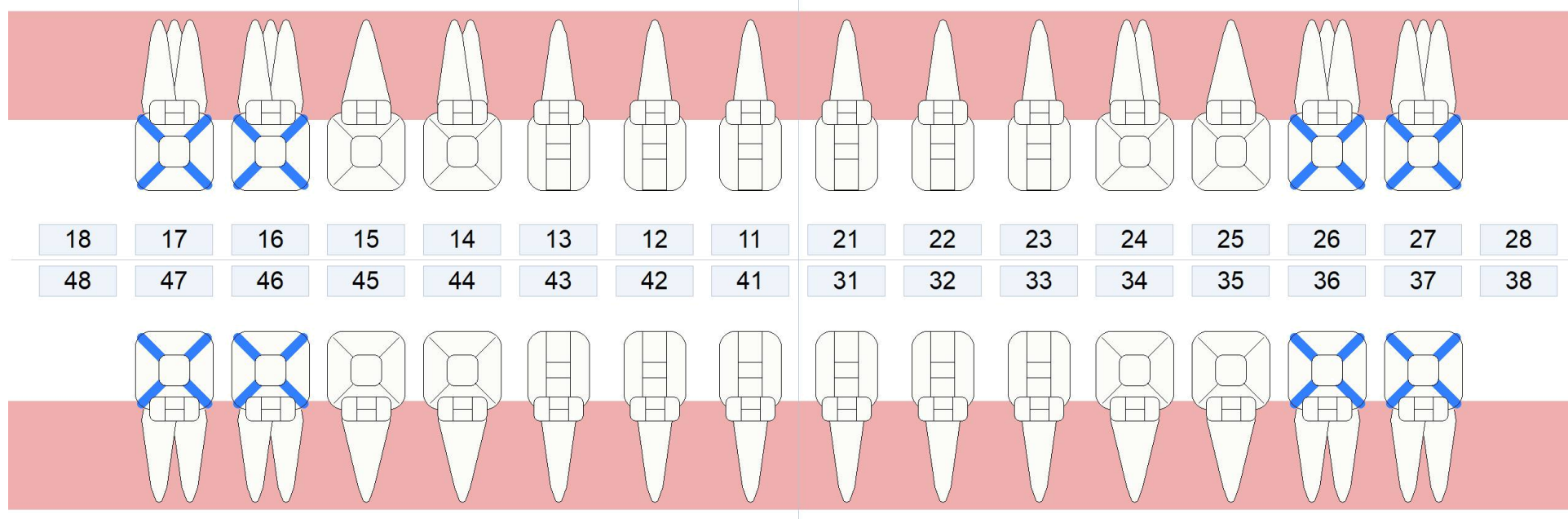

Supplement: Supplementary file 1 [file ijms-26-01228-s001.zip › Supplementary materials/Table S1/Tables API, SBI, PSI/Sample _15/Befund.pdf]

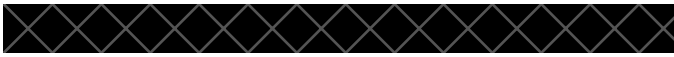

Probe: 15

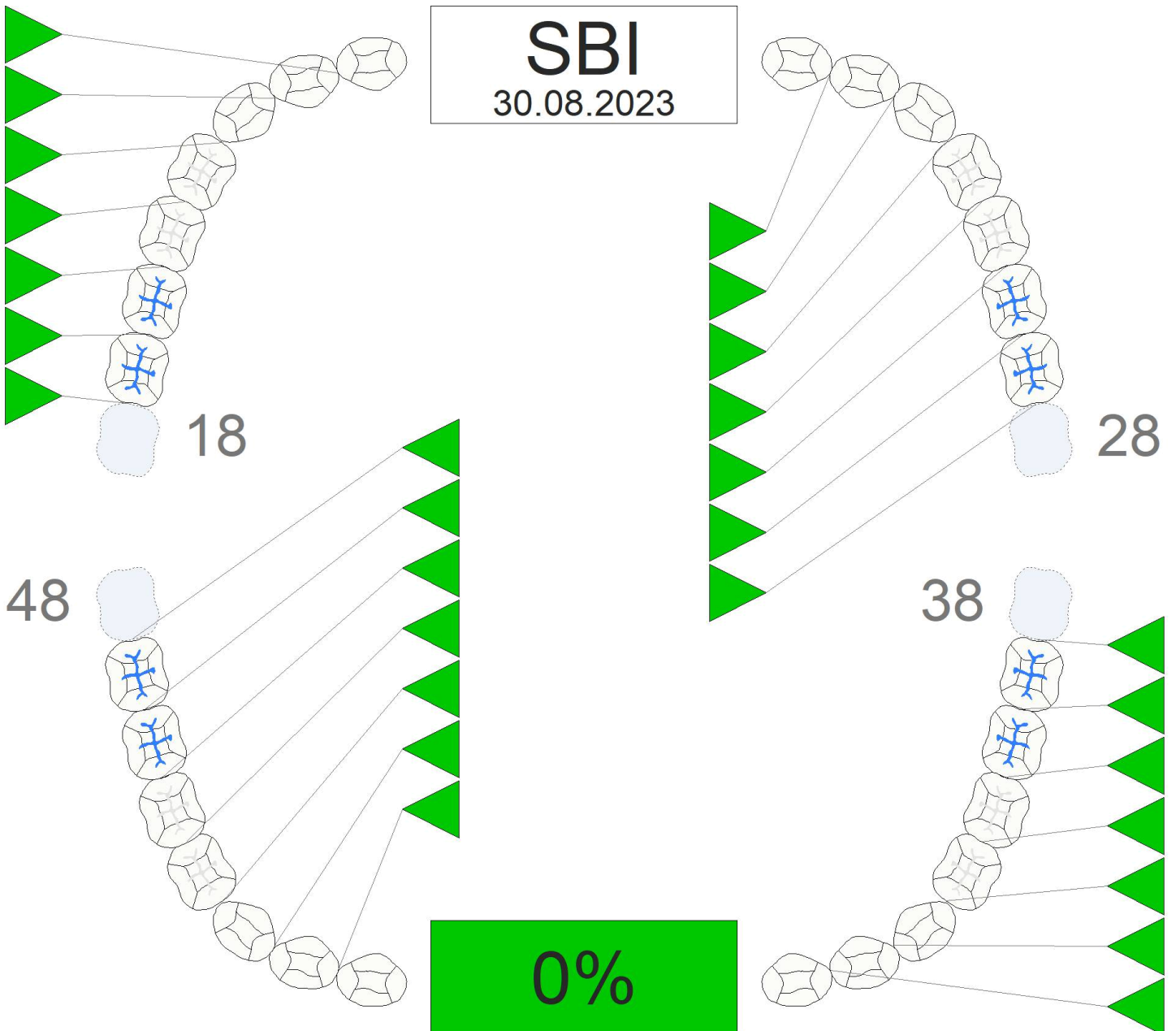

Supplement: Supplementary file 1 [file ijms-26-01228-s001.zip › Supplementary materials/Table S1/Tables API, SBI, PSI/Sample _15/SBI.pdf]

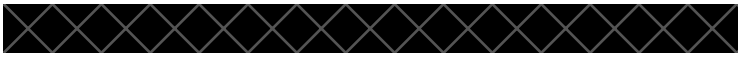

Probe: 16

API  
25.08.2023

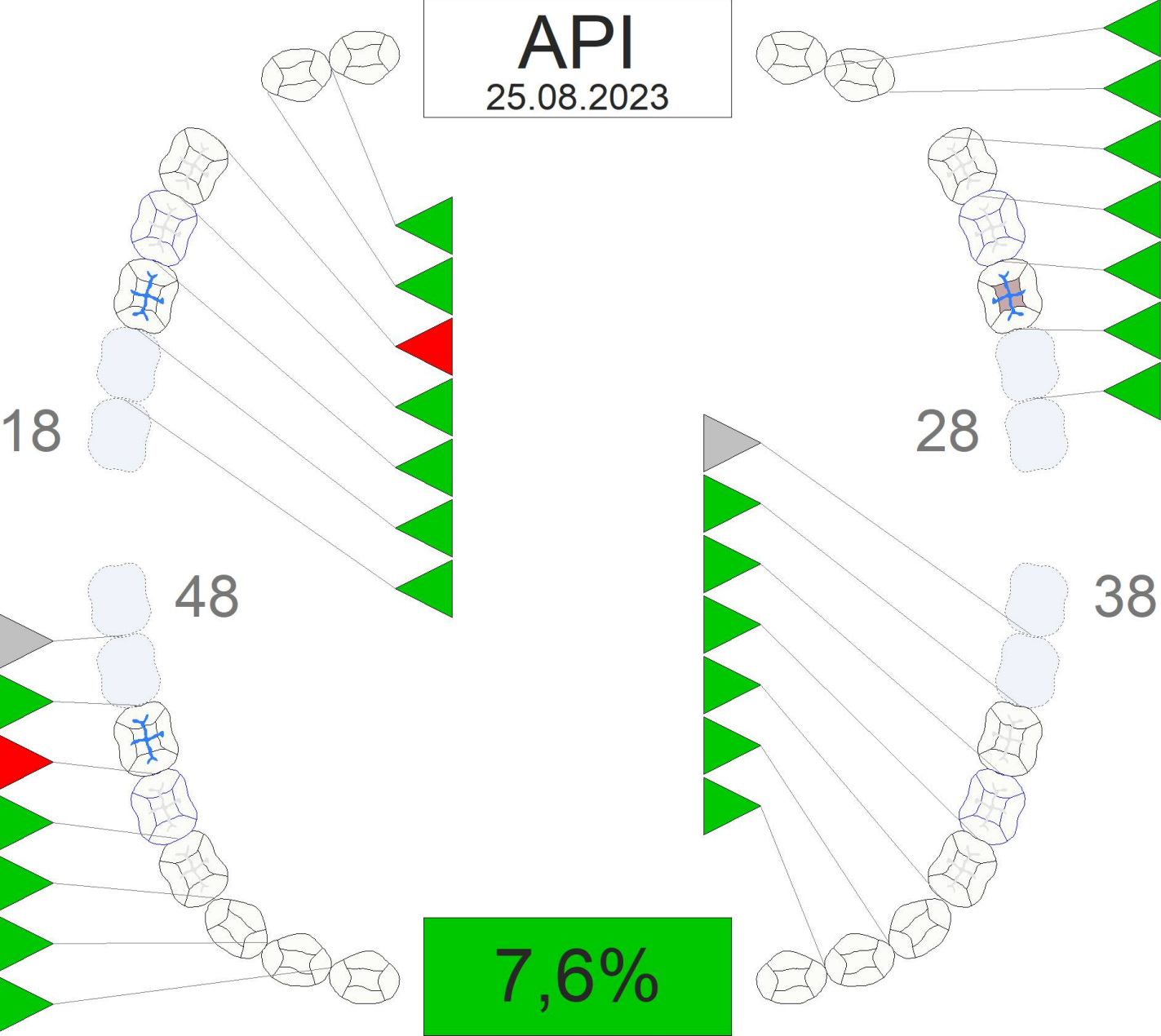

Supplement: Supplementary file 1 [file ijms-26-01228-s001.zip › Supplementary materials/Table S1/Tables API, SBI, PSI/Sample _16/API.pdf]

Zahnbefund

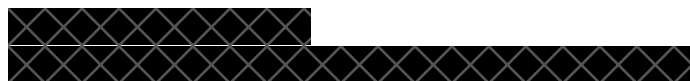

Probe: 16

04.07.23

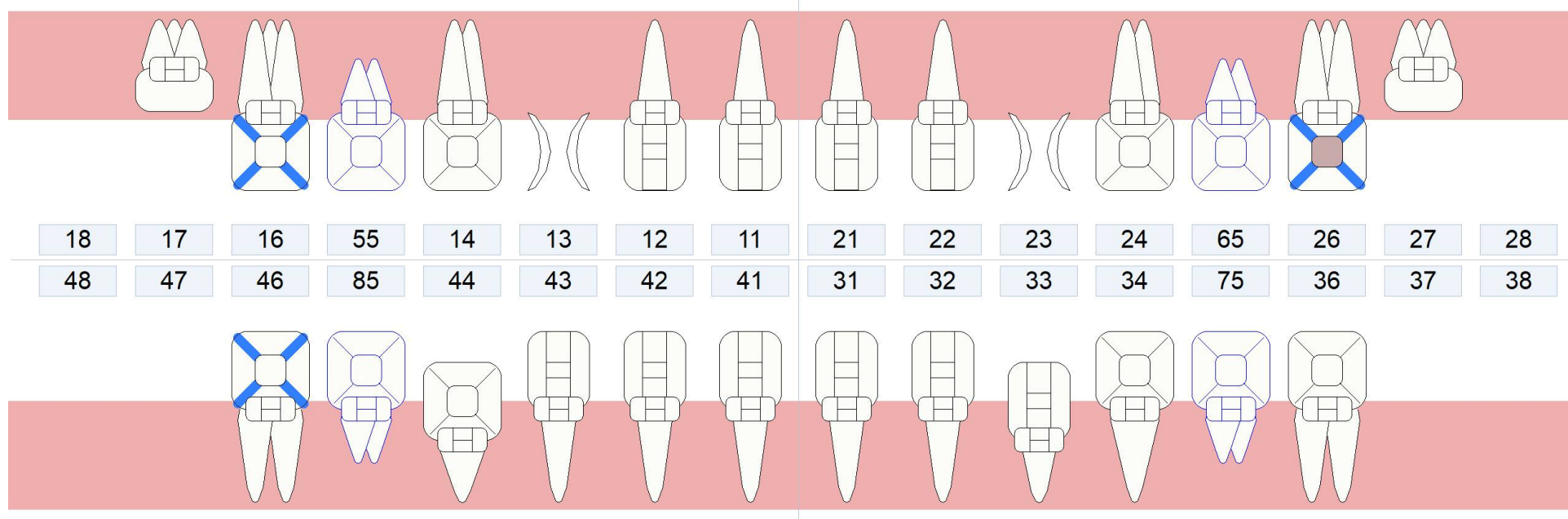

Supplement: Supplementary file 1 [file ijms-26-01228-s001.zip › Supplementary materials/Table S1/Tables API, SBI, PSI/Sample _16/Befund.pdf]

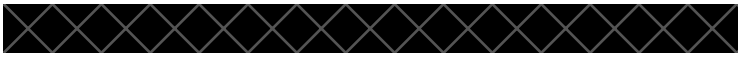

Probe: 16

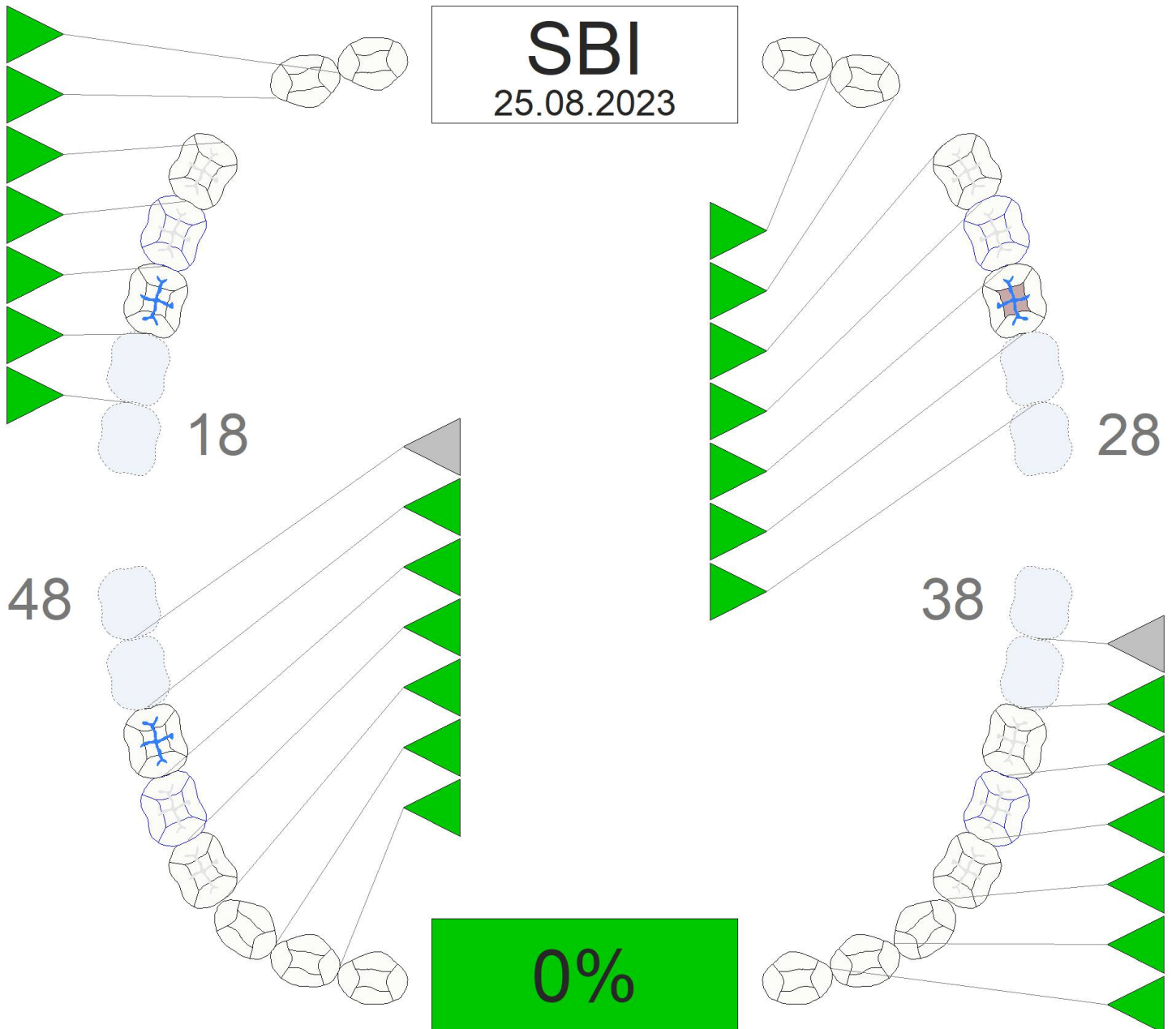

Supplement: Supplementary file 1 [file ijms-26-01228-s001.zip › Supplementary materials/Table S1/Tables API, SBI, PSI/Sample _16/SBI.pdf]

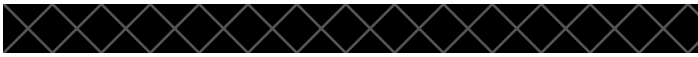

Probe: 1

API  
23.01.2023

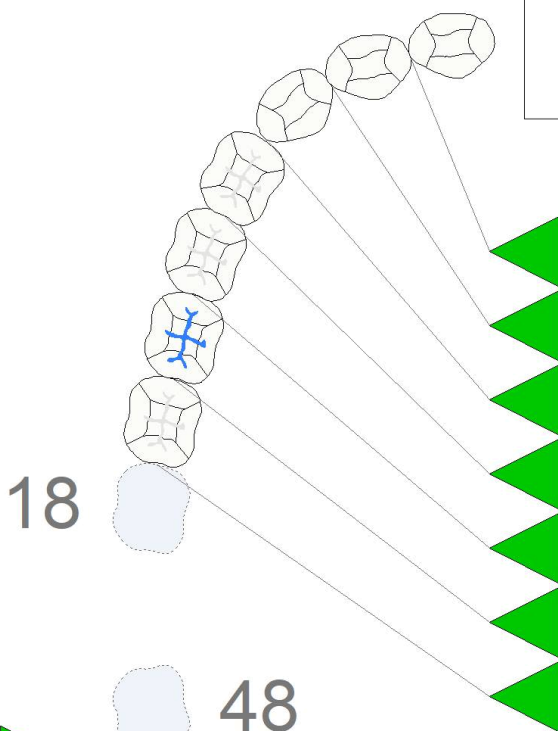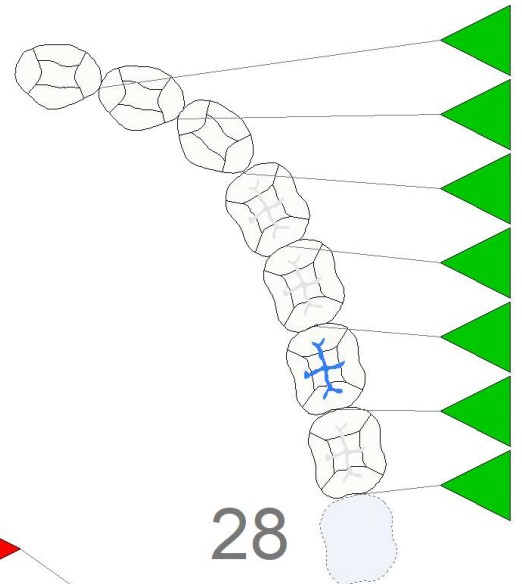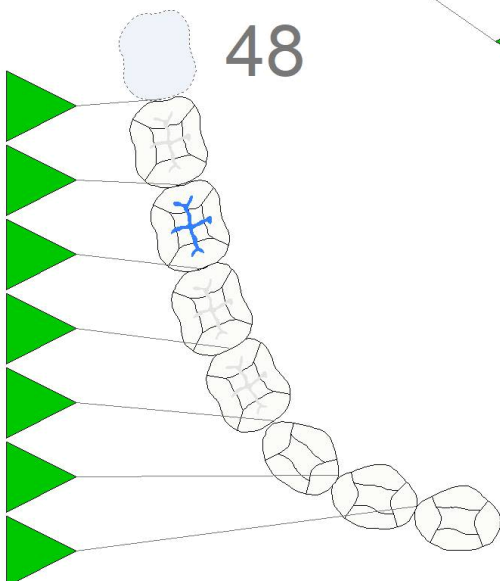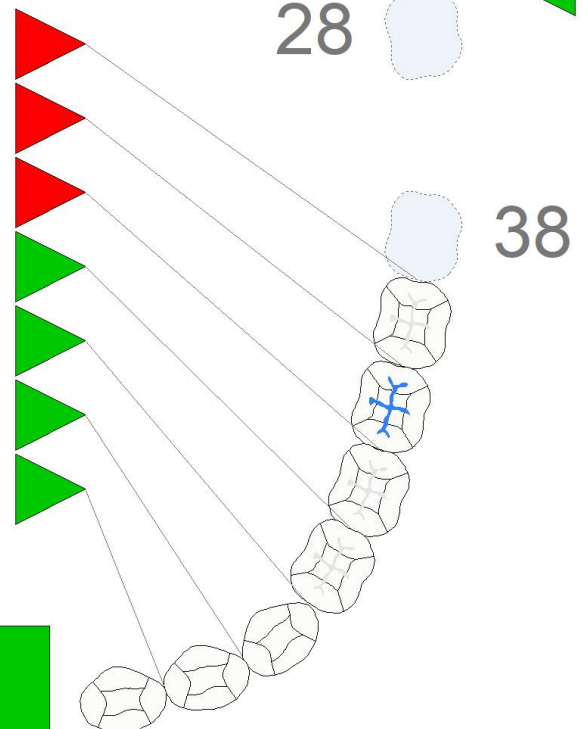

10,7%

Supplement: Supplementary file 1 [file ijms-26-01228-s001.zip › Supplementary materials/Table S1/Tables API, SBI, PSI/Sample _1/API.pdf]

|                                                                                    |                                                                                    |                                                                                    |                                                                                    |                                                                                    |                                                                                    |                                                                                      |                                                                                      |                                                                                      |                                                                                      |                                                                                      |                                                                                      |                                                                                      |                                                                                      |                                                                                      |                                                                                      |
|------------------------------------------------------------------------------------|------------------------------------------------------------------------------------|------------------------------------------------------------------------------------|------------------------------------------------------------------------------------|------------------------------------------------------------------------------------|------------------------------------------------------------------------------------|--------------------------------------------------------------------------------------|--------------------------------------------------------------------------------------|--------------------------------------------------------------------------------------|--------------------------------------------------------------------------------------|--------------------------------------------------------------------------------------|--------------------------------------------------------------------------------------|--------------------------------------------------------------------------------------|--------------------------------------------------------------------------------------|--------------------------------------------------------------------------------------|--------------------------------------------------------------------------------------|
| 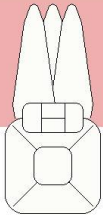  | 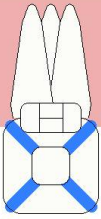  | 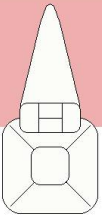  | 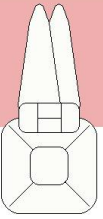  | 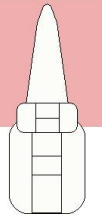  | 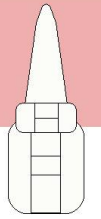  | 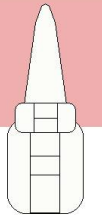  | 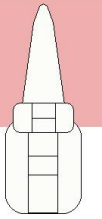  | 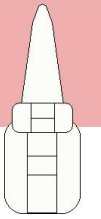  | 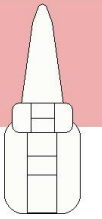  | 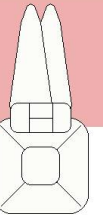  | 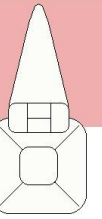  | 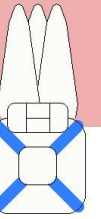  | 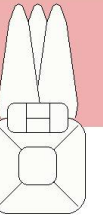  | 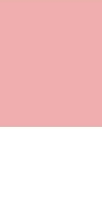  | 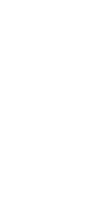  |
| 18                                                                                 | 17                                                                                 | 16                                                                                 | 15                                                                                 | 14                                                                                 | 13                                                                                 | 12                                                                                   | 11                                                                                   | 21                                                                                   | 22                                                                                   | 23                                                                                   | 24                                                                                   | 25                                                                                   | 26                                                                                   | 27                                                                                   | 28                                                                                   |
| 48                                                                                 | 47                                                                                 | 46                                                                                 | 45                                                                                 | 44                                                                                 | 43                                                                                 | 42                                                                                   | 41                                                                                   | 31                                                                                   | 32                                                                                   | 33                                                                                   | 34                                                                                   | 35                                                                                   | 36                                                                                   | 37                                                                                   | 38                                                                                   |
| 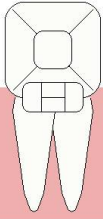 | 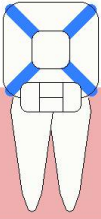 | 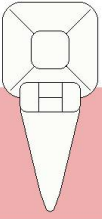 | 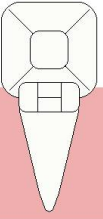 | 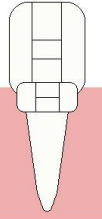 | 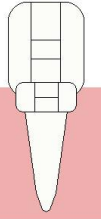 | 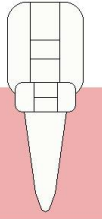 | 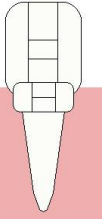 | 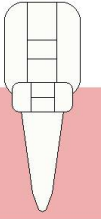 | 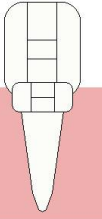 | 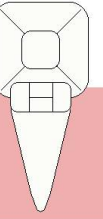 | 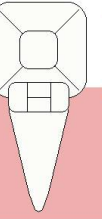 | 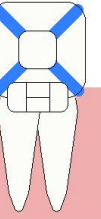 | 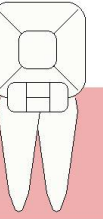 | 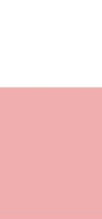 | 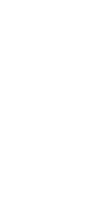 |

Supplement: Supplementary file 1 [file ijms-26-01228-s001.zip › Supplementary materials/Table S1/Tables API, SBI, PSI/Sample _1/Befund.pdf]

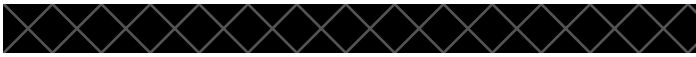

Probe: 1

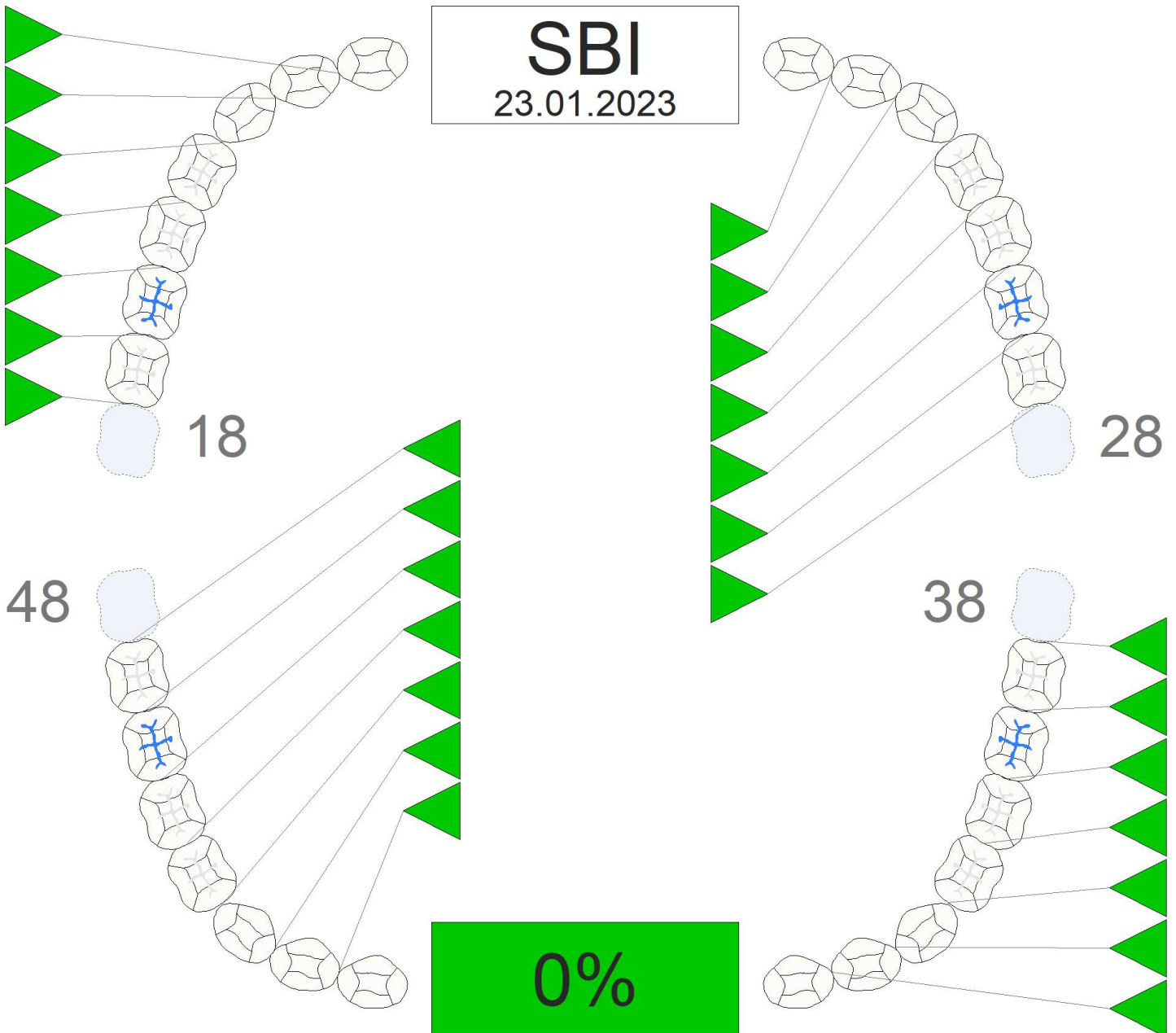

Supplement: Supplementary file 1 [file ijms-26-01228-s001.zip › Supplementary materials/Table S1/Tables API, SBI, PSI/Sample _1/SBI.pdf]

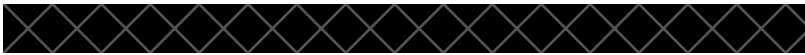

Probe: 2

API  
07.02.2023

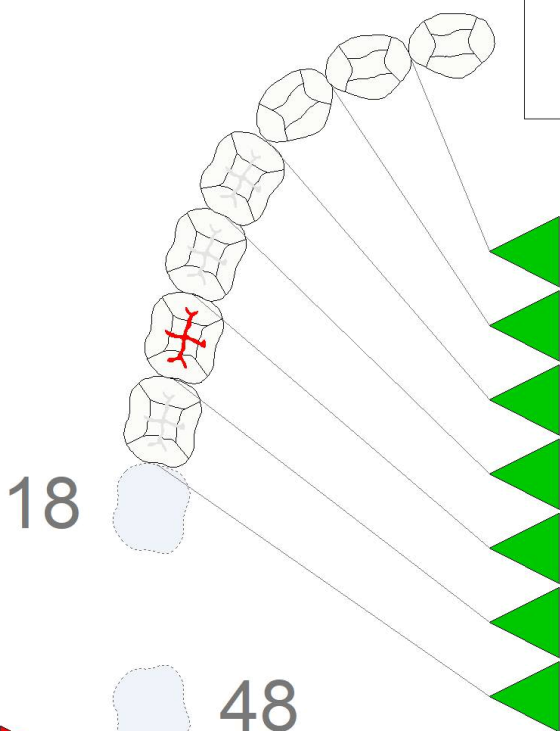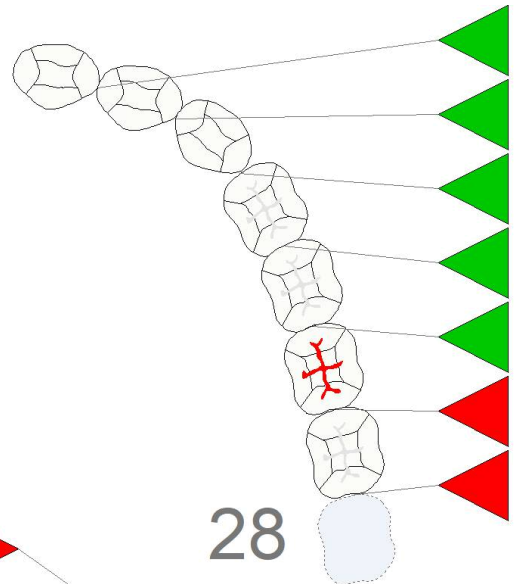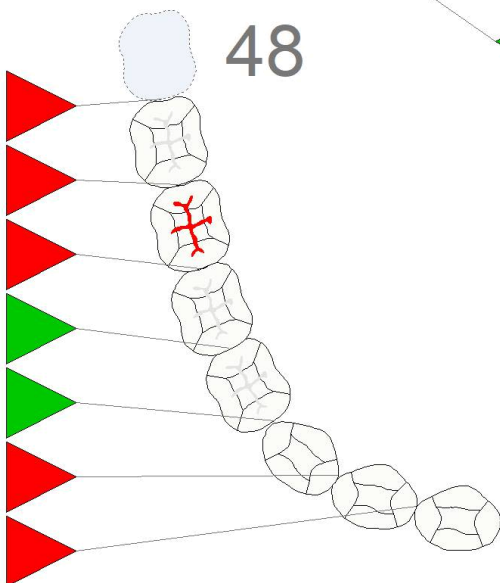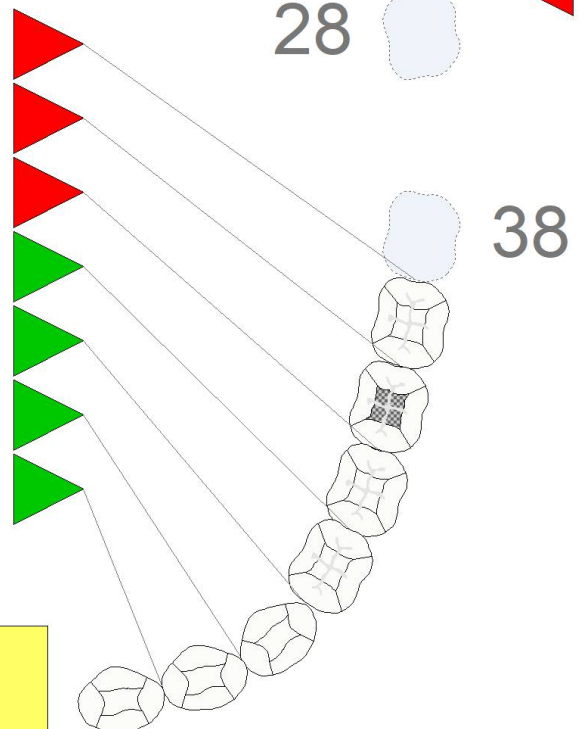

35,7%

Supplement: Supplementary file 1 [file ijms-26-01228-s001.zip › Supplementary materials/Table S1/Tables API, SBI, PSI/Sample _2/API.pdf]

# Zahnbefund

Probe: 2

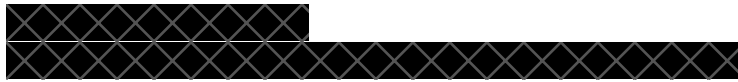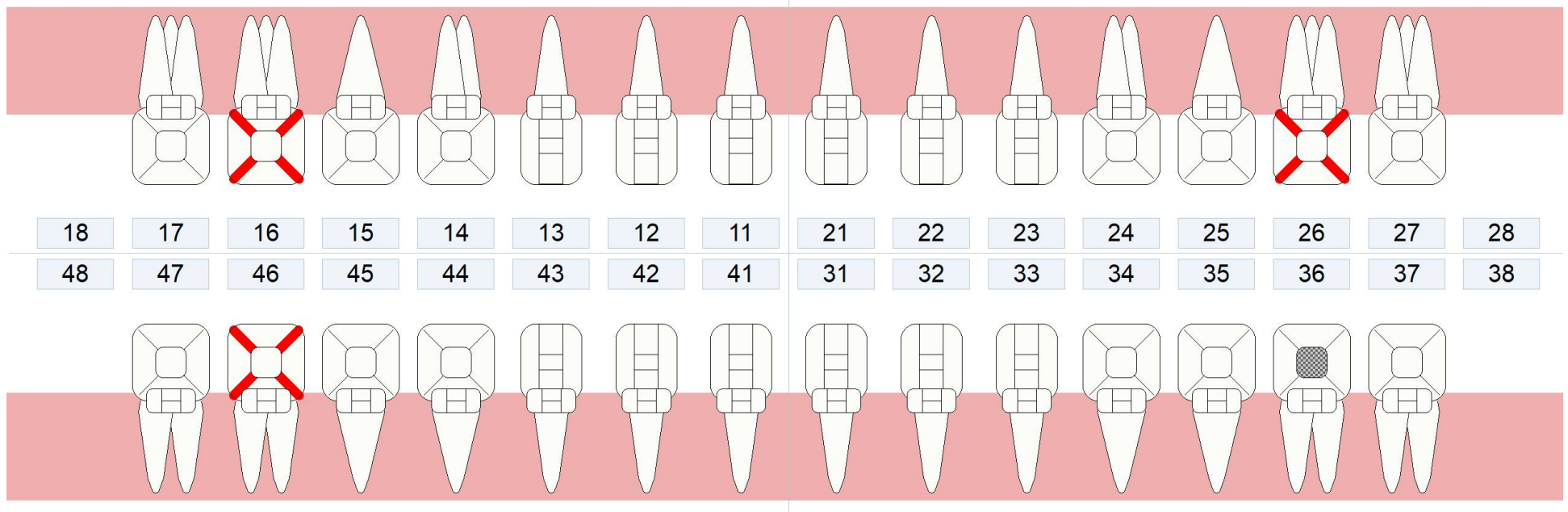

Supplement: Supplementary file 1 [file ijms-26-01228-s001.zip › Supplementary materials/Table S1/Tables API, SBI, PSI/Sample _2/Befund.pdf]

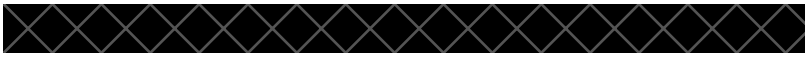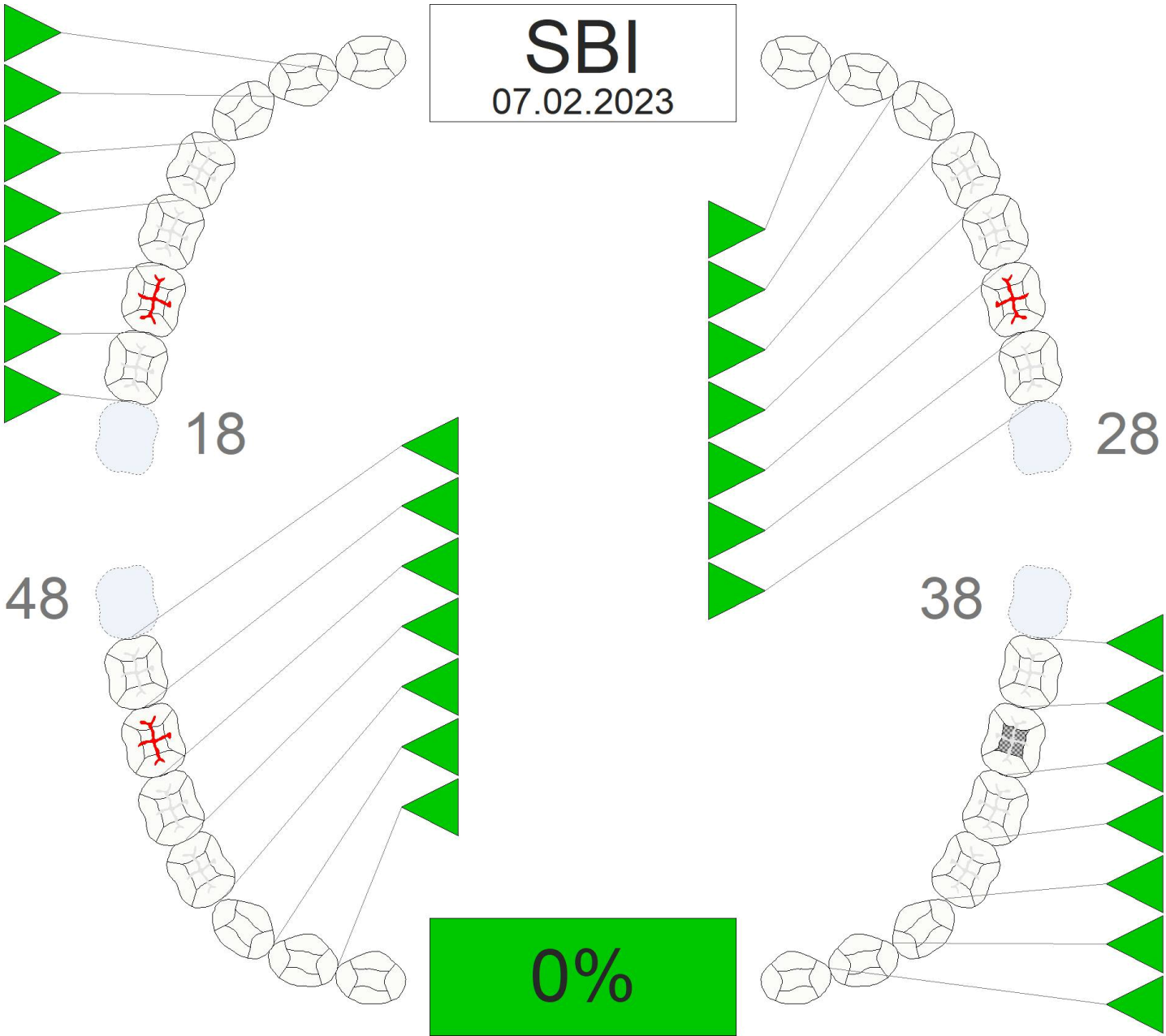

Supplement: Supplementary file 1 [file ijms-26-01228-s001.zip › Supplementary materials/Table S1/Tables API, SBI, PSI/Sample _2/SBI.pdf]

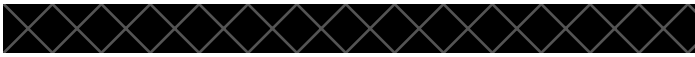

Probe: 3

API  
30.01.2023

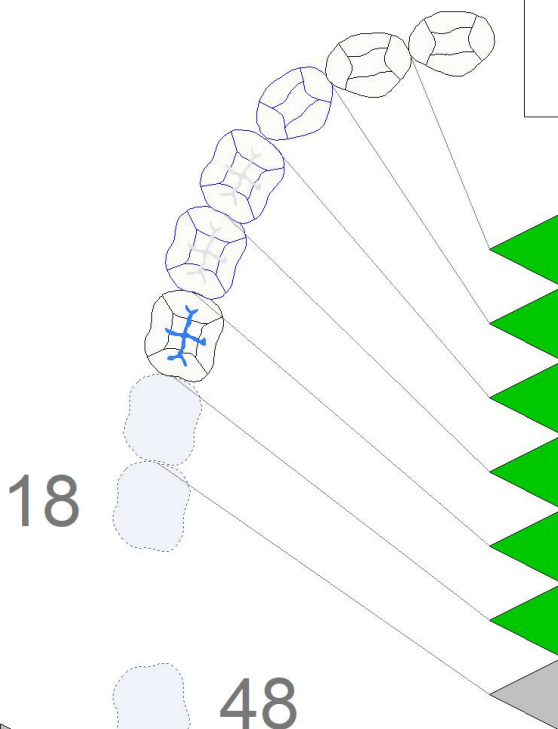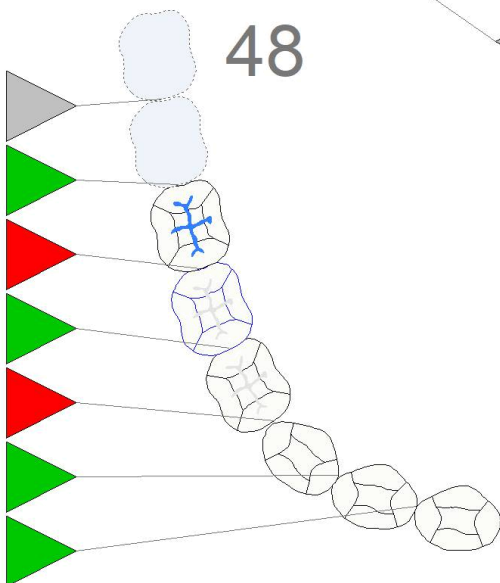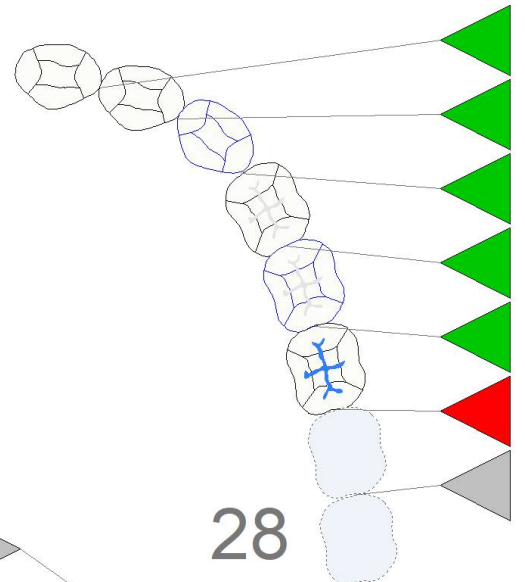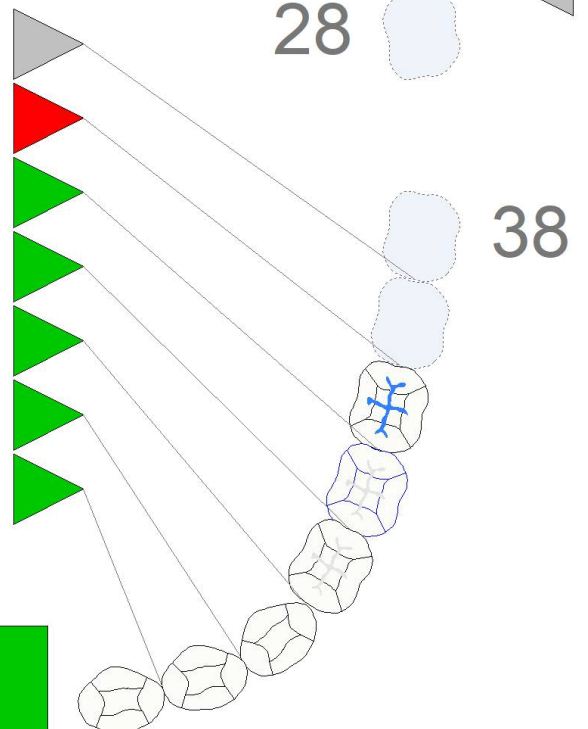

16,6%

Supplement: Supplementary file 1 [file ijms-26-01228-s001.zip › Supplementary materials/Table S1/Tables API, SBI, PSI/Sample _3/API.pdf]

# Zahnbefund

Probe: 3

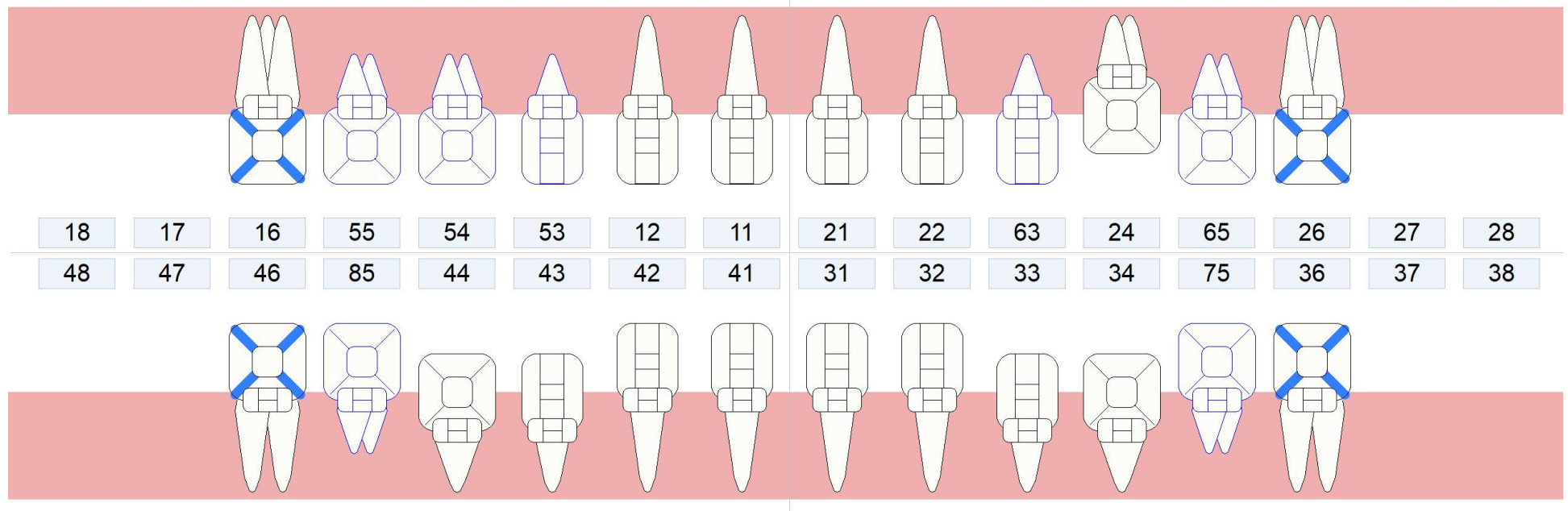

Supplement: Supplementary file 1 [file ijms-26-01228-s001.zip › Supplementary materials/Table S1/Tables API, SBI, PSI/Sample _3/Befund.pdf]

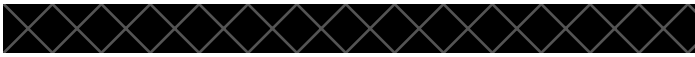

Probe: 3

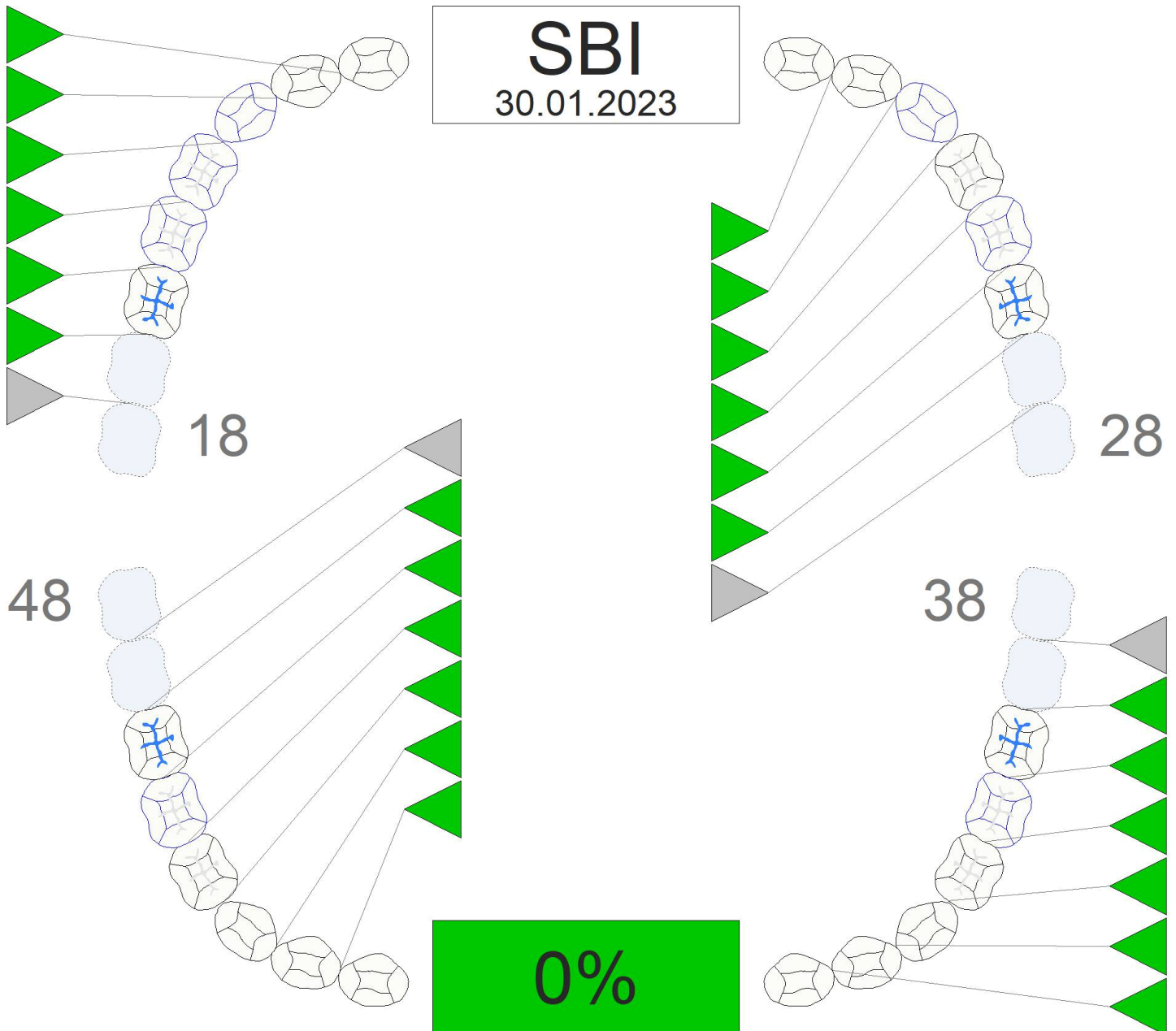

Supplement: Supplementary file 1 [file ijms-26-01228-s001.zip › Supplementary materials/Table S1/Tables API, SBI, PSI/Sample _3/SBI.pdf]

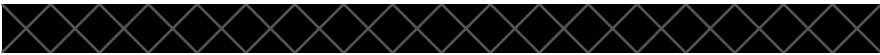

Probe: 4

API  
02.03.2023

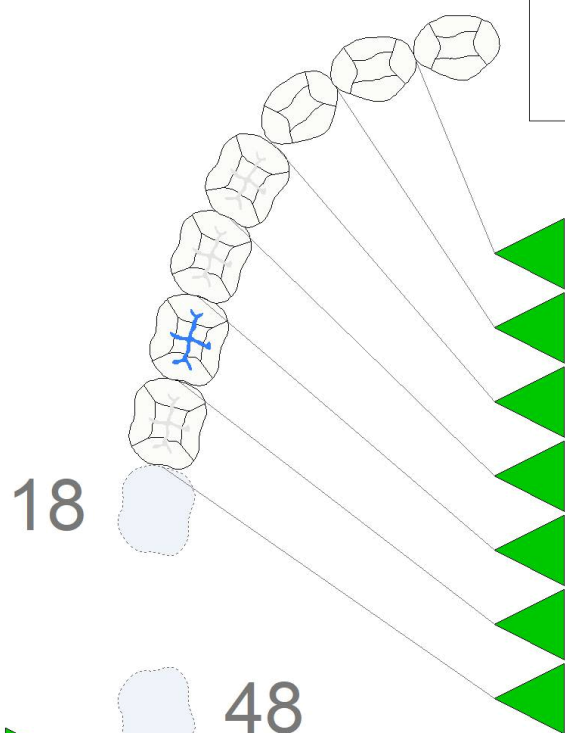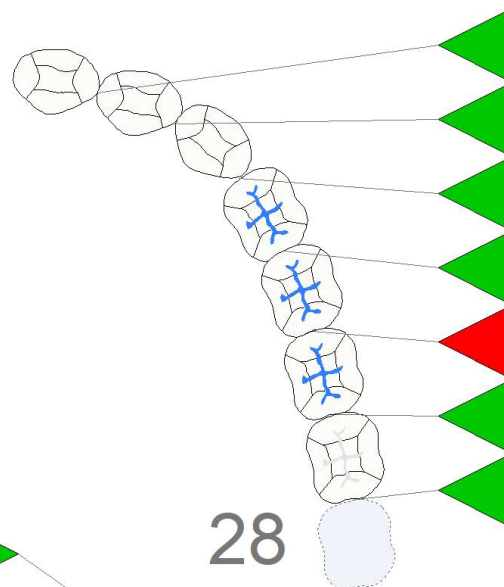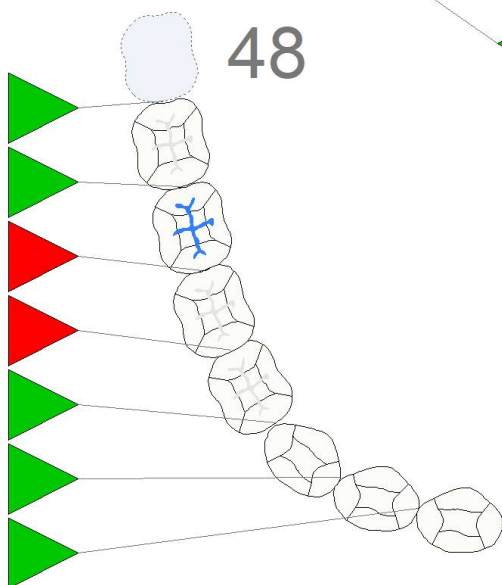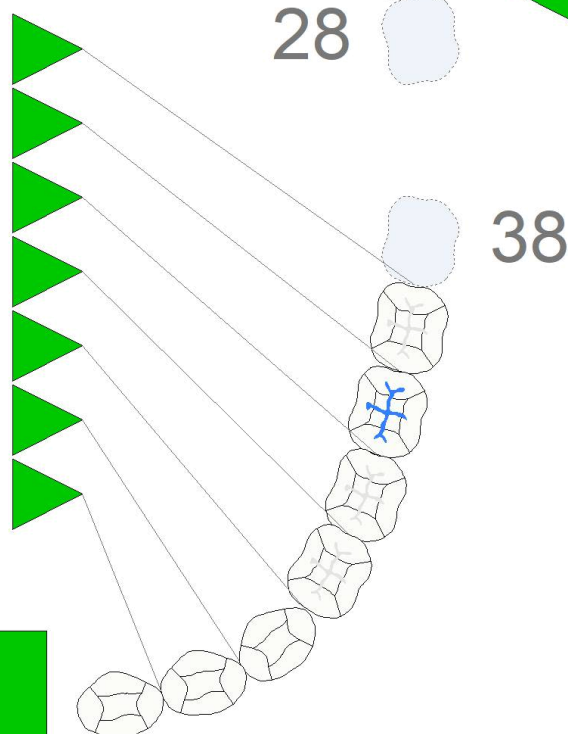

10,7%

Supplement: Supplementary file 1 [file ijms-26-01228-s001.zip › Supplementary materials/Table S1/Tables API, SBI, PSI/Sample _4/API.pdf]

## Probe: 4

Zahnbefund

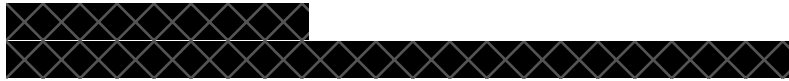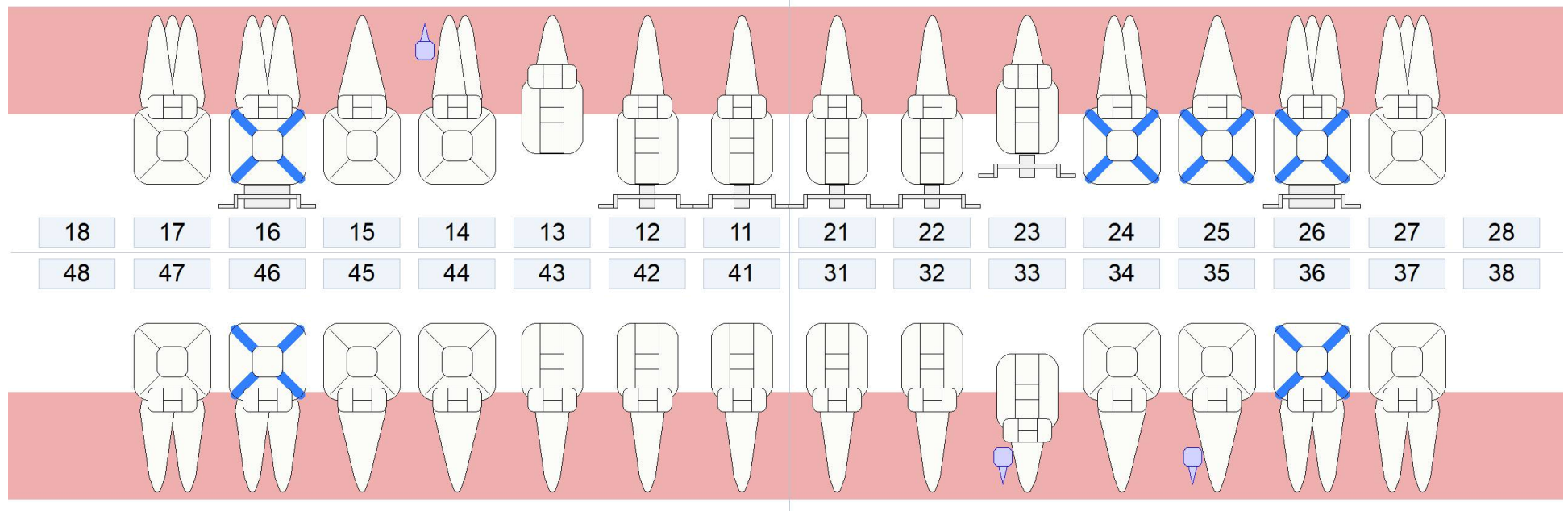

Supplement: Supplementary file 1 [file ijms-26-01228-s001.zip › Supplementary materials/Table S1/Tables API, SBI, PSI/Sample _4/Befund.pdf]

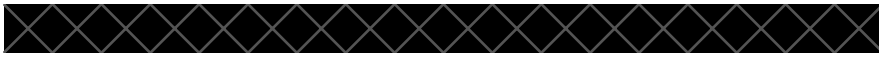

Probe: 4

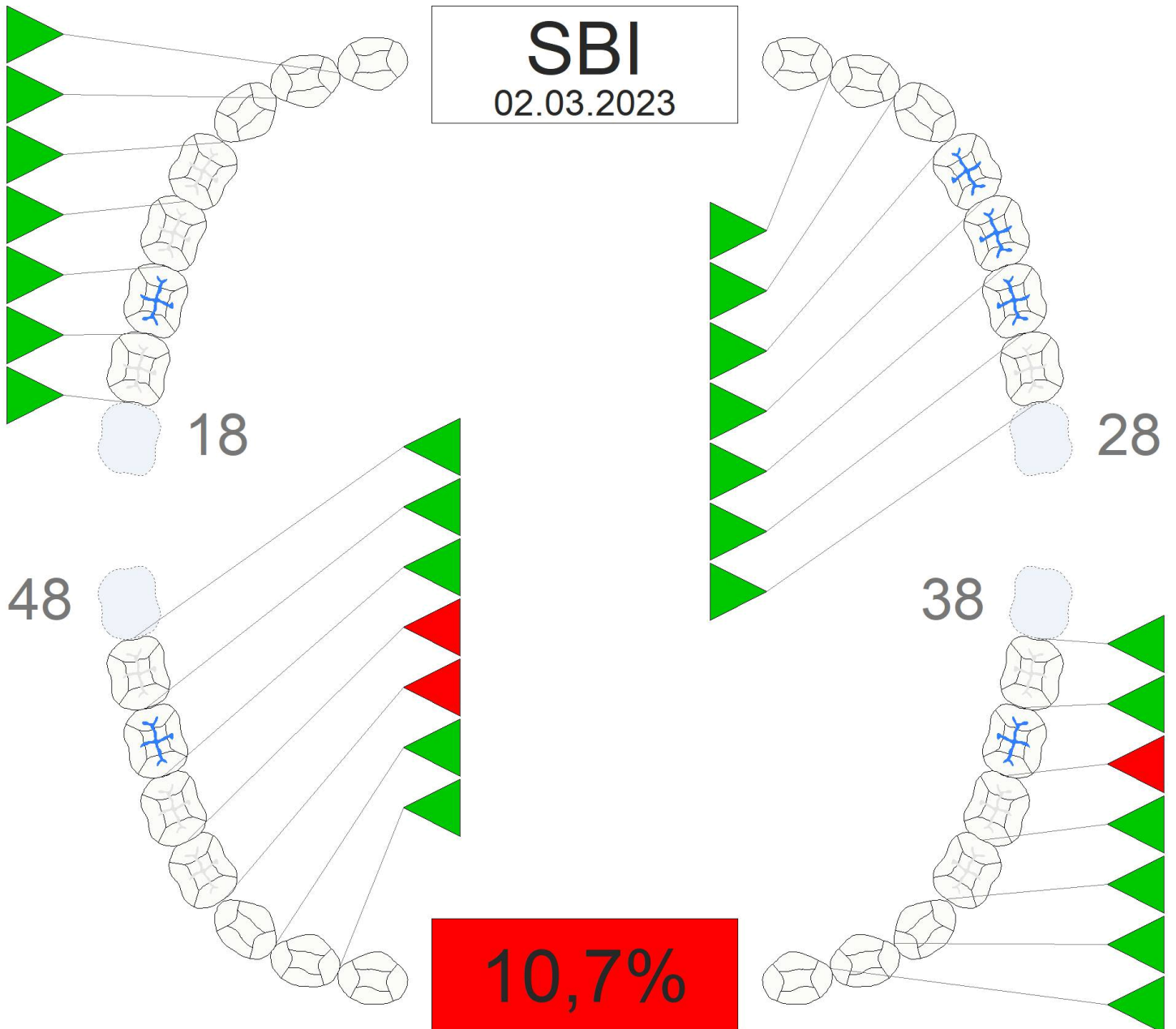

Supplement: Supplementary file 1 [file ijms-26-01228-s001.zip › Supplementary materials/Table S1/Tables API, SBI, PSI/Sample _4/SBI.pdf]

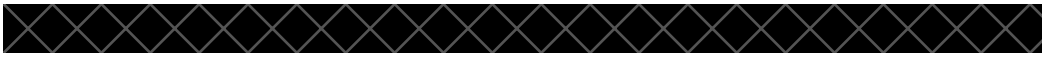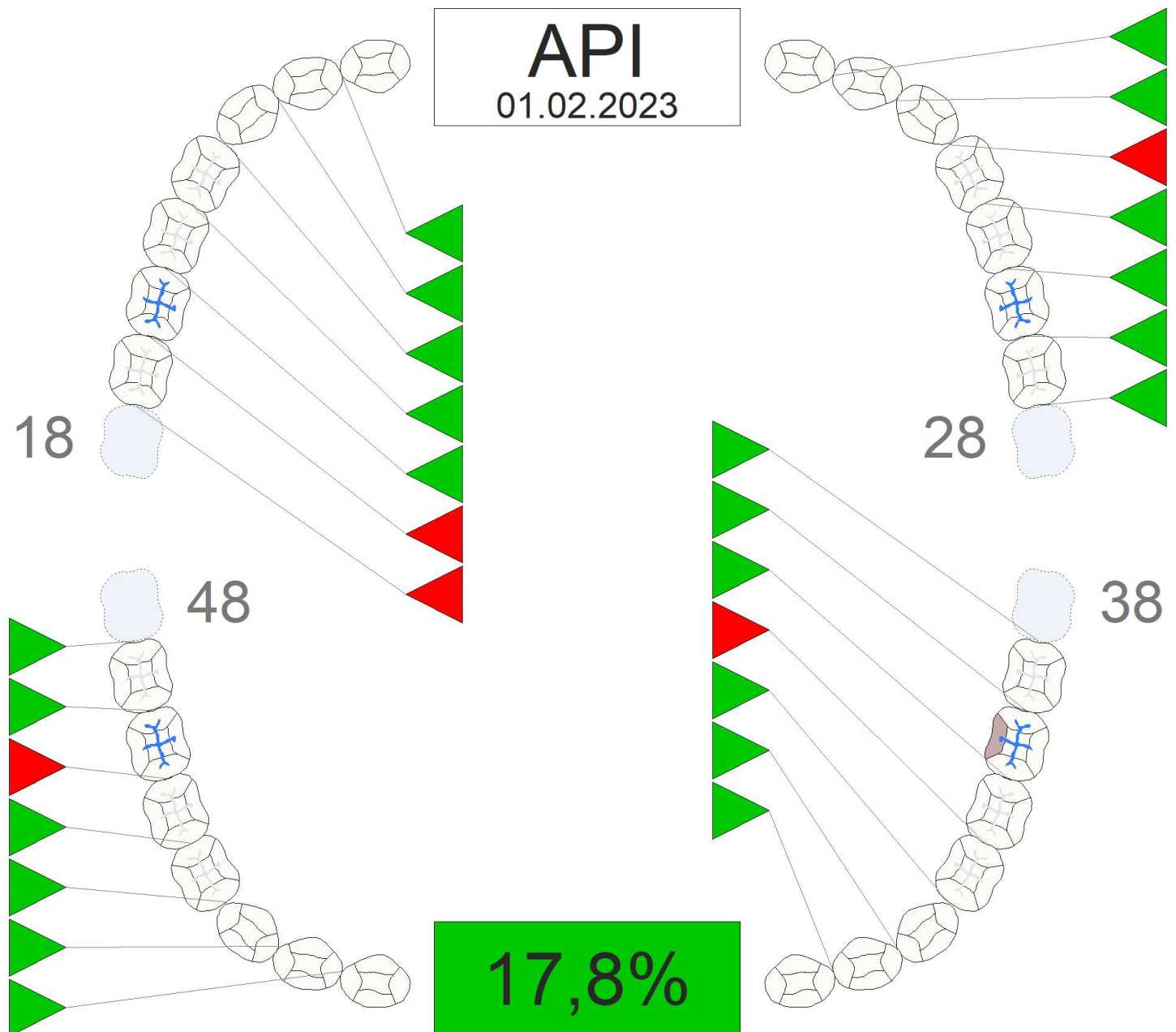

Supplement: Supplementary file 1 [file ijms-26-01228-s001.zip › Supplementary materials/Table S1/Tables API, SBI, PSI/Sample _5/API.pdf]

# Zahnbefund

Probe: 5

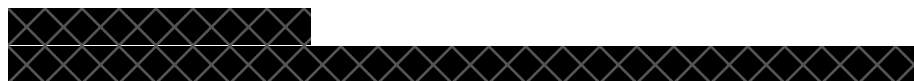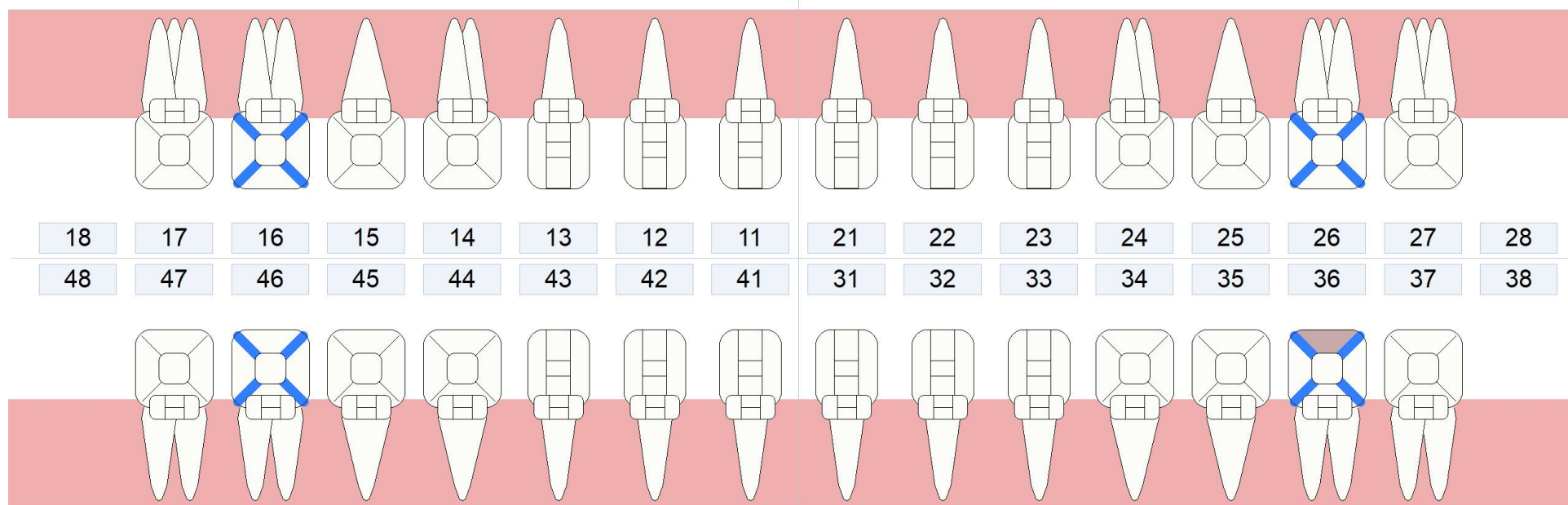

Supplement: Supplementary file 1 [file ijms-26-01228-s001.zip › Supplementary materials/Table S1/Tables API, SBI, PSI/Sample _5/Befund.pdf]

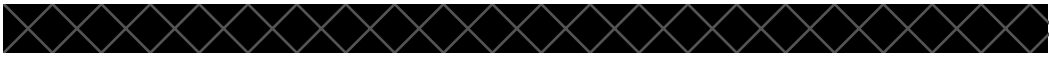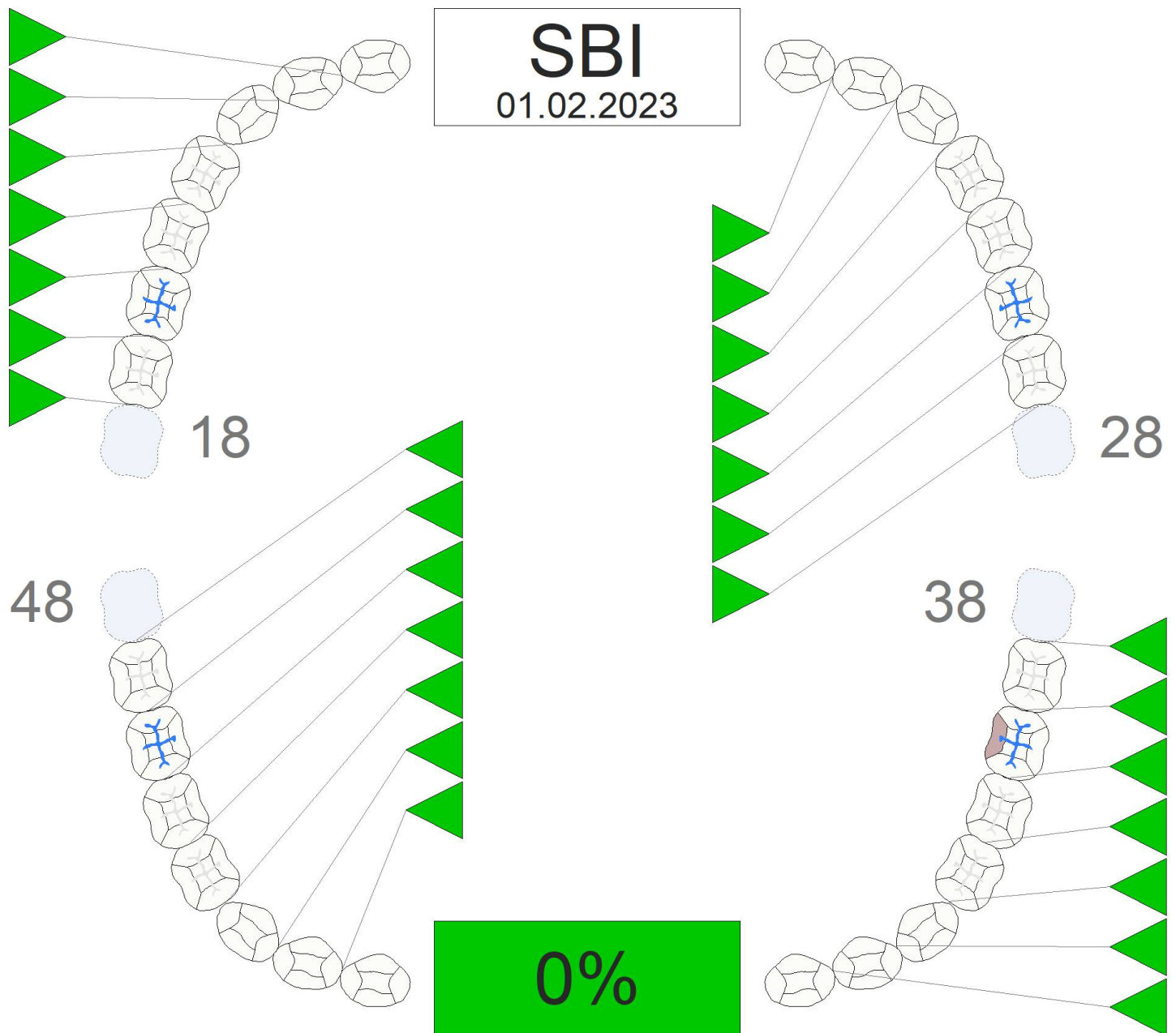

Supplement: Supplementary file 1 [file ijms-26-01228-s001.zip › Supplementary materials/Table S1/Tables API, SBI, PSI/Sample _5/SBI.pdf]

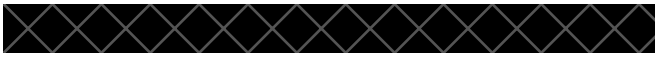

Probe: 6

API  
06.03.2023

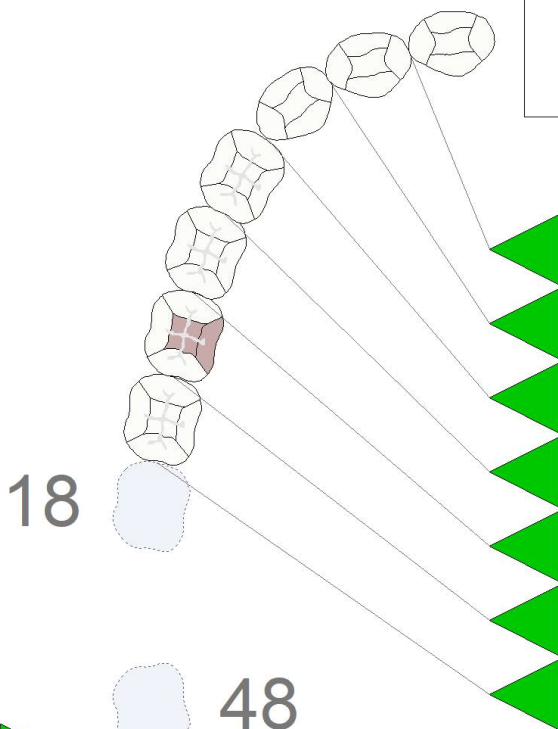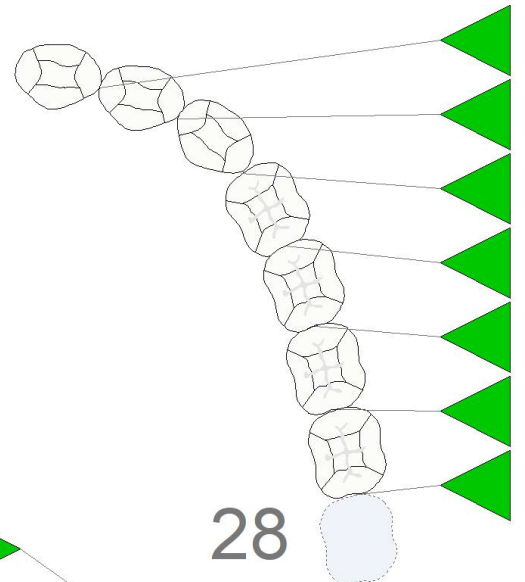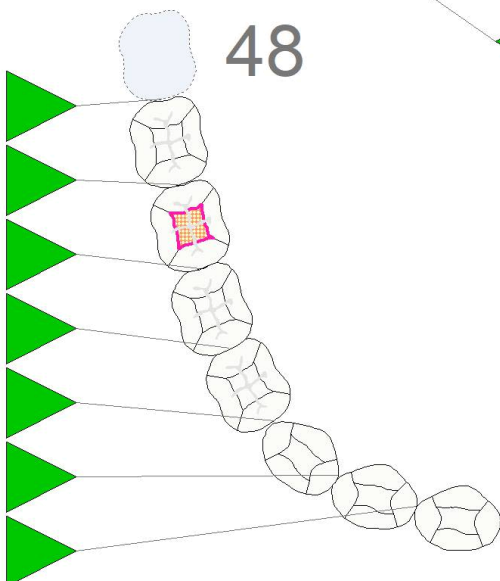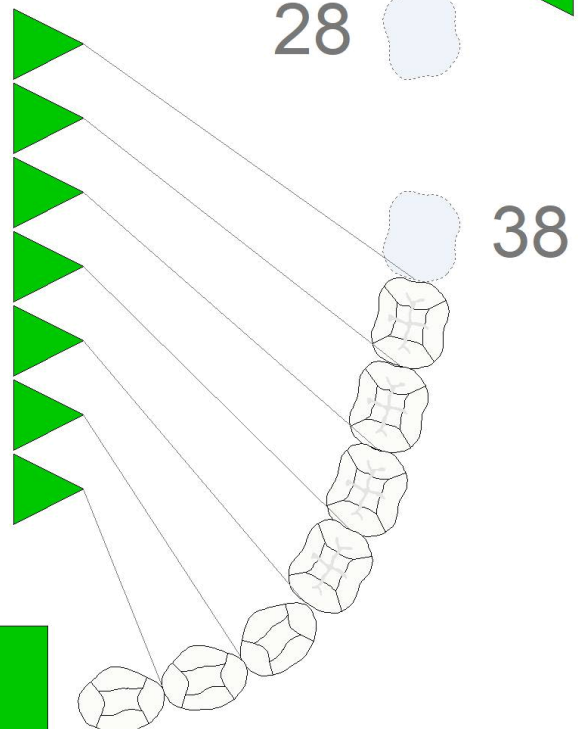

0%

Supplement: Supplementary file 1 [file ijms-26-01228-s001.zip › Supplementary materials/Table S1/Tables API, SBI, PSI/Sample _6/API.pdf]

Zahnbefund

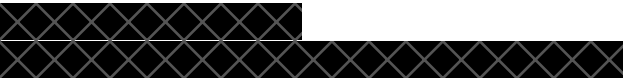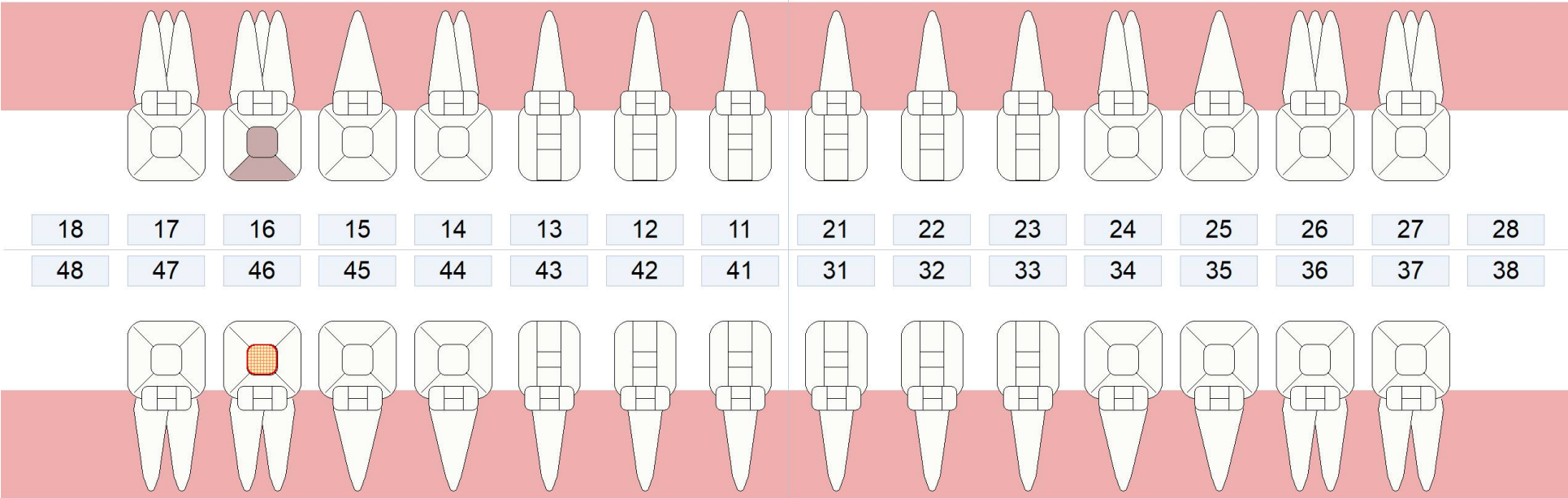

Supplement: Supplementary file 1 [file ijms-26-01228-s001.zip › Supplementary materials/Table S1/Tables API, SBI, PSI/Sample _6/Befund.pdf]

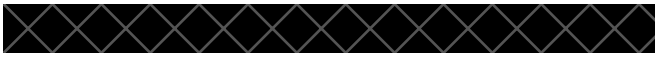

## Probe: 6

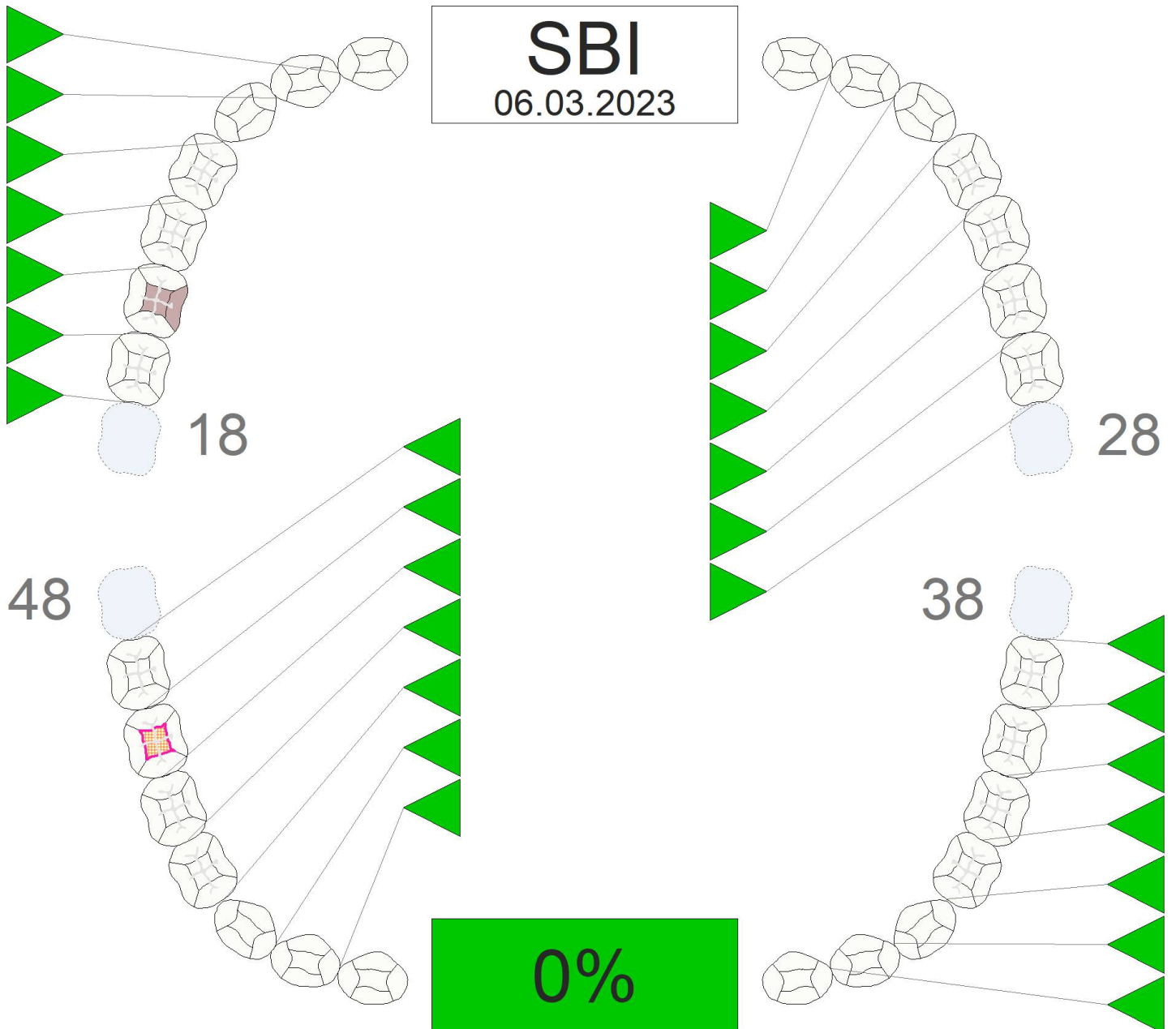

Supplement: Supplementary file 1 [file ijms-26-01228-s001.zip › Supplementary materials/Table S1/Tables API, SBI, PSI/Sample _6/SBI.pdf]

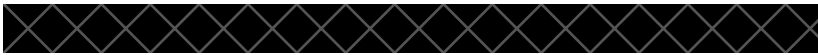

Probe: 7

API  
27.02.2023

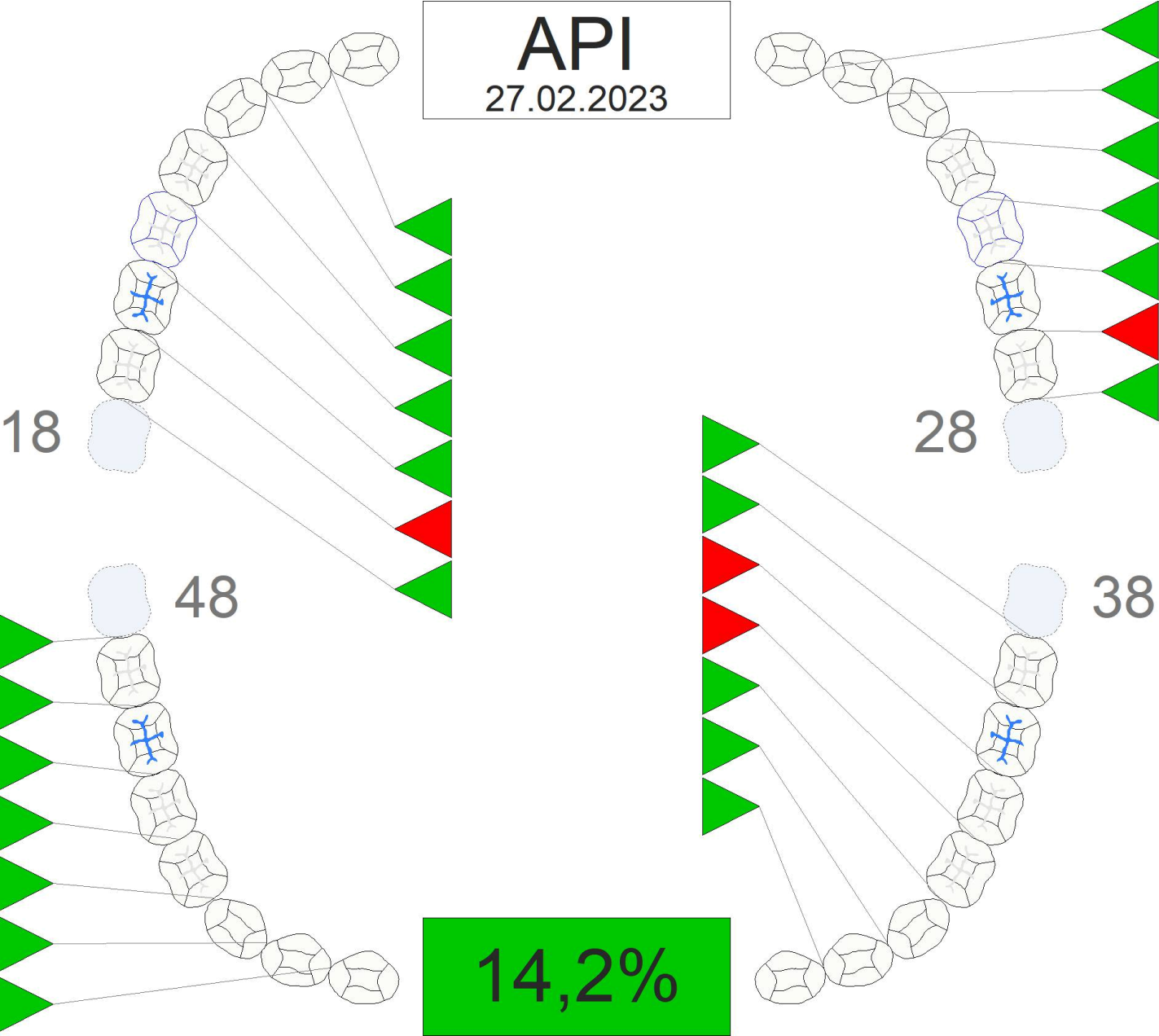

Supplement: Supplementary file 1 [file ijms-26-01228-s001.zip › Supplementary materials/Table S1/Tables API, SBI, PSI/Sample _7/API.pdf]

# Zahnbefund

Probe: 7

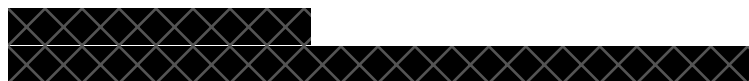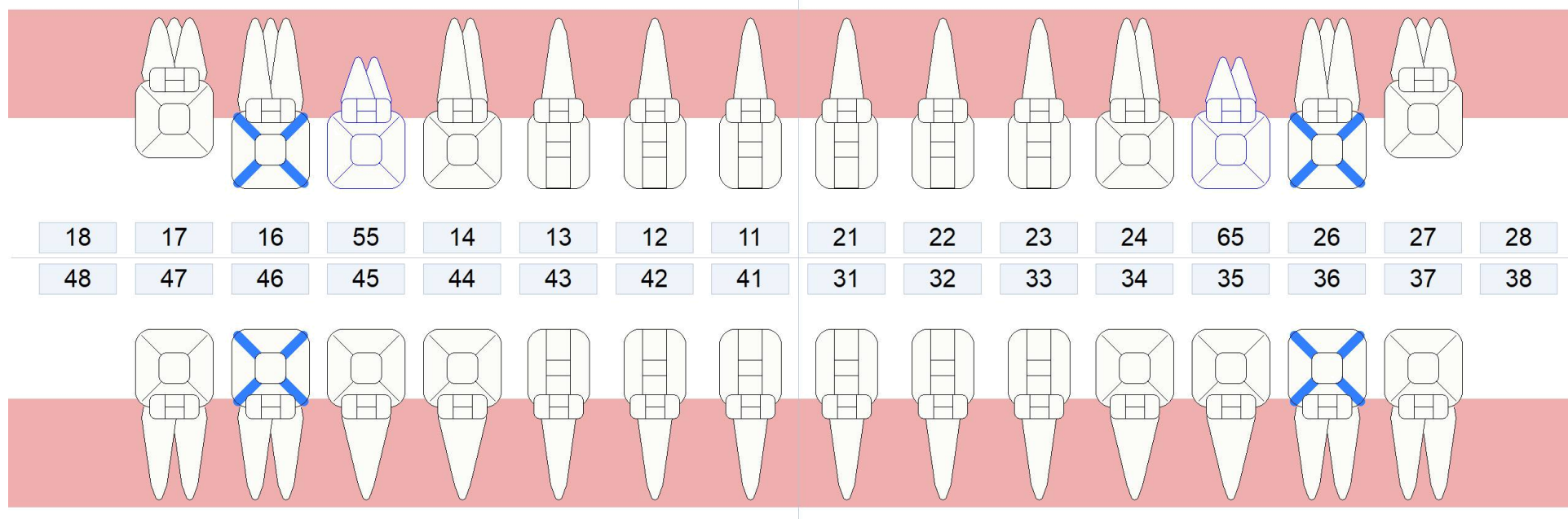

Supplement: Supplementary file 1 [file ijms-26-01228-s001.zip › Supplementary materials/Table S1/Tables API, SBI, PSI/Sample _7/Befund.pdf]

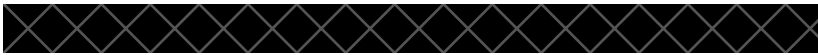

Probe: 7

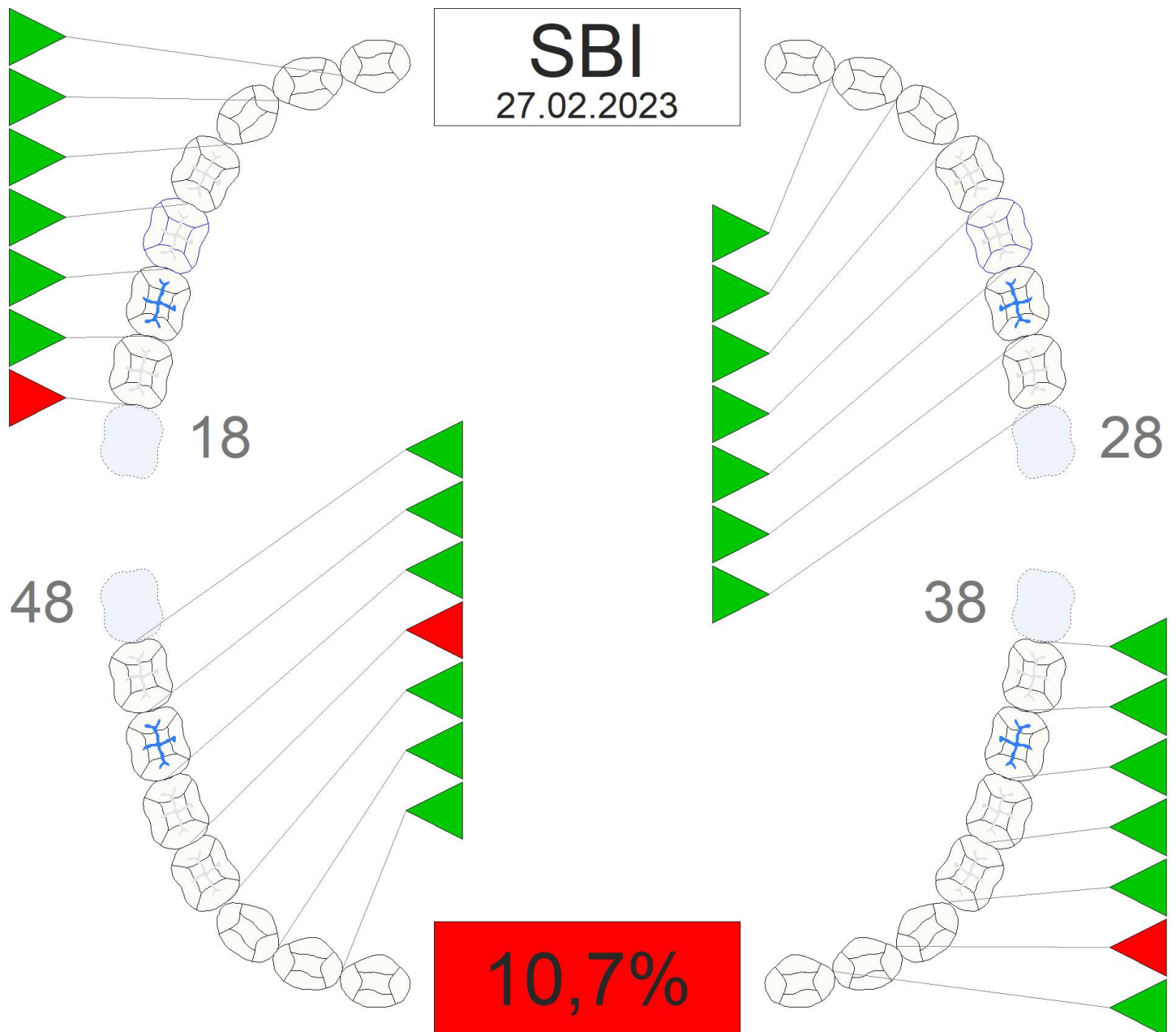

Supplement: Supplementary file 1 [file ijms-26-01228-s001.zip › Supplementary materials/Table S1/Tables API, SBI, PSI/Sample _7/SBI.pdf]

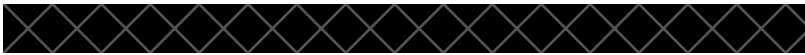

Probe: 8

API  
27.02.2023

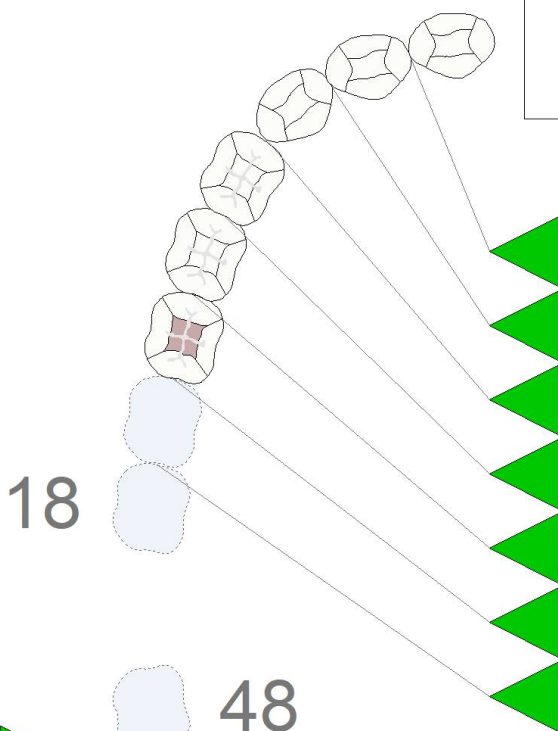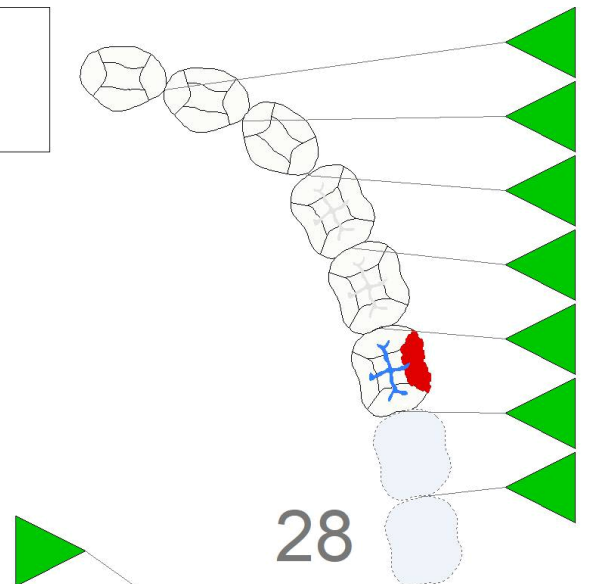

0%

Supplement: Supplementary file 1 [file ijms-26-01228-s001.zip › Supplementary materials/Table S1/Tables API, SBI, PSI/Sample _8/API.pdf]

| Age Group | Percentage |
|-----------|------------|
| 18-24     | ~22%       |
| 25-34     | ~32%       |
| 35-44     | ~20%       |
| 45-54     | ~18%       |
| 55-64     | ~15%       |
| 65-74     | ~12%       |
| 75-84     | ~8%        |
| 85+       | ~5%        |

## Probe: 8

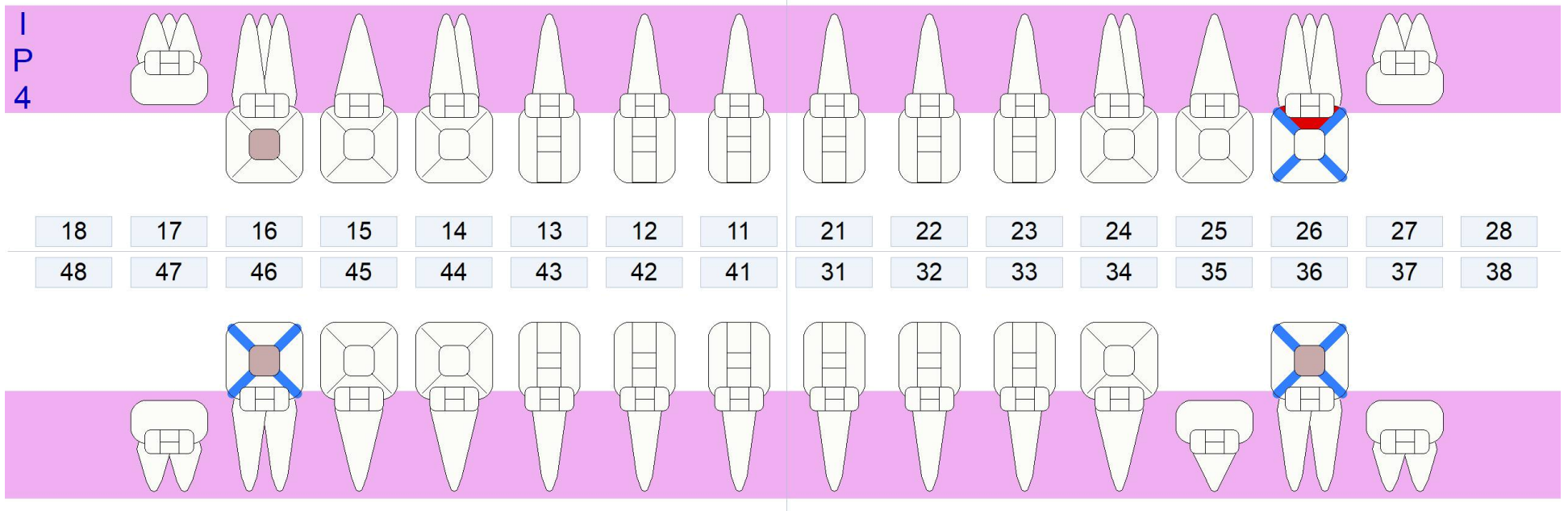

Supplement: Supplementary file 1 [file ijms-26-01228-s001.zip › Supplementary materials/Table S1/Tables API, SBI, PSI/Sample _8/Befund.pdf]

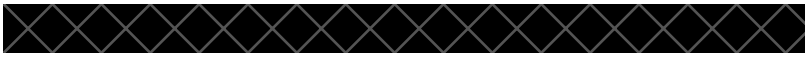

Probe: 8

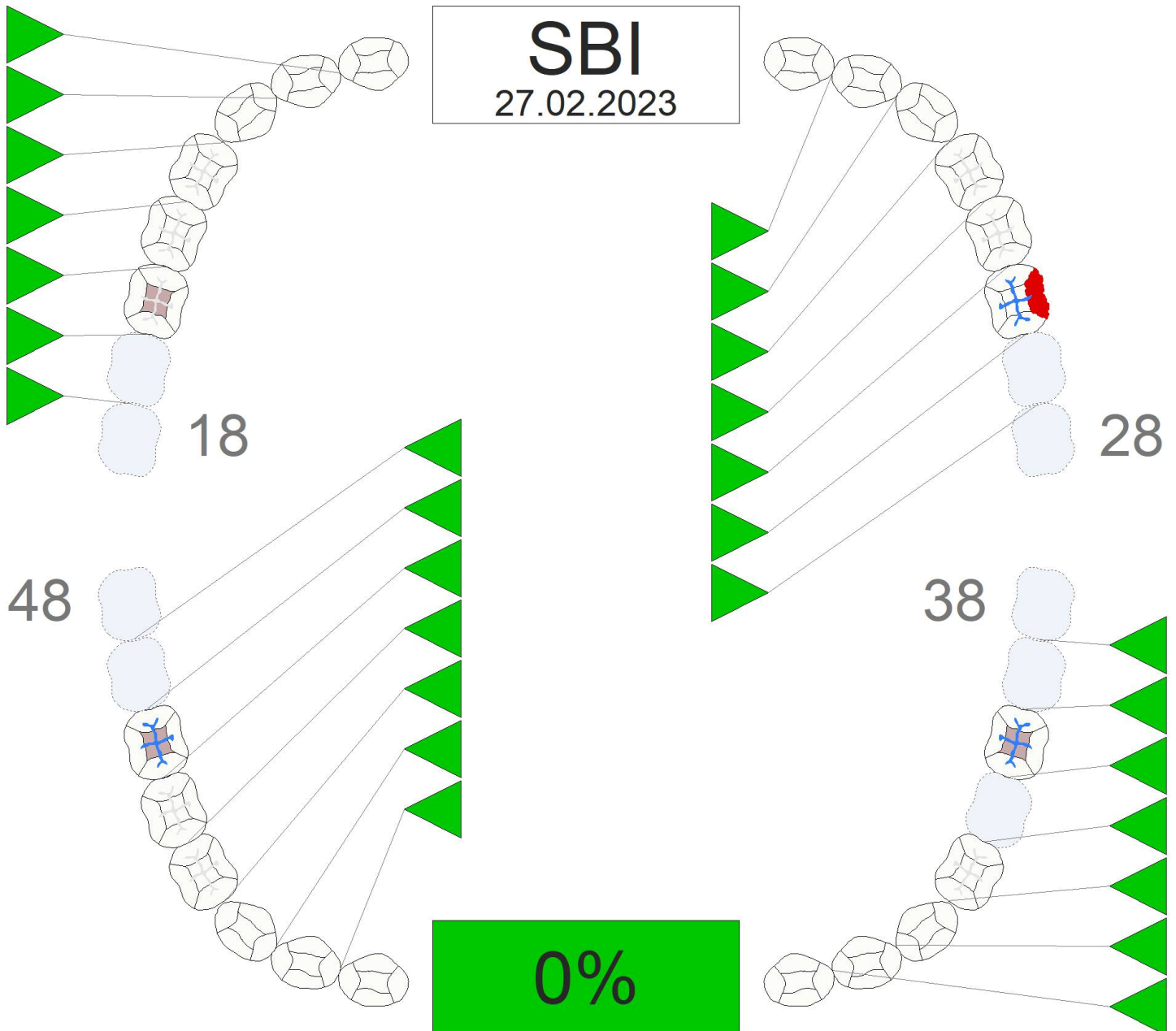

Supplement: Supplementary file 1 [file ijms-26-01228-s001.zip › Supplementary materials/Table S1/Tables API, SBI, PSI/Sample _8/SBI.pdf]

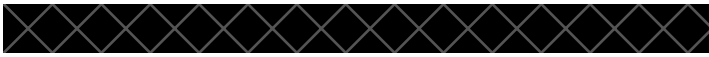

Probe: 9

API  
13.03.2023

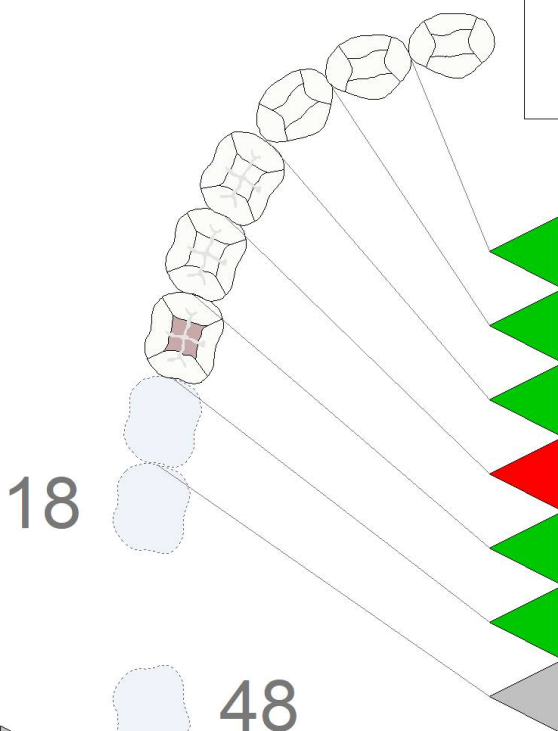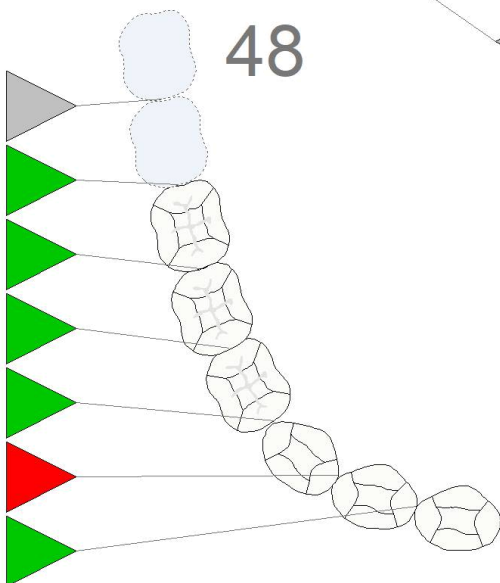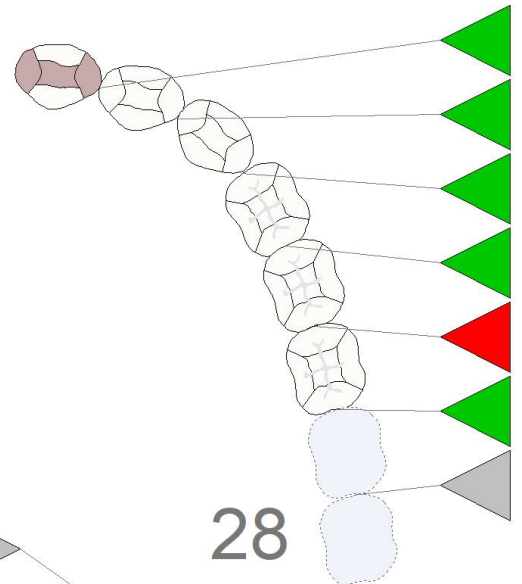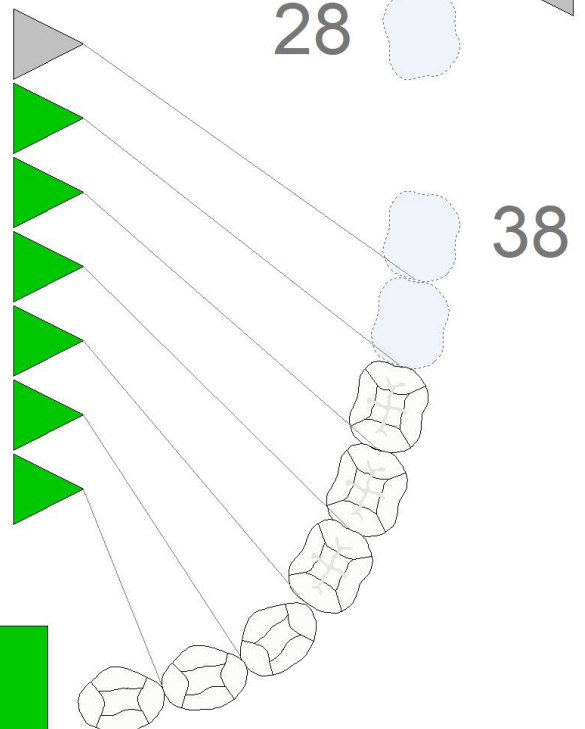

12,5%

Supplement: Supplementary file 1 [file ijms-26-01228-s001.zip › Supplementary materials/Table S1/Tables API, SBI, PSI/Sample _9/API.pdf]

# Zahnbefund

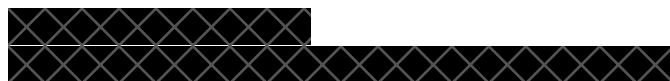

Probe: 9

| I<br>P<br>4 |    |    |    |    |    |    |    |    |    |    |    |    |    |    |    |    |
|-------------|----|----|----|----|----|----|----|----|----|----|----|----|----|----|----|----|
|             | 18 | 17 | 16 | 15 | 14 | 13 | 12 | 11 | 21 | 22 | 23 | 24 | 25 | 26 | 27 | 28 |
|             | 48 | 47 | 46 | 45 | 44 | 43 | 42 | 41 | 31 | 32 | 33 | 34 | 35 | 36 | 37 | 38 |
|             |    |    |    |    |    |    |    |    |    |    |    |    |    |    |    |    |

Supplement: Supplementary file 1 [file ijms-26-01228-s001.zip › Supplementary materials/Table S1/Tables API, SBI, PSI/Sample _9/Befund.pdf]

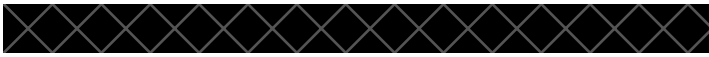

Probe: 9

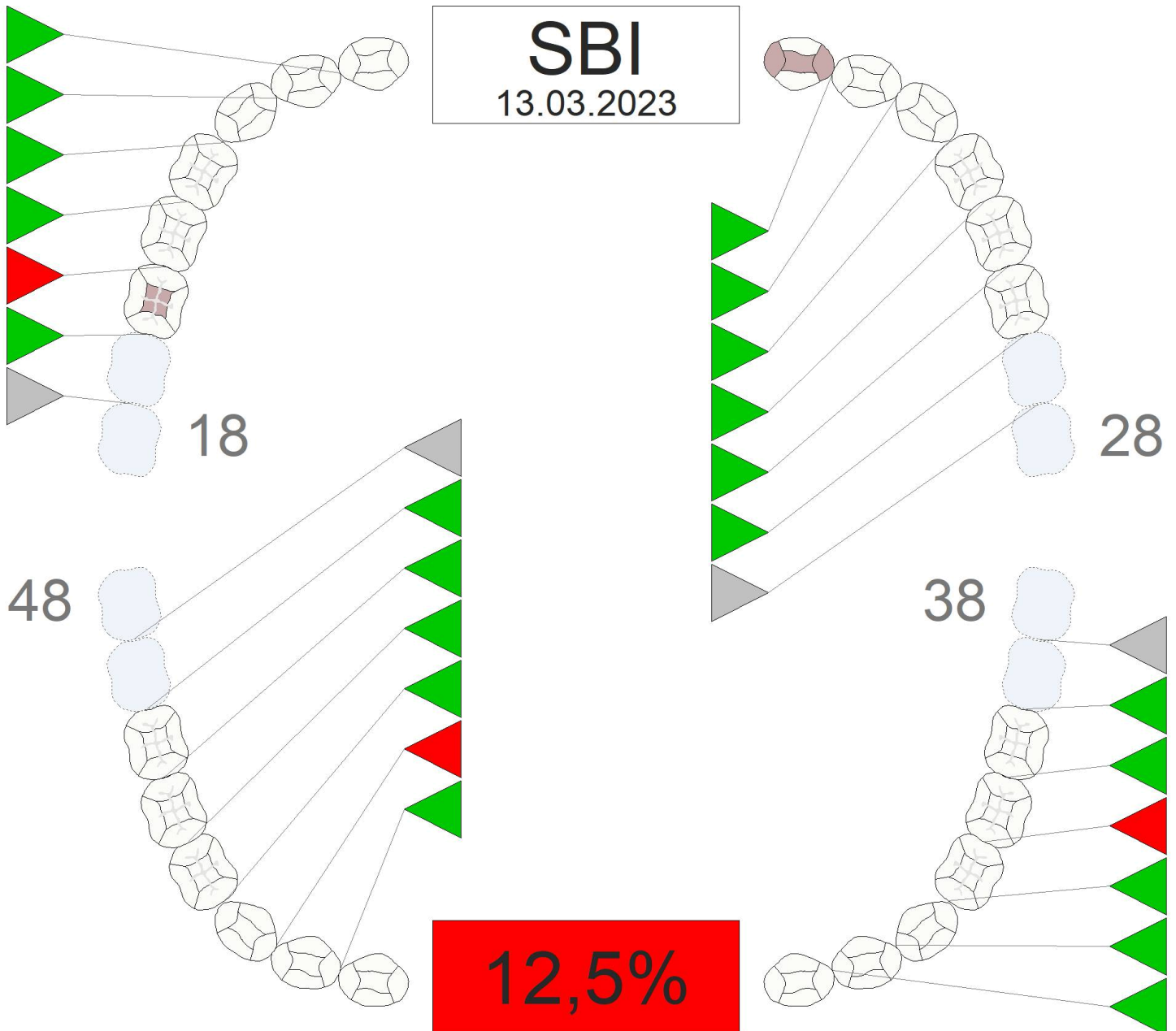

Supplement: Supplementary file 1 [file ijms-26-01228-s001.zip › Supplementary materials/Table S1/Tables API, SBI, PSI/Sample _9/SBI.pdf]
